# Mitochondrial matrix RTN4IP1/OPA10 is an oxidoreductase for coenzyme Q synthesis

**DOI:** 10.1038/s41589-023-01452-w

**Published:** 2023-10-26

**Authors:** Isaac Park, Kwang-eun Kim, Jeesoo Kim, Ae-Kyeong Kim, Subin Bae, Minkyo Jung, Jinhyuk Choi, Pratyush Kumar Mishra, Taek-Min Kim, Chulhwan Kwak, Myeong-Gyun Kang, Chang-Mo Yoo, Ji Young Mun, Kwang-Hyeon Liu, Kyu-Sun Lee, Jong-Seo Kim, Jae Myoung Suh, Hyun-Woo Rhee

**Affiliations:** 1 Department of Chemistry, Seoul National University, Seoul, Republic of Korea; 2 Graduate School of Medical Science and Engineering, KAIST, Daejeon, Republic of Korea; 3 School of Biological Sciences, Seoul National University, Seoul, Republic of Korea; 4 Center for RNA Research, Institute for Basic Science, Seoul, Republic of Korea; 5 Metabolism and Neurophysiology Research Group, KRIBB, Daejeon, Republic of Korea; 6 BK21 FOUR Community-Based Intelligent Novel Drug Discovery Education Unit, College of Pharmacy and Research Institute of Pharmaceutical Sciences, Kyungpook National University, Daegu, Korea; 7 Neural Circuit Research Group, Korea Brain Research Institute, Daegu, Republic of Korea; 8 School of Pharmacy, Sungkyunkwan University, Suwon, Korea

**Keywords:** Mass spectrometry, Metabolism, Enzyme mechanisms, Proteomics, Chemical tools

## Abstract

Targeting proximity-labeling enzymes to specific cellular locations is a viable strategy for profiling subcellular proteomes. Here, we generated transgenic mice (MAX-Tg) expressing a mitochondrial matrix-targeted ascorbate peroxidase. Comparative analysis of matrix proteomes from the muscle tissues showed differential enrichment of mitochondrial proteins. We found that reticulon 4-interacting protein 1 (RTN4IP1), also known as optic atrophy-10, is enriched in the mitochondrial matrix of muscle tissues and is an NADPH oxidoreductase. Interactome analysis and in vitro enzymatic assays revealed an essential role for RTN4IP1 in coenzyme Q (CoQ) biosynthesis by regulating the *O*-methylation activity of COQ3. *Rtn4ip1-*knockout myoblasts had markedly decreased CoQ_9_ levels and impaired cellular respiration. Furthermore, muscle-specific knockdown of *dRtn4ip1* in flies resulted in impaired muscle function, which was reversed by dietary supplementation with soluble CoQ. Collectively, these results demonstrate that RTN4IP1 is a mitochondrial NAD(P)H oxidoreductase essential for supporting mitochondrial respiration activity in the muscle tissue.

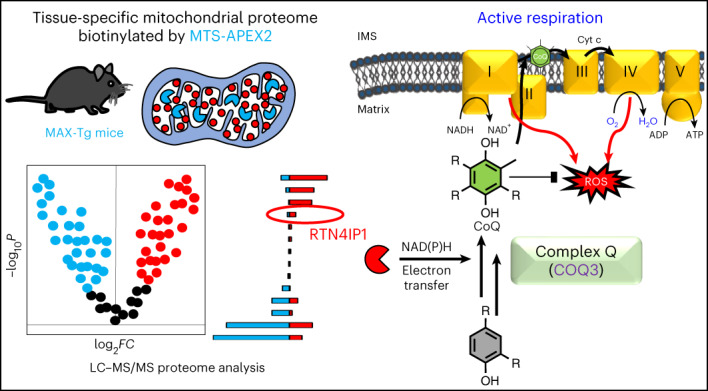

## Abstract (web summary)

The development of a transgenic mouse line that expresses mitochondrial matrix-targeted APEX2 combined with proteome analysis identified RTN4IP1, which serves as an NAD(P)H oxidoreductase required for respiration and CoQ biosynthesis.

## Main

In multicellular organisms, unique metabolic processes occur in different tissues, resulting in a tissue-specific proteome inventory^1,2^. As a central regulator of cellular metabolism, the mitochondrial proteome is expected to vary among tissues to meet tissue-specific metabolic demands. Imaging studies have shown substantial differences in the mitochondrial ultrastructure among tissues^3^, which are likely linked to distinct mitochondrial proteomes. For example, the oxidative phosphorylation (OXPHOS) complex is highly expressed in muscle tissues^4^. However, the precise details of the muscle-specific mitochondrial matrix proteome remain unclear.

Conventional approaches to identifying mitochondrial proteomes are largely based on subcellular fractionation followed by mass spectrometry (MS)^5–8^. However, even the mitochondrial proteome of the same muscle tissues shows discrepancies due to the inevitable contamination of nonmitochondrial proteins^9^. Furthermore, these approaches cannot provide submitochondrial spatial information; although this information can be inferred through a proteinase K digestion assay, this method suffers from technical artifacts^10–12^. As a result, information regarding the mitochondrial proteome from different tissues at the suborganelle level remains incomplete due to a lack of suitable methodology.

Engineered ascorbate peroxidase (APEX) allows for in situ biotinylation of the local proteome in multiple cell lines^10,13–17^. APEX-mediated proximity labeling results in the covalent biotinylation of tyrosine residues of endogenous proteins^10,13^, and liquid chromatography–tandem MS (LC–MS/MS) analysis of biotin-modified sites on endogenous proteins can reveal subcellular proteomes to which APEX is localized^10^. Using proximity-labeling techniques, we and our colleagues have successfully identified the submitochondrial proteome of the mitochondrial matrix^10,13^, intermembrane space^18,19^ and mitochondrial-associated membrane^20,21^ in the immortalized human cell line human embryonic kidney 293T (HEK293T).

Notably, the submitochondrial proteome information obtained using APEX in HEK293T cells^13,18^ has been incorporated into recent updates of the MitoCarta^22,23^ database. However, it is questionable whether the sum of the data from different biological systems and methods sufficiently reflects the diversity of in vivo physiological contexts, such as tissue-specific submitochondrial protein information. For example, some brown fat-specific intermembrane space proteins^24^ are not found in the MitoCarta database. Therefore, new analytical methods that can provide direct experimental evidence for tissue-specific submitochondrial proteomes are highly desirable.

Here, we extend the use of APEX labeling for profiling subcellular proteomes to whole-animal models. We established a transgenic (Tg) mouse model that expresses mitochondrial matrix-targeted engineered APEX2 (MAX), enabling the in situ biotinylation of mitochondrial matrix-localized proteins in different tissues. Using this MAX-Tg mouse model, we analyzed the muscle-specific mitochondrial matrix proteomes and identified reticulon 4-interacting protein 1 (RTN4IP1) as an oxidoreductase for coenzyme Q (CoQ) biosynthesis.

## Results

### MAX-Tg mice for mitochondrial matrix-specific labeling

**Fig. 1:**
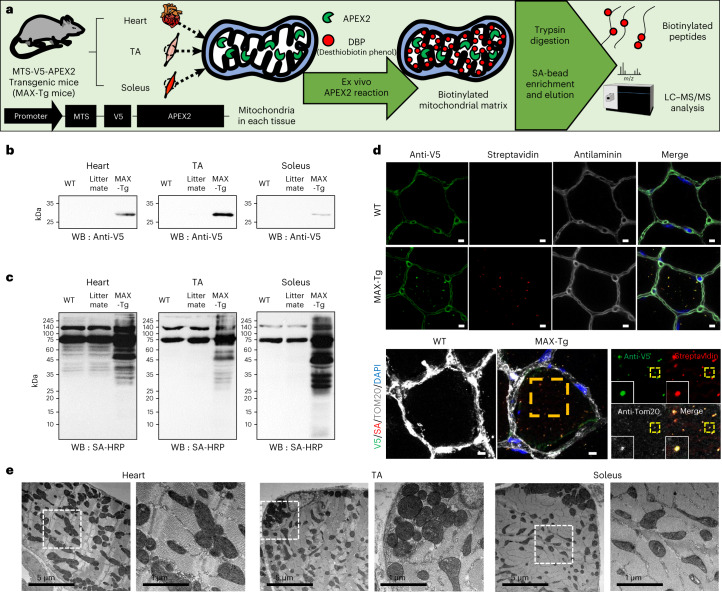
MTS-APEX2 transgenic (MAX-Tg) mice enable in situ profiling of mitochondrial matrix-specific proteomes. **a**, Scheme for tissue-specific mitochondrial matrix proteome mapping using MAX-Tg mice. **b**, Western blotting of MTS-V5-APEX2 (expected processed molecular weight 28 kDa) in WT, littermate and Tg mice. Representative images from three independent experiments are shown. **c**, Streptavidin (SA)-horseradish peroxidase western blotting of biotinylated proteins in WT, littermate and Tg mouse tissues after the APEX-mediated in situ biotinylation reaction (that is, DBP and H_2_O_2_ treatment). Representative images from three independent experiments are shown. **d**, Confocal microscopy imaging of MTS-APEX2 in situ biotinylation in the TA muscle of MAX-Tg mice. Scale bars, 5 µm. **e**, TEM of the mitochondrial matrix expression pattern of MTS-APEX2 in each muscle tissue of MAX-Tg mice. Source data

To generate transgenic mice with constitutive expression of APEX2 in the mitochondrial matrix, we localized APEX2 to the mitochondrial matrix by fusing it with the mitochondrial-targeting sequence (MTS) from cytochrome C oxidase subunit 4I1 (COX4I1) (Fig. 1a). Immunoblotting of desthiobiotin-phenol (DBP)-labeled muscle tissues confirmed the expression (V5) and functionality (horseradish peroxidase-conjugated streptavidin) of MTS-V5-APEX2 (Fig. 1b,c). Immunofluorescence imaging revealed specific V5 signals within the muscle myofibers of MAX-Tg mice, overlapping with TOM20 and streptavidin signals (Fig. 1d).

Transmission electron microscopy (TEM) was conducted to assess whether MTS-V5-APEX2 specifically targeted the mitochondrial matrix compartment, using 3,3′-diaminobenzidine (DAB) to stain by APEX in fixed tissues^25^, which confirmed the specific targeting of APEX2 to the mitochondrial matrix (Fig. 1e). The aligned cristae structure stained with MTS-APEX2 was observed in the heart and each skeletal muscle, aligning with the morphology of mitochondria in the muscle^26^. These data indicated that MTS-V5-APEX2 is expressed in the MAX-Tg mouse muscle and shows proximity-labeling activity in the mitochondrial matrix of muscle tissues.

### Divergent matrix proteome between mouse and human models

**Fig. 2:**
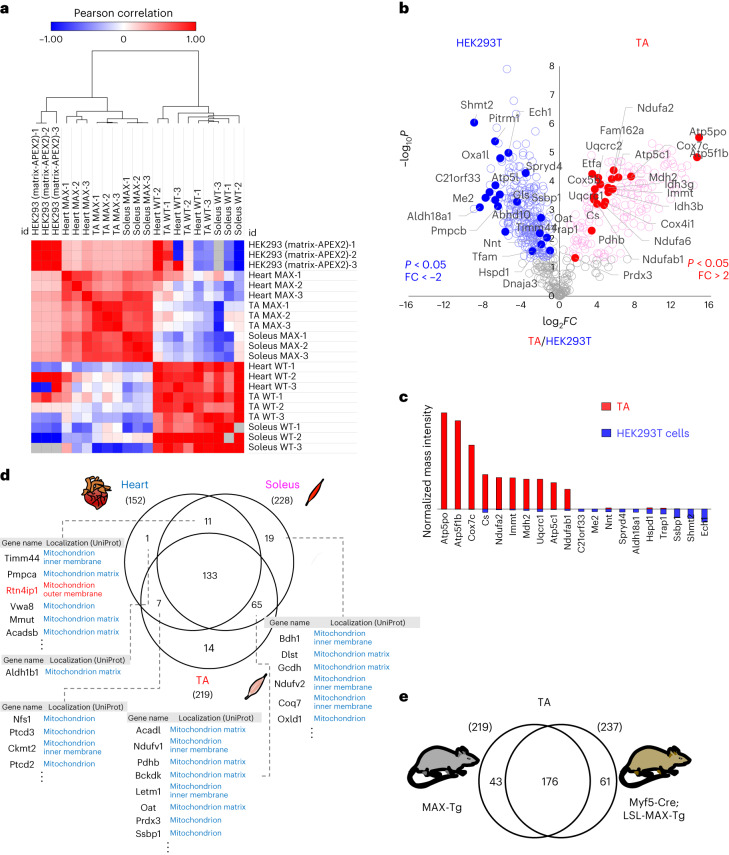
MTS-APEX2 transgenic (MAX-Tg) mice resolve distinct matrix proteomes of different muscle tissues. **a**, Heatmap of correlations between mass signal intensities of each replicate sample from WT mice, MAX-Tg mice and HEK293T cells (heart, TA, soleus, HEK293T cells stably expressing MTS-APEX2). Pearson correlation coefficients were calculated from each comparison. Hierarchical clustering was performed based on Pearson correlation coefficients. **b**, Volcano plot of the DBP-labeled proteome labeled by MTS-APEX2 in HEK293T cells (left) versus the TA muscle (right) from MAX-Tg mice. Statistical significance against the fold change revealed significantly different proteins between the HEK293T and TA muscle proteomes. The top 20 DBP-labeled proteins based on the normalized mass intensities in each sample are marked with filled circles with their gene names (see Supplementary Data 2 for detailed information). **c**, Top ten abundant DBP-labeled proteins labeled by MTS-APEX2 in TA tissue or HEK293T cells based on the normalized mass intensity. **d**, Venn diagram of identified mitochondrial matrix proteins from the heart, TA and soleus tissues. Representative proteins are shown with the current subcellular information in UniProt (see Supplementary Data 3 for detailed information). **e**, Venn diagram showing the overlap in MTS-APEX2-labeled mitochondrial protein identification between genetically different Tg mouse models (MAX-Tg mice and Myf5-Cre;LSL-MAX-Tg mice). See Supplementary Data 5 for detailed information.

**Extended Data Fig. 1:**
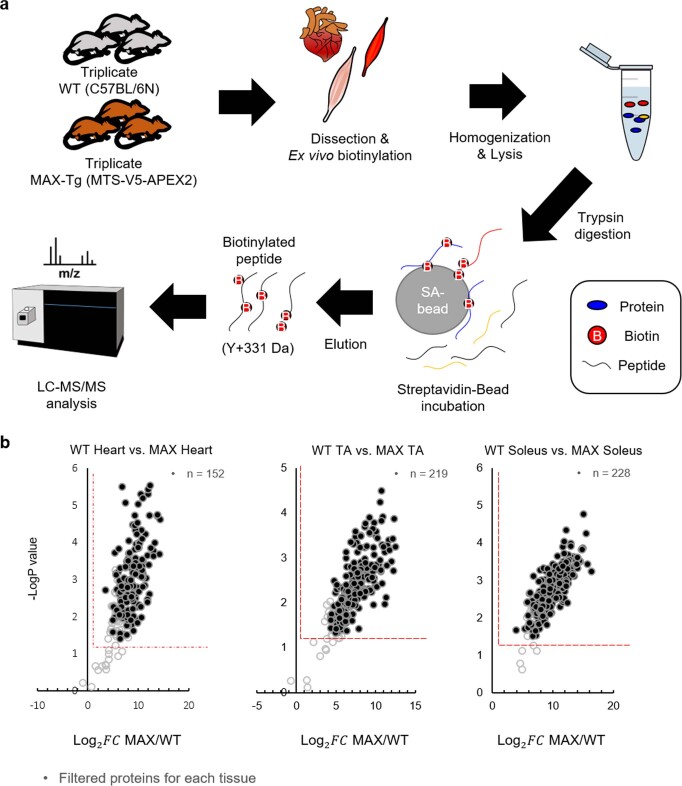
Experimental scheme to profile the mitochondrial matrix proteomes using mouse muscle tissues of MAX-Tg mice. (**a**) Scheme of LC-MS/MS analysis using MAX-Tg mice and Spot-ID methodology. (**b**) Volcano plot for the DBP-labeled proteome of each muscle tissue from WT mice (left) vs. MAX-Tg mice (right). Both samples were treated with the same amount of DBP and H2O2 prior to mass sampling. The cut-off for the mitochondrial matrix proteins was P < 0.05 and fold change (FC) > 2. See Supplementary Data 3 for detailed information.

Next, we performed ex vivo DBP labeling of Tg mouse tissues and identified muscle-specific mitochondrial matrix proteins using LC–MS/MS-based quantitative proteomic analysis (Extended Data Fig. 1a). We used the super-resolution proximity-labeling approach, which detects biotinylated sites at a single amino acid residue level^10^. Hundreds of DBP-labeled peptides were identified consistently using MTS-V5-APEX2 labeling (Fig. 2a and Supplementary Data 1). Distinct clusters were evident even between the tibialis anterior (TA) and soleus muscles, revealing the ability of MAX-Tg to differentiate the mitochondrial matrix proteome among various muscle groups.

**Extended Data Fig. 2:**
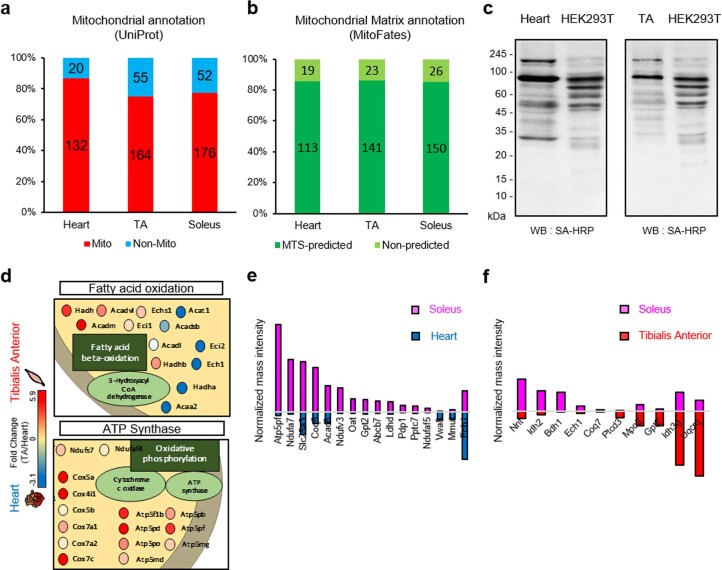
Asymmetrical DBP-labeled mitochondrial matrix proteins detected by MTS-V5-APEX2 in the TA and heart muscles of MAX-Tg. (**a**) Mitochondrial distribution or matrix of the DBP-labeled proteins by MTS-APEX2 in each muscle tissue of MAX-Tg mice. Mitochondrial proteins were classified by annotation of their subcellular localization in UniProt. (**b**) Mitochondrial matrix distribution of the DBP-labeled proteins by MTS-APEX2 among mitochondrial annotated proteins in indicated muscle tissues of MAX-Tg mice. Mitochondrial matrix proteins were classified with MitoFates prediction. (**c**) Western blots of biotinylated proteins in MAX-Tg mouse tissues and HEK293T cells expressing MTS-V5-APEX2 after the APEX-mediated in situ biotinylation reaction (that is, DBP and H2O2 treatment). Representative images from three independent experiments are shown. (**d**) Schematic representation of tissue-enriched mitochondrial proteins with similar metabolic functions in the TA muscle and heart. Heart- and TA-enriched proteins are colored in light blue and pink, respectively. All proteins are color-coded to reflect the fold change of the normalized mass intensity of DBP-labeled peptides in the TA muscle and the heart. Annotations with function and complex are based on the UniProt and CORUM databases. An expanded figure of tissue-enriched mitochondrial proteins (TA vs. heart) is shown in Extended Data Fig. 2f. See Supplementary Data 3 for detailed information. (**e**) Normalized mass intensities of asymmetrically DBP-labeled mitochondrial matrix proteins by MTS-APEX2 in the heart and soleus muscle. (**f**) Normalized mass intensities of asymmetrically DBP-labeled mitochondrial matrix proteins by MTS-APEX2 in the TA and soleus muscles. Source data

Using our filtering criteria (*P* < 0.05, fold change (FC) > 2) on MAX-Tg muscle samples against negative wild-type (WT) controls, we compiled a list of the MTS-V5-APEX2-labeled proteome for each muscle sample, comprising 152, 219 and 228 proteins for the heart, TA and soleus, respectively (Extended Data Fig. 1b). As expected, a substantial portion of the MTS-V5-APEX2-labeled proteome (73.6%; 184 of 250 total proteins) had a mitochondrial annotation in UniProt (Extended Data Fig. 2a). We also used MitoFates^27^ to verify whether the filtered proteins were annotated as mitochondrial matrix proteins, demonstrating that the MTS-V5-APEX2-labeled mitochondrial proteome was highly enriched (85.3%) with the predicted MTS (Extended Data Fig. 2b). These data demonstrated that MAX-Tg mice selectively label mitochondrial matrix proteins.

Cluster analysis revealed that the mitochondrial matrix proteomes of the TA muscle tissue and HEK293T cells^10^ formed the most distinct clusters (Fig. 2a). The biotinylated protein patterns also displayed differences between the muscle tissues of MAX-Tg mice and cultured HEK293T cells (Extended Data Fig. 2c). In the TA muscle, the most abundant proteins were associated with energy production functions, including OXPHOS complex proteins and enzymes related to pyruvate regulation and the tricarboxylic acid cycle (Fig. 2b,c and Supplementary Data 2). Mitochondrial RNA processing proteins such as PTCD2 and PTCD3 were also highly expressed in the muscle. These proteins also play a role in the mitochondrial respiratory chain, reflecting the reliance of the muscle tissue on energy production via respiration^28,29^.

By contrast, data for HEK293T cells showed strong enrichment of mitochondrial chaperone proteins and transcription-related proteins (Fig. 2b,c and Supplementary Data 2). Several mitochondrial proteins involved in one-carbon metabolism and glutamate metabolism, which play important roles in cancer metabolism^30,31^, were also notably enriched in HEK293T cells. These findings underscore the distinct composition of mitochondrial matrix proteomes between mouse muscle tissues and the immortalized HEK293T cell line.

### Distinct matrix proteomes in multiple muscle tissues

Muscle fibers in different muscle groups exhibit distinct compositions, which can potentially influence their proteomes^32^. Overlapping analysis of the MTS-APEX2-labeled proteomes among the heart, TA and soleus muscles was performed (Fig. 2d and Supplementary Data 3). Several tissue-specific mitochondrial matrix proteins emerged, while 133 proteins were commonly identified across the three muscle tissues (among the total 250 proteins).

Proteins related to the beta oxidation pathway were more highly expressed in the heart than in the TA muscle (Extended Data Fig. 2d and Supplementary Fig. 1), aligning with the heart’s capacity to produce energy from fatty acids^33^. The heart also showed higher expression of mitochondrial quality control proteins, while the TA muscle showed higher expression of ubiquinone biosynthesis-related and ATP synthase complex proteins (Extended Data Fig. 2d and Supplementary Fig. 1). The expression levels of isotype proteins of isocitrate dehydrogenase (IDH) differed between the heart and muscle tissue; specifically, IDH3A, IDH3B and IDH3G, using NADH as a substrate^34^, were abundant in the TA muscle, whereas IDH2 (which uses NADPH) was more abundant in the heart (Supplementary Fig. 1).

The soleus skeletal muscle demonstrated high expression of ATP synthase complex and OXPHOS complex subunits compared to heart. (Extended Data Fig. 2e). The soleus muscle showed higher expression levels of mitochondrial proteins using NADPH/NADP in comparison to the TA muscle. Additionally, the soleus muscle showed higher expression levels of 5-demethoxyubiquinone hydroxylase (COQ7), which is vital for CoQ biosynthesis, while the TA muscle exhibited higher expression of complex III, mitochondrial ribosome subunits and pyruvate-related proteins (Extended Data Fig. 2f). These data highlight the unique mitochondrial matrix proteome composition of each muscle tissue.

### Further validation of the muscle-specific matrix proteome

**Extended Data Fig. 3:**
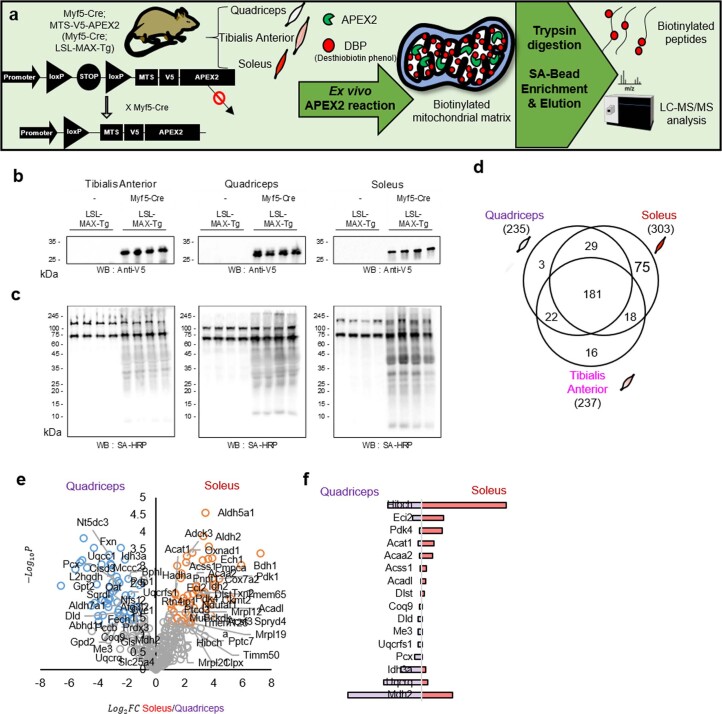
Muscle cell-type specific mitochondrial proteome mapping using ‘floxed’ LSL-MAX-Tg results (Myf5-Cre; LSL-MAX-Tg). (**a**) Scheme for muscle cell-specific mitochondrial matrix proteome mapping using LoxP-Stop-LoxP-MAX-Tg (LSL-MAX-Tg) mouse crossed with Myf5-Cre driver mouse. (**b**) Western blotting of MTS-V5-APEX2 (expected processed molecular weight: 28 kDa). Representative images from three independent experiments. (**c**) Streptavidin (SA)-HRP western blotting of biotinylated proteins. Representative images from three independent experiments are shown. (**d**) Venn diagram of identified mitochondrial matrix proteins from the tibialis anterior, quadriceps, and soleus tissues from Myf5-Cre; LSL-MAX-Tg mice. (see Supplementary Data 4 for detailed information) (e) Volcano plot of the DBP-labeled proteome labeled by MTS-APEX2 in quadriceps (left) vs. soleus (right) from Myf5-Cre; LSL-MAX-Tg mice. Analysis of statistically significant fold change revealed significantly different proteins between the quadriceps proteome and soleus proteome of Myf5-Cre; LSL-MAX-Tg mice. (f) Normalized mass intensities of asymmetrically DBP-labeled mitochondrial matrix proteins by MTS-APEX2 in the quadriceps and soleus muscle of Myf5-Cre; LSL-MAX-Tg mice. Source data

To address the need for profiling ‘cell type’-specific mitochondrial matrix proteomes, we generated a conditional MAX-Tg mouse model (LoxP-Stop-LoxP-MAX or LSL-MAX-Tg, Extended Data Fig. 3a). We crossed an LSL-MAX-Tg mouse with a Myf5-Cre driver mouse to obtain a muscle-fiber-specific proteome and confirmed the expression of MTS-V5-APEX2 and its biotinylation activity in three distinct skeletal muscle tissues (Extended Data Fig. 3a–c). We obtained Myf5-specific mitochondrial matrix protein information from these skeletal muscles by following the same protocol as that used with MAX-Tg mice (Extended Data Fig. 3d and Supplementary Data 4).

We compared the mitochondrial matrix proteomes between the quadriceps and soleus muscles, known for their highly diverse fiber type composition^32,35^. In the quadriceps, proteins related to NAD/NADH conversion, mitochondrial ATP synthesis and pyruvate metabolism were highly enriched. Conversely, the soleus muscle fibers exhibited high expression of proteins related to fatty acid beta oxidation, branched-chain amino acid catabolic process, and acetyl-CoA metabolism (Extended Data Fig. 3e,f). For the TA muscle, substantial overlap was observed between the APEX-labeled mitochondrial proteome results of the two genetically different Tg mouse models (Fig. 2e and Supplementary Data 5).

**Extended Data Fig. 4:**
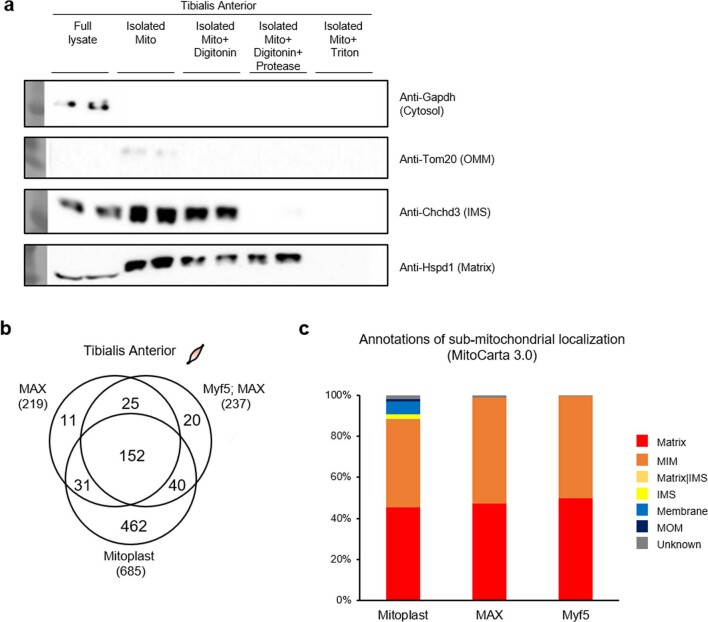
Comparison of sub-mitochondrial protein population in the mitoplast sample and APEX-labeled proteome of MAX-Tg mice (whole body expressed MAX-Tg mice and Myf5-Cre; LSL-MAX-Tg mice). (**a**) Validation of mitoplast with western blot using antibodies for sub-mitochondrial marker proteins. Representative images from three independent experiments. (**b**) Comparison of identified proteins in the mitoplast sample from TA muscle (WT mice), APEX-labeled proteins in TA muscle of MAX-Tg sample and APEX-labeled proteins in TA muscle of Myf5-Cre; MAX-Tg mice (**c**) Sub-mitochondrial protein population of mitochondria-annotated proteins in the mitoplast, APEX-labeled proteins of MAX-Tg (MAX), APEX-labeled proteins of Myf5-Cre; LSL-MAX-Tg (Myf5-MAX) in TA muscle samples. Sub-mitochondrial protein annotations were generated using Mitocarta3.0 (see Supplementary Data 5 for detailed information). Source data

We further investigated the protein overlap between the mitoplast proteome from the TA muscle tissue prepared using an established fractionation and proteolysis method^36–38^ and the MTS-APEX2-labeled mitochondrial protein data from the MAX-Tg and Myf5-Cre;LSL-MAX-Tg mice (Extended Data Fig. 4a,b and Supplementary Data 5). Although the mitoplast proteome identified more mitochondrial proteins, it showed notable contamination with other submitochondrial proteins. By contrast, the MAX-Tg and Myf5-Cre;LSL-MAX-Tg results showed minimal presence of other submitochondrial proteins (Extended Data Fig. 4b,c). These results support the reliability of our MAX-Tg mouse approach for identifying tissue- or cell type-specific mitochondrial matrix proteomes.

### RTN4IP1 is a mitochondrial matrix protein

Among the identified proteins, RTN4IP1, also known as optic atrophy-10 (OPA10), was consistently detected in the soleus muscle of both MAX-Tg and Myf5-Cre;LSL-MAX-Tg mice (Supplementary Data 3 and 4). Previous reports suggested RTN4IP1 is an outer mitochondrial membrane protein based on conventional fractionation and proteinase K protection assays^39,40^. However, our data consistently identified RTN4IP1 as a matrix protein across multiple samples. Consistently, RTN4IP1 was previously observed in the mitochondrial matrix proteome of HEK293T cells through an indirect biotinylated protein profiling method^13^. According to human genetic studies, *RTN4IP1* is associated with optic neuropathy, muscle loss and global developmental delay, indicating mitochondrial dysfunction^39,40^. Nonetheless, the molecular understanding of RTN4IP1 has remained unclear. Thus, we aimed to clarify its submitochondrial localization and identify its function in the mitochondrial matrix.

**Fig. 3:**
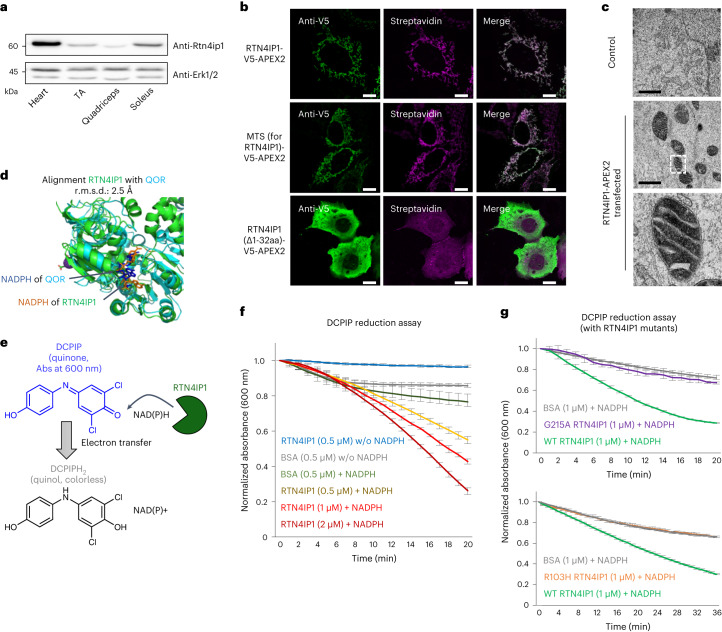
RTN4IP1 is localized at the mitochondrial matrix and displays NAD(P)H oxidoreductase activity. **a**, Western blotting of RTN4IP1 in the heart and three types of skeletal muscle. Anti-ERK1/2 was used as a loading control. Representative images from three independent experiments are shown. **b**, Confocal microscopy imaging of mitochondrial biotinylation by RTN4IP1-V5-APEX2 in HEK293T cells (anti-V5 imaging with the GFP channel, streptavidin imaging with the Cy5 channel). The N-terminal MTS (~R32) of RTN4IP1 was predicted by MitoFates. Scale bars, 10 µm. **c**, TEM images of RTN4IP1-APEX2-transfected HEK293T cells (right) and nontransfected HEK293T cells (left). Scale bars, 1 μm. Both samples were treated with DAB and H_2_O_2_, followed by OsO_4_ staining. Mitochondrial matrix DAB/OsO_4_ staining of RTN4IP1-APEX2 is highlighted in the magnified images of the white boxed region. **d**, Comparison of the crystal structure between RTN4IP1 and quinone NADPH oxidoreductase (QOR) (PDB ID 1QOR, blue). The molecular structure of cocrystalized NADPH is shown in the structure of RTN4IP1 and QOR. r.m.s.d., root mean-squared deviation. **e**, Scheme for the DCPIP assay of the QOR activity of RTN4IP1. **f**, Real-time monitoring results using blue-colored oxidized DCPIP as the quinone substrate (*n* = 3 independent experiments). DCPIP turns colorless when it accepts an electron from NADPH. BSA was used as a control. **g**, Real-time monitoring of oxidoreductase activity of mutated RTN4IP1 (G215A or R103H) with DCPIP and NADPH (*n* = 3 independent experiments). w/o, without. Source data

We first confirmed that RTN4IP1 expression was the highest in the heart, followed by the soleus muscle, with the TA muscle showing the lowest expression level (Fig. 3a). MitoFates^27^ prediction (0.900 value) and identification of biotinylated peptides in MTS-APEX2-labeled tissues strongly indicated its matrix localization. Immunofluorescence imaging of RTN4IP1-V5-APEX2 showed a mitochondrial expression pattern for both V5 and biotinylated proteins (Fig. 3b, top panel). We next tested the mitochondrial matrix-targeting capability of the MTS of RTN4IP1, which includes the N-terminal 32 amino acids. We expressed an APEX2 construct fused to this expected MTS. Confocal microscopy clearly demonstrated a mitochondrial distribution pattern for both V5 and biotinylated proteins (Fig. 3b, middle panel). We also generated an MTS-deleted RTN4IP1-APEX2 construct (RTN4IP1(Δ1-32aa)-V5-APEX2), which showed a clear cytosolic distribution in both anti-V5 and streptavidin staining (Fig. 3b, bottom panel). Collectively, these data strongly support the matrix localization of RTN41P1 rather than the outer mitochondrial membrane.

Last, APEX-electron microscopy^25^ confirmed the submitochondrial localization of APEX-tagged proteins. In RTN4IP1-APEX2-expressing cells, APEX-mediated DAB/OsO_4_ staining (darker regions) showed a staining pattern consistent with mitochondrial matrix localization (Fig. 3c). Taken together, these results confirmed that RTN4IP1 is a mitochondrial matrix protein with a strong mitochondrial matrix-targeting sequence.

### RTN4IP1 is a mitochondrial NAD(P)H oxidoreductase

**Extended Data Fig. 5:**
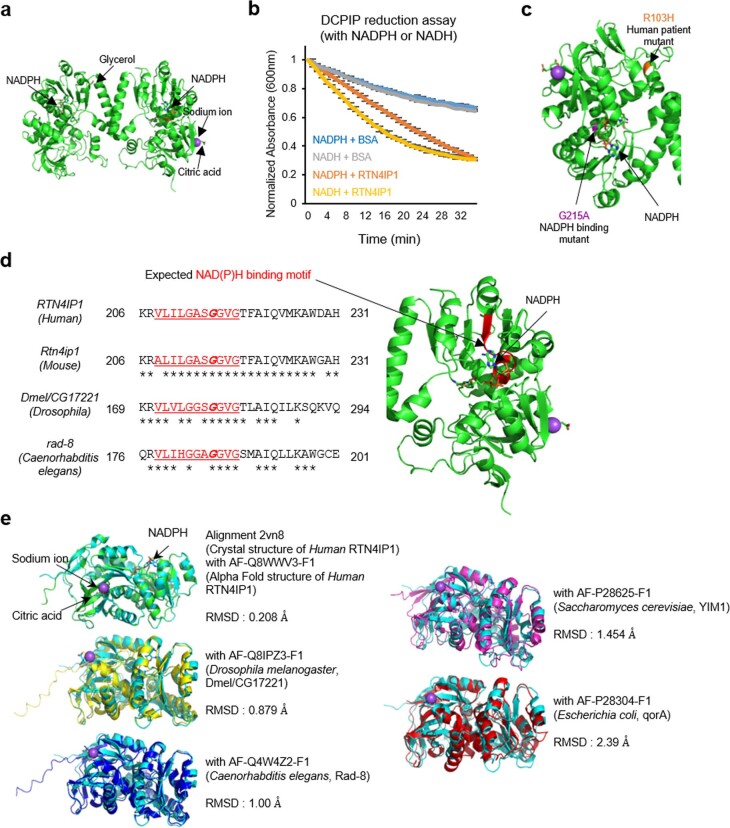
Structural similarity of human RTN4IP1 and RTN4IP1 orthologs in other organisms. (**a**) Crystal structure of RTN4IP1 (dimer form) from Protein Data Bank (PDB ID: 2VN8). (**b**) Real-time monitoring of the QOR activity of RTN4IP1 with DCPIP and NADPH or NADH (n = 3 independent experiments). (**c**) Position of mutated amino acid residues (R103H, G215A) in the crystal structure of RTN4IP1. (**d**) Sequence homology of G125 site at the NAD(P) binding region of RTN4IP1 in other organisms. (**e**) Structural similarity between the crystal structure of human RTN4IP1 (Uniprot ID: Q8WWV3, PDB ID: 2VN8) and the AlphaFold-predicted structure of human RTN4IP1 (AF-Q8WWV3-F1, https://alphafold.ebi.ac.uk/). Structural similarity between human RTN4IP1 crystal structure (PDB ID: 2VN8) and AlphaFold-predicted structures of RTN4IP1 orthologs in other organisms: CG17221 (Drosophila, AF-Q8IPZ3-F1), Rad-8 (C. elegans, AF-P28625-F1), Yim1p (Yeast, AF-P28625-F1), and qorA(E. coli, AF-P28304-F1) from AlphaFold database.

RTN41P1 is an NADPH-binding protein according to the Protein Data Bank (PDB) (2VN8) (Extended Data Fig. 5a). Considering that NADPH-binding proteins are rare in the mitochondrial matrix and are typically related to biosynthetic pathways or antioxidant control, we hypothesized that RTN4IP1 exerts enzymatic activity by using NADPH as a cofactor. Supporting this notion, structural homology analysis indicated that the reported structure of RTN4IP1 showed structural similarity to *Escherichia coli* quinone NADPH oxidoreductase (PDB ID 1QOR)^41^ in the NADPH-binding quinone oxidoreductase domain^42–44^ (Fig. 3d). This suggests that RTN41P1 has possible quinone oxidoreductase activity.

To assess the NADPH oxidoreductase activity of RTN4IP1 with a quinone substrate, we purified recombinant RTN4IP1 protein and measured its catalytic activity using the oxidation and reduction indicator 2,6-dichlorophenolindophenol (DCPIP)^45–47^ (Fig. 3e). As depicted in Fig. 3f, a rapid decrease in the absorption at 600 nm was observed on adding RTN4IP1 and NADPH. We also confirmed that RTN4IP1 could use NADH as a cofactor using the same DCPIP assay system (Extended Data Fig. 5b). These results indicate that RTN4IP1 plays a catalytic role in transferring electrons from NAD(P)H to electron acceptors such as quinone molecules in the mitochondrial matrix.

Next, we examined the perturbed oxidoreductase activity of RTN4IP1 mutants. We tested the G215A mutation located at the conserved NAD(P)H binding motif region (208-VLILGAS*G*GVG-218), which is predicted to bind the pyrophosphate group of NAD(P)H (Extended Data Fig. 5c,d)^48^. As expected, the G215A-RTN4IP1 mutant protein showed abolished oxidoreductase activity (Fig. 3g, top panel). We also tested a patient-derived RTN4IP1 mutation (R103H, c.308G>A) (Extended Data Fig. 5c), which is linked to optic neuropathy disease^39^. In our DCPIP assay, this R103H-RTN4IP1 mutant protein also showed negligible oxidoreductase activity compared to the WT RTN4IP1 protein (Fig. 3g, bottom panel), which indicates that this mutation can perturb the enzymatic activity of RTN4IP1.

### RTN4IP1 is required for CoQ biosynthesis

To further characterize the detailed molecular function of RTN4IP1, we conducted the interactome profiling of RTN41P1 in the mitochondrial matrix using TurboID^49^. Proteins biotinylated by RTN4IP1-TurboID were profiled via definitive mass spectrometric identification of biotinylated peptides with the biotinylated lysine residues (K + 226 Da)^50^. We used MTS-TurboID as a control sample, which is uniformly distributed throughout the mitochondrial matrix.

**Fig. 4:**
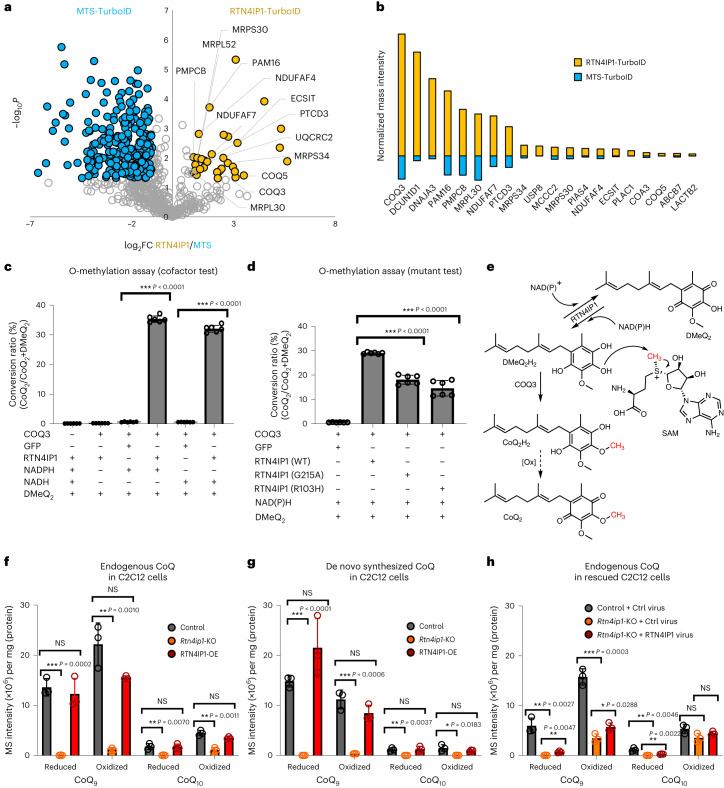
RTN4IP1 is required for CoQ synthesis. **a**, Volcano plot of MTS-TurboID (left) versus RTN4IP1-TurboID (right) biotinylated proteins (Supplementary Data 6). Statistical significance against FC revealed significantly different proteins between MTS-TurboID-labeled and RTN4IP1-TurboID-labeled samples. A total of 32 proteins were significantly biotinylated by RTN4IP1-TurboID (RTN4IP1 interactome) (*P* < 0.05, FC > 2). The proteins clustered by each function are highlighted. See Extended Data Fig. 6b for details. **b**, Normalized mass signal intensities of the top 20 biotinylated proteins labeled by RTN4IP1-TurboID among the 32 significantly biotinylated proteins. **c**,**d**, LC–PRM analysis of RTN4IP1-assisted O-methyltransferase activity of COQ3 using DMeQ_2_ as a substrate. All samples were incubated with *S*-adenosyl-methionine (SAM). The measured conversion ratio (that is (CoQ_2_)/(DMeQ_2_ and CoQ_2_)) is shown (*n* = 6 independent experiments): cofactor test (**c**) and mutant test (**d**). **e**, Proposed scheme for the O-methylation conversion of DMeQ_2_ to CoQ_2_ with COQ3/RTN4IP1. **f**–**h**, Histograms of LC–PRM measurement results for endogenous CoQs (**f**) and de novo-synthesized heavy CoQs (**g**) from heavy 4-HB (^13^C_6_) treatment in various C2C12 cells: *Rtn4ip1*-knockout (KO) cells (**f**), RTN4IP1-OE C2C12 cells (**g**) and *Rtn4ip1*-knockout (KO) and RTN4IP1-rescued C2C12 cells (**h**) (*n* = 3 biological replicates). The *y* axis is the normalized mass intensity unit per the sample’s protein mass; representative samples are shown. All precursor ions were cationized and detected as a form of ammonium adduct (NH_4_^+^) of the respective CoQ_9_ and CoQ_9_H_2_ molecules. Mean values are shown with error bars representing the standard deviation. Statistical significance was determined using a two-tailed Student’s *t*-test: *P < 0.05, **P < 0.01, ***P < 0.001; NS, not significant. Source data can be found in the Source Data file. Ctrl, Control. Source data

**Extended Data Fig. 6:**
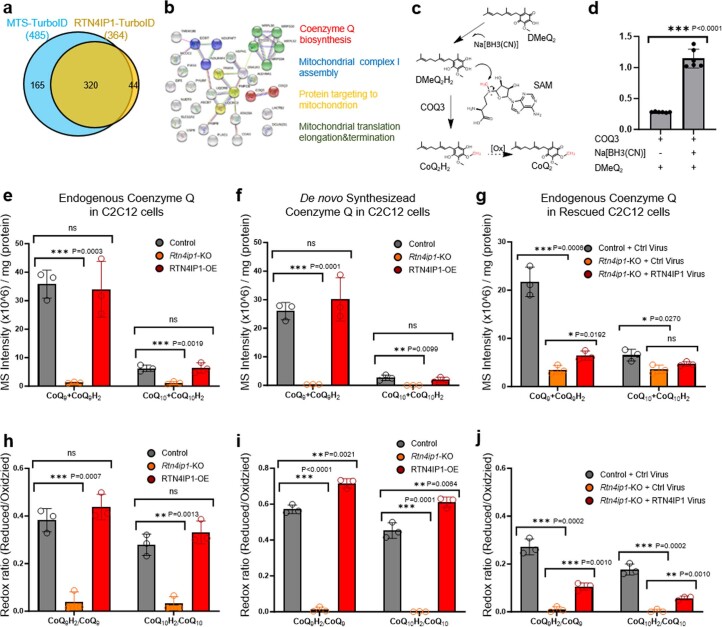
RTN4IP1 is required for CoQ synthesis. (**a**) Number of identified biotinylated proteins by MTS-TurboID or RTN4IP1-TurboID in HEK293T cells via Spot-ID workflow. See Supplementary Data 6 for detailed information. (**b**) Functional clustering with the RTN4IP1 interactome using STRING analysis. Molecular interaction network of 32 significant proteins biotinylated by RTN4IP1-TurboID (RTN4IP1 interactome) were analyzed by STRING analysis (https://string-db.org/). The clustered molecular functions are marked with different colors: red, coenzyme Q biosynthesis; blue, mitochondrial complex I assembly; green, mitochondrial translation elongation and termination; yellow, protein targeting the mitochondrion. (**c**) Proposed scheme for the O-methylation conversion of DMeQ2 to CoQ2 with COQ3 and Na[BH3(CN)]. (**d**) LC-PRM analysis of O-methyltransferase activity of COQ3 using DMeQ2 as a substrate. All samples were incubated with S-adenosyl-methionine (SAM). Measured conversion ratio (that is [CoQ2] / [DMeQ2 and CoQ2]) is shown. (n = 3 independent experiments) (**e**, **f**, **g**) Histograms of LC-PRM assay results of endogenous CoQ9 + CoQ9H2 and CoQ10 + CoQ10H2 (**e**, **g**) and de novo synthesized heavy CoQ9 + CoQ9H2 and CoQ10 + CoQ10H2 from heavy 4-HB (13C6) (**f**) in Rtn4ip1-knockout (KO), control, RTN4IP1-OE, RTN4IP1-rescued C2C12 cells. (n = 3 biological replicates) Y-axis is a normalized mass intensity unit per sample’s mass of protein. (**h**, **i**, **j**) Histogram of LC-PRM assay results representing CoQ9H2 %, CoQ10H2 % in Rtn4ip1-knockout (KO), control, RTN4IP1-OE, RTN4IP1-rescued C2C12 cells. Y-axis of histogram indicates the measured redox state ratio (that is [CoQ9H2]/[CoQ9 and CoQ9H2]). (n = 3 biological replicates) Mean values are shown with error bars representing the standard deviation. Statistical significance was determined using a two-tailed Student’s t-test: *p < 0.05, **p < 0.01, ***p < 0.001. Source data can be found in the Source Data file. Source data

A total of 639 and 438 proteins were identified by MTS-TurboID and RTN4IP1-TurboID, respectively. Among these, 399 proteins were common between the two experiments (Extended Data Fig. 6a and Supplementary Data 6). Through quantitative comparative analysis based on normalized mass intensity, we obtained 32 interactome candidate proteins that were consistently labeled by RTN4IP1-TurboID (*P* < 0.05, FC > 2; Fig. 4a and Extended Data Fig. 6b). Two proteins (COQ3, COQ5) are involved in CoQ biosynthetic processes; COQ3 was the most strongly biotinylated protein by RTN4IP1-TurboID, suggesting a potential involvement of RTN4IP1 in the CoQ production pathway within the mitochondria (Fig. 4b).

From this interactome analysis result, we hypothesized that RTN4IP1 regulates the function of COQ3, an O-methyltransferase that participates in the O-methylation steps of intermediates in CoQ biosynthesis. However, the regulatory mechanism underlying COQ3 has not been fully explained^51,52^. To determine whether the O-methyltransferase activity of COQ3 depends on RTN4IP1, we performed targeted mass spectrometric analysis using a high-resolution Orbitrap system in parallel reaction monitoring (PRM) mode^53^ to measure the conversion levels of demethylated CoQ_2_ (DMeQ_2_)^51^ to CoQ_2_ by COQ3. (Fig. 4c, Supplementary Fig. 2a,b and Supplementary Fig. 3a).

As a result, we observed that COQ3 generated a higher amount of CoQ_2_ (32–35% conversion) in the presence of RTN4IP1 compared to the control experiment (0.6–0.7% conversion). We also found that the O-methylation of DMeQ_2_ was not carried out by RTN4IP1 alone, supporting that RTN4IP1 assists with the O-methylation reaction of COQ3 as an interacting partner. Additionally, RTN4IP1 could not accelerate the O-methylation reaction of COQ3 in the absence of NADPH or NADH. We further confirmed that the COQ3 O-methylation activity of the two RTN4IP1 mutants (G215F, R103H) mutants was significantly attenuated compared to that of the WT RTN4IP1 protein (Fig. 4d). Furthermore, when we conducted the COQ3 O-methylation reaction using DMeQ_2_ with a reducing agent (sodium cyanoborohydride), there was an approximately fourfold increase in the level of O-methylation. However, this increase was not as high as when RTN4IP1 and NAD(P)H were involved (Extended Data Fig. 6c,d). These findings indicate the crucial role of RTN4IP1 in generating a reduced hydroquinone intermediate, which is subsequently used for the O-methylation reaction of COQ3 (Fig. 4e).

We expanded the mass spectrometric metabolite analysis to other CoQ molecules using three C2C12 mouse myoblast cell line samples: control, *Rtn4ip1*-knockout (KO) and RTN4IP1-overexpression (OE) cells. LC and PRM parameters for targeted MS analysis of CoQs were optimized using commercially available standard CoQ molecules (CoQ_2_, CoQ_9_ and CoQ_10_) and in house-synthesized CoQ derivatives (DMeQ_2_) (Supplementary Figs. 4–8). We identified CoQs and measured CoQ levels in the three cell lines, showing a marked reduction of CoQ_9_, CoQ_9_H_2_, CoQ_10_ and CoQ_10_H_2_ levels in *Rtn4ip1*-KO cells (Fig. 4f, Extended Data Fig. 6e,h and Supplementary Fig. 3b). Additionally, the ratio of reduced CoQ was decreased in *Rtn4ip1*-KO cells compared to that of control cells. These results were consistent with results from the LC–parallel reaction monitoring (LC–PRM) assay to quantify the levels of de novo synthesis of CoQs using a heavy version of 4-hydroxybenzoic acid (4-HB), a precursor molecule in CoQ synthesis^54,55^. As depicted in Fig. 4g (and Extended Data Fig. 6f,i and Supplementary Fig. 3b), de novo-synthesized heavy CoQ_9_ and heavy CoQ_10_ levels were significantly reduced in the *Rtn4ip1*-KO cells.

The levels of all types of CoQ, both endogenous and de novo-synthesized, did not increase significantly in RTN4IP1-OE C2C12 cells. This suggests that the endogenous expression level of RTN4IP1 is not a rate-limiting factor for CoQ biosynthesis (Fig. 4f,g). However, measurements with our de novo-synthesized CoQ revealed a significantly higher proportion of reduced CoQ in RTN4IP1-OE cells compared to that of control cells (Extended Data Fig. 6i). We also measured the CoQ levels in cells where RTN4IP1 was rescued from *Rtn4ip1*-KO cells (Fig. 4h, Extended Data Fig. 6g,j and Supplementary Fig. 3b). The amount of CoQ_9_ in the rescue cells increased; however, the CoQ quantity in the rescued cells was not fully restored to the levels in control cells, indicating potential irreversible mitochondrial damage. Furthermore, we observed a significant increase (2.69-fold) in the CoQ_2_ level when RTN4IP1-OE cells were treated with exogenous DMeQ_2_. This result confirmed that RTN4IP1 is involved in the O-methylation step of the demethylated precursors in CoQ synthesis (Supplementary Fig. 3c), supporting that RTN4IP1 is a crucial factor for CoQ synthesis in the mitochondria.

### RTN4IP1 deficiency induces oxidative stress

CoQs play a crucial role in mediating electron transport between Complex I/II and Complex III of the OXPHOS complex while also serving as an antioxidant^56^. Notably, CoQ metabolites have demonstrated protective functions against oxidative stress^56^. In the mitochondrial matrix, CoQ possesses a quinone moiety and functions as a well-characterized antioxidant in its reduced quinol state^57^. Mitochondrial-synthesized CoQ effectively protect against excessive reactive oxygen species (ROS) generation across various subcellular membranes through the lipid transport mechanism^56^. Therefore, we hypothesized that RTN4IP1 might protect the mitochondria from ROS by supporting the generation of protective CoQ.

**Fig. 5:**
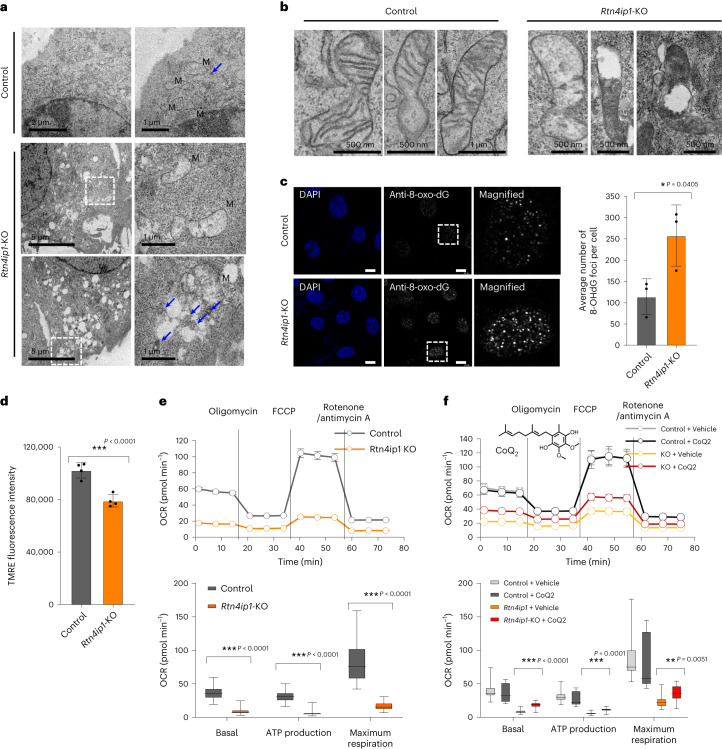
RTN4IP1/OPA10 regulates oxidative stress and mitochondrial respiration. **a**, TEM images of control (upper panel) and *Rtn4ip1*-knockout (KO) (lower panel) C2C12 cells. Mitochondrial structures are marked with ‘M’ and multilamella body structures are marked with blue arrows. **b**, Magnified TEM images of control (left panel) and *Rtn4ip1*-KO (right panel) C2C12 cells focusing on the mitochondria. **c**, Confocal microscopy images of control and *Rtn4ip1*-KO C2C12 cells. Scale bars, 10 µm. DNA regions oxidized by intracellular ROS were stained with an anti-8-oxo-dG monoclonal antibody and total DNA was stained with 4,6-diamidino-2-phenylindole (DAPI). The average number of 8-OHdG foci per cell was counted in images from control and *Rtn4ip1*-KO C2C12 cells (*n* = 3 biological replicates). **d**, Mitochondrial membrane potential measurement of control and *Rtn4ip1*-KO C2C12 cells by TMRE fluorescence. Mean fluorescence of the C2C12 cells in each sample was measured by a microplate reader (*n* = 4 biological replicates). **e**, Measurement of OCR, basal respiration, maximal respiration and ATP production in control and *Rtn4ip1*-KO C2C12 cells (*n* = 45 biological replicates). **f**, Rescued OCR of *Rtn4ip1*-KO cells by CoQ_2_. Basal respiration, ATP production and maximal respiration of CoQ_2_-treated *Rtn4ip1*-KO cells (*n* = 14 biological replicates). Mean values are shown with error bars representing the standard deviation. Statistical significance was determined using a two-tailed Student’s *t*-test: *P < 0.05, **P < 0.01, ***P < 0.001. Source data can be found in the Source Data file. Source data

**Extended Data Fig. 7:**
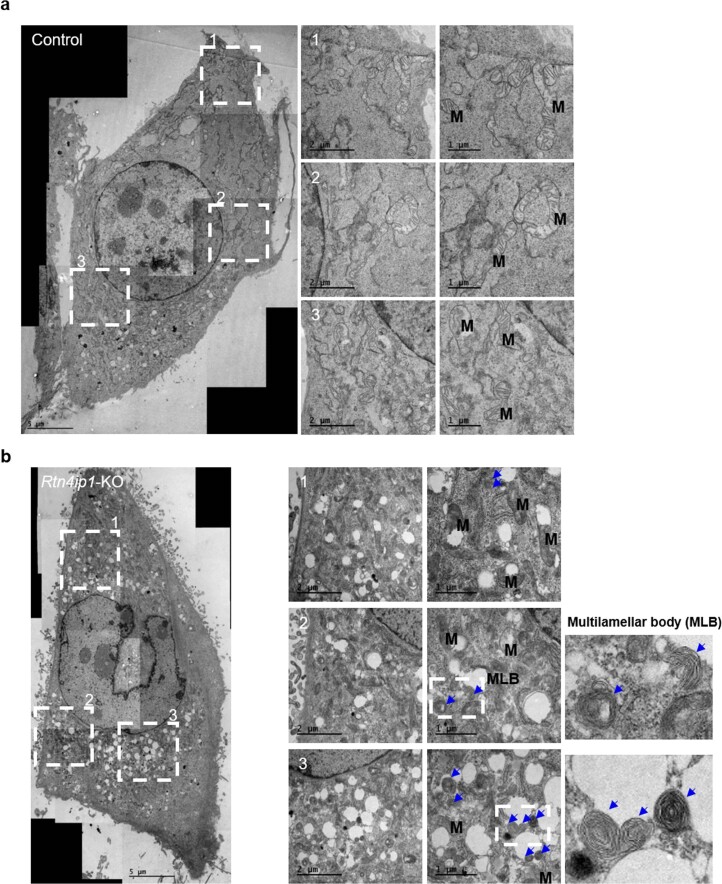
Additional TEM images of control (upper panel) and Rtn4ip1-KO (lower panel) C2C12 cells. Transmission electron microscopy (TEM) images of control (**a**) and *Rtn4ip1*-knockout (KO) (**b**) C2C12 cells. Mitochondrial structures are marked with ‘M’ and multilamella body (MLB) structures are marked with blue arrows.

To investigate this hypothesis, we conducted TEM imaging to evaluate subcellular ultrastructural changes arising from RTN4IP1 depletion. As shown in Fig. 5a, many vacuole-like structures were markedly increased in *Rtn4ip1*-KO C2C12 cells. Mitochondrial cristae of *Rtn4ip1*-KO cells were collapsed, the matrix exhibited reduced electron density and outer membrane rupture was observed in numerous mitochondria (Fig. 5b and Extended Data Fig. 7a,b), resembling structures seen under ferroptosis-inducing conditions^58–60^. Additionally, substantial multilamellar body structures, linked to the autophagic process to degrade damaged mitochondria^61^, were observed in *Rtn4ip1*-KO cells (Fig. 5a and Extended Data Fig. 7a,b).

Next, we assessed oxidative stress by measuring the oxidized nuclear DNA content in *Rtn4ip1*-KO cells using anti-8-oxo-dG monoclonal antibody (Fig. 5c). DNA staining using the antibody showed a higher count of foci in *Rtn4ip1*-KO cells (average 262 per cell) compared to that of control C2C12 cells (average 117 per cell), indicating increased foci in the nucleus (Fig. 5c). Collectively, these results indicate that RTN4IP1 deficiency leads to endogenous ROS accumulation and increased damage due to external oxidative stress, potentially due to a lowered level of the antioxidant CoQ.

### RTN4IP1 is essential for mitochondrial respiration

Given the essential role of CoQ in the electron transport chain, we further tested whether RTN4IP1 could modulate the OXPHOS activity of the mitochondria. We observed a substantial reduction in the tetramethylrhodamine, ethyl ester (TMRE) signal, as an indicator of mitochondrial membrane potential, in *Rtn4ip1*-KO cells compared to that of control C2C12 cells (Fig. 5d and Supplementary Fig. 9a,b).

**Extended Data Fig. 8:**
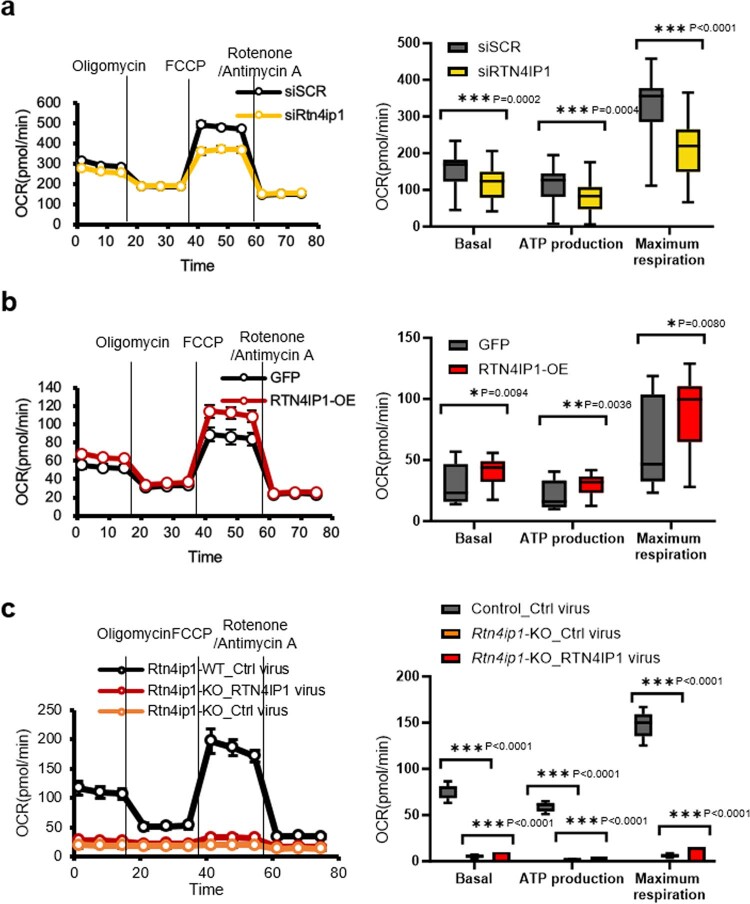
Oxygen consumption rate (OCR) and OXPHOS complex expression levels in RTN4IP1-KD C2C12 and RTN4IP1-overexpressed (RTN4IP1-OE) C2C12 cells. (**a**) Measurement of oxygen consumption rate (OCR), basal respiration, maximal respiration, and ATP production in control (siSCR) and Rtn4ip1 knockdown C2C12 cells (siRtn4ip1). (n = 46 biological replicates) (**b**) Oxygen consumption rate (OCR) and basal respiration, ATP production, and maximal respiration of RTN4IP1-overexpressed (RTN4IP1-OE) C2C12 cells. GFP-overexpressed cells were used as a control. (n = 30 biological replicates) (**c**) Oxygen consumption rate (OCR) and basal respiration, ATP production, and maximal respiration of RTN4IP1-rescued C2C12 cells. GFP-overexpressed cells were used as a control. (n = 30 biological replicates) Mean values are shown with error bars representing the standard deviation. Statistical significance was determined using a two-tailed Student’s t-test: *p < 0.05, **p < 0.01, ***p < 0.001. Source data can be found in the Source Data file. Source data

As a diminished membrane potential is directly linked to decreased OXPHOS activity in mitochondria due to oxidative damage to electron transport chain proteins, we next evaluated the oxygen consumption rate (OCR) and the expression of OXPHOS complex subunits. The overall OCR was lower in *Rtn4ip1*-knockdown (KD) C2C12 cells transfected with small interfering RNA (siRNA) compared to that of control cells (Extended Data Fig. 8a). However, there were no differences in the levels of OXPHOS complex proteins between the *Rtn4ip1*-KD and control cells (Supplementary Fig. 9c). In *Rtn4ip1*-KO cells, the decrease in OCR was even more pronounced, accompanied by reduced expression levels of OXPHOS complex proteins, particularly those within complexes I, III and V (Fig. 5e and Supplementary Fig. 9d).

Overexpression of RTN4IP1 increased the OCR of C2C12 cells (Extended Data Fig. 8b and Supplementary Fig. 9e). We also examined OCR in *Rtn4ip1*-KO cells expressing RTN4IP1; although there was a slight increase, the OCR did not fully recover to the level of control cells (Extended Data Fig. 8c and Supplementary Fig. 9f). This result indicates that RTN4IP1-deficient mitochondria may suffer substantial damage, which cannot be easily recovered by its subsequent expression. Future investigations should consider the potential of permanent mitochondrial damage due to CoQ deficiency and the broader mitochondrial roles of RTN4IP1, independent of CoQ synthesis. Collectively, these results are in good agreement with the elevated CoQ levels in the same cell lines (Fig. 4f–h). Taken together, these data indicate that RTN4IP1 potentially affects OXPHOS activity through CoQ biosynthesis regulation.

### Short-tail CoQ rescue deficiency of RTN4IP1 in flies

Next, we tested whether the CoQ-deficient phenotype of *Rtn4ip1*-KO C2C12 cells could be ameliorated through CoQ analog supplementation. CoQ_2_ with a short isoprene tail exhibits enhanced membrane permeability compared to that of longer-tail CoQ molecules, making it advantageous for mitochondrial delivery^62^. We found that treatment with CoQ_2_ contributed to the partial rescue of reduced respiratory defects of *Rtn4ip1*-KO cells (Fig. 5f).

**Fig. 6:**
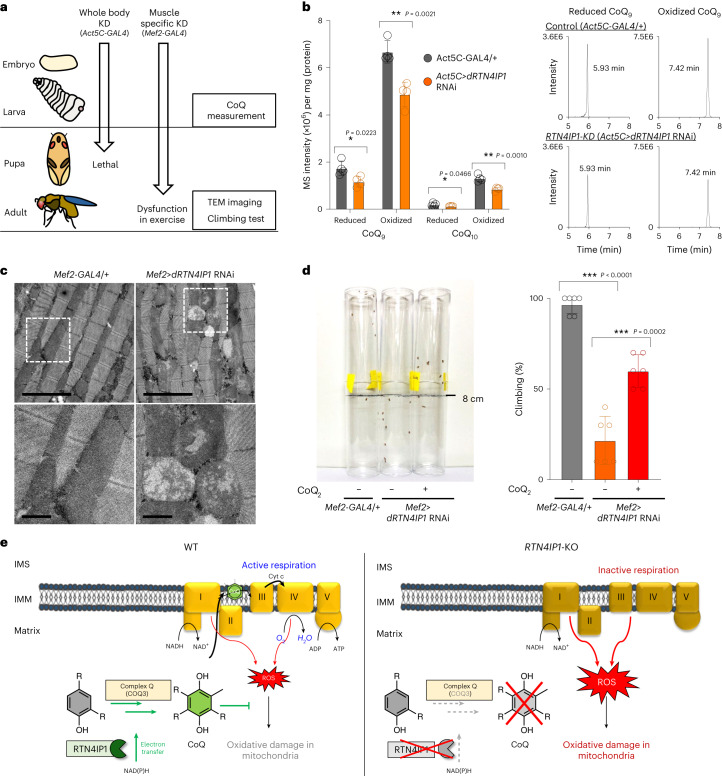
*dRTN4IP1*, a *Drosophila* ortholog of *RTN4IP1*, is required for CoQ biogenesis and mitochondrial function in *Drosophila*. **a**, Scheme of experiments using *Act-GAL4* (whole body) and *Mef2-GAL4* (muscle) drivers in *dRTN4IP1*-knockdown (KD) flies and their phenotypes. **b**, Histograms of LC–PRM assay results and representative LC–PRM chromatograms of endogenous CoQ_9_, CoQ_9_H_2_, CoQ_10_ and CoQ_10_H_2_, which were all detected as a form of ammonium adduct (NH_4_^+^), in control and whole-body Dmel/CG17221-KD fruit fly larvae (*n* = 4 biological replicates). **c**, TEM images of the mitochondrial morphology of indirect flight muscle in muscle-specific *dRTN4IP1*-KD flies. Scale bars, 5 µm (upper) and 1 µm (lower). **d**, Climbing assay for muscle-specific *dRTN4IP1*-KD (left), control (middle) and CoQ_2_-treated flies of muscle-specific dRTN4IP-KD (right). In the negative geotaxis assay, flies climbing over 8 cm were counted over 10 s. For CoQ_2_ treatment, CoQ_2_-containing foods (50 μg g^−1^) were treated to 3–5-day male flies for 24 h (*n* = 6 biological replicates). **e**, Proposed model of the RTN4IP1–CoQ axis and its function in the mitochondria. Mean values are shown with error bars representing the standard deviation. Statistical significance was determined using a two-tailed Student’s *t*-test: *P < 0.05, **P < 0.01, ***P < 0.001. Source data can be found in the Source Data file. Source data

**Extended Data Fig. 9:**
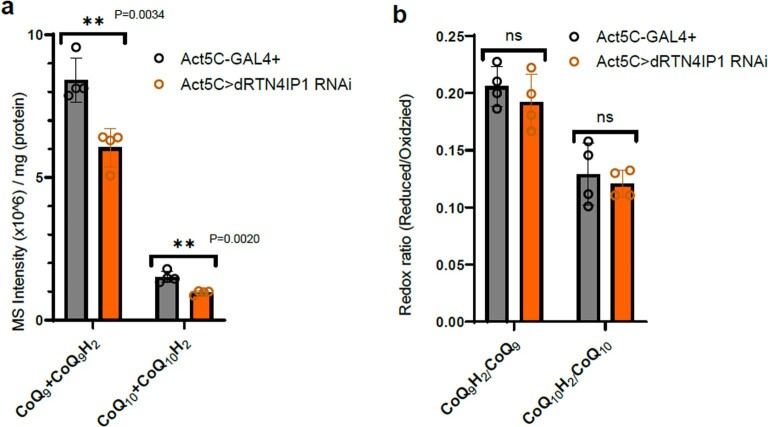
Coenzyme Q level in whole body Dmel/CG17221-KD fruit fly larvae and control. (**a**) Histograms of LC-PRM assay results of endogenous CoQ9 + CoQ9H2 and CoQ10 + CoQ10H2 in control and whole body Dmel/CG17221-KD fruit fly larvae. (n = 4 biological replicates) Y-axis is a normalized mass intensity unit per sample’s mass of protein. (**b**) Histogram of LC-PRM assay results representing CoQ9H2%, CoQ10H2% in control and whole body Dmel/CG17221-KD fruit fly larvae. Y-axis of the histogram indicates the measured redox state ratio (that is, [CoQ9H2]/[CoQ9 and CoQ9H2]). (n = 4 biological replicates) Mean values are shown with error bars representing the standard deviation. Statistical significance was determined using a two-tailed Student’s t-test: *p < 0.05, **p < 0.01, ***p < 0.001. Source data can be found in the Source Data file. Source data

Notably, whole-body *Rtn4ip1*-KO in mice has been reported as lethal according to the International Mouse Phenotype Consortium dataset, pointing to the essential physiological role for RTN4IP1. Hence, we used a *Drosophila* model, which carries a conserved ortholog of *RTN4IP1* (*dRTN4IP1*, *Dmel/CG17221*) to study the in vivo RTN4IP1 function (Extended Data Fig. 5e). Ubiquitous expression of *dRTN4IP1* RNAi with *Act5C-GAL4* (*Act5C*>*dRTN4IP1* RNAi) resulted in a pupal lethal phenotype (Fig. 6a). LC–PRM analysis showed a decreased number of CoQ species in *dRTN4IP1*-KD flies during third-instar larvae stage compared to that of the control (*Act5C-GAL4/+*) (Fig. 6b and Extended Data Fig. 9a,b). These results indicate that dRTN4IP1 is also required for CoQ synthesis in *Drosophila*.

Finally, we tested whether CoQ treatment could rescue respiratory defects in the fly in vivo model. Muscle-specific knockdown of *RTN4IP1* with *Mef2-GAL4* (*Mef2*>*dRTN4IP1* RNAi) produced viable adults, but showed striking mitochondrial defects with severely perturbed cristae structures as assessed by TEM imaging of indirect flight muscles (Fig. 6a,c). These flies also displayed dramatically reduced locomotor activity in a climbing assay (Fig. 6d). The locomotor defect of *dRTN4IP1-*KD flies was significantly restored by supplementation of CoQ_2_ in the diet for 24 h (Fig. 6d and Supplementary Video 1). These results indicate that the loss of dRTN4IP1 in the fly muscle tissue directly leads to CoQ deficiency and motor defects, which can be rescued by CoQ analog treatment. Overall, our findings indicate the indispensable role of the RTN4IP1–CoQ axis in maintaining mitochondrial respiration and muscle function (Fig. 6e).

## Discussion

We established MAX-Tg mice, facilitating the profiling of tissue-specific matrix proteomes, and used MTS-APEX2 to efficiently label mitochondrial matrix proteins. The robust activity of APEX2 observed in vivo within muscle tissues is consistent with a recent report using APEX in the mouse brain for electron microscope imaging^63^.

RTN4IP1 was first proposed as a mitochondrial protein interacting with the endoplasmic reticulum membrane protein reticulon 4 (RTN4) (also known as neurite outgrowth inhibitor or NOGO) using a yeast two-hybrid assay^64^ and coimmunoprecipitation experiment. However, the detailed molecular mechanisms of RTN4IP1 underlying its role in OXPHOS activity at the mitochondrial matrix/inner mitochondrial membrane^39,40^ are yet to be investigated. In the present study, we revealed that RTN4IP1 is mainly expressed in the mitochondrial matrix of the heart and soleus muscle tissues and is involved in CoQ biosynthesis assisting the O-methyltransferase function of COQ3.

We identified that RTN4IP1 assists the O-methylation reaction of COQ3 in the presence of a reducing agent. These findings suggest that the temporary reduction of the DMeQ_2_ intermediate to the hydroquinone intermediate by RTN4IP1/NAD(P)H serves as a crucial substrate in the O-methylation reaction of COQ3. It is worth noting that the hydroxyl group of the reduced hydroquinone can function as a methyl acceptor in the O-methylation reaction of COQ3 with SAM^51^, and our results indicate that the reductase activity of RTN4IP1 can provide the reduced hydroquinone substrate for the sequential O-methylation reaction of COQ3. Additionally, RTN4IP1 and COQ3 have strong coexpression or codependency profiles (RCHS4 and Depmap database) (Supplementary Table 1). These profiles also support coupling of the enzymatic function of RTN4IP1 and COQ3 for CoQ synthesis. It is noteworthy that the C-methylation reaction of COQ5 also requires putative reductase activity^65^. Given that we identified COQ5 as part of the interactome of RTN4IP1-TurboID, it will be intriguing to further investigate whether RTN4IP1 can assist this reaction of COQ5.

Notably, *RTN4IP1* is highly conserved across species, including humans. The similarity of predicted three-dimensional structures of *RTN4IP1* orthologs by Alphafold^66^ (Extended Data Fig. 5e) indicates that these *RTN4IP1* orthologs in eukaryotic systems may exhibit similar NAD(P)H activity in CoQ biosynthesis. Here, we confirmed that the *RTN4IP1* ortholog in *Drosophila* is involved in CoQ synthesis for maintaining mitochondrial function. An ortholog of *RTN4IP1* in *Caenorhabditis elegans* (*Rad-8*) was also identified as a mitochondrial protein, with mutations of this gene causing increased sensitivity to oxidative stress and mitochondrial defects^67^. The yeast ortholog of *RTN4IP1*, *Yim1p*, is also identified as a mitochondrial NAD(P)H quinone oxidoreductase protein and its mutant showed reduced resistance to oxidative stress^68^. Further investigations are required to confirm the relationship between *RTN4IP1* orthologs and CoQ levels in these organisms.

Our results also offer insights in the treatment of conditions associated with genetic defects of *RTN4IP1*. The decreased mobility in the muscle-specific *dRTN4IP1*-KD fruit fly accurately reflects the reported muscle diseases in human patients carrying defects in *RTN4IP1* (refs. ^39,40,69–71^). Using the fruit fly model, we demonstrated that soluble CoQ supplements could alleviate locomotor defects in *dRTN4IP1-*KD flies. These results indicate that CoQ supplementation could be a potentially effective treatment for diseases in humans resulting from *RTN4IP1* mutations.

Moreover, our data indicate the presence of other intriguing muscle-specific mitochondrial proteins such as MYOM2 and PRDX1 (Supplementary Table 2 and Supplementary Data 5). These proteins show mitochondrial localization patterns in the Human Protein Atlas. Moreover, many of our muscle-specific mitochondrial proteins show high expression in muscle tissues and in the human tissue transcriptome database (GTEx) (Supplementary Table 3). This suggests that our findings from MAX-Tg mice might be relevant to tissue-specific expression in humans. We anticipate that these proteins may play a muscle-specific role in supporting mitochondrial function, which requires further investigation.

In conclusion, we successfully used the APEX2 technique to generate MAX-Tg mice, which can be used for obtaining tissue-specific mitochondrial matrix proteome data. Through these MAX-Tg mice, we unveiled new mitochondrial matrix proteins, particularly RTN4IP1, demonstrating its role as an NAD(P)H oxidoreductase that supports the O-methyltransferase activity of COQ3 in CoQ biosynthesis. This study demonstrates the potential use of MAX-Tg mice in identifying tissue-specific mitochondrial proteins and their metabolic implications in diverse disease models.

## Methods

### Matrix-V5-APEX2 (MAX-Tg) mouse model

MTS-V5-APEX2 Tg mice were generated, interbred and maintained in specific pathogen-free conditions at Macrogen (Seoul, Republic of Korea). All manipulations were conducted under Macrogen Institutional Animal Care and Use Committee approval. Briefly, MTS-V5-APEX2 DNA was comicroinjected into a single-cell embryo. Fourteen to 16 injected single-cell-stage embryos were surgically transplanted into the oviducts of pseudopregnant recipient ICR mice. After the F0 offspring were born, genotyping was performed by PCR. MAX-Tg mice were mated with WT mice and pups were born at the expected Mendelian ratio.

### LSL-Matrix-APEX2 Tg (LSL-MAX-Tg) mouse model

LSL-Matrix-APEX2 Tg (LSL-MAX-Tg) mice were generated, interbred and maintained in specific pathogen-free conditions at Cyagen Biosciences (Taicang City, China). The genomic RNA (CTCCAGTCTTTCTAGAAGATGGG) of the mouse *Rosa26* gene locus, the donor vector containing the ‘CAG promoter-loxP-3*polyA-loxP-Kozak-MTS-V5-APEX2-polyA’ cassette, and *Cas9* messenger RNA were coinjected into fertilized mouse eggs to generate targeted conditional knockin offspring. F0 founder animals were identified by PCR followed by sequence analysis, which were bred to WT mice to test germline transmission and F1 animal generation.

### Cell culture and transfection

HEK293T and C2C12 cells were obtained from the American Type Culture Collection (ATCC), which were used in more than 20 passages. HEK293T Flp-in T-rex cells were obtained from ThermoFisher Scientific (catalog no. R78007). The cell lines were frequently checked and tested for morphology and *Mycoplasma* contamination under a microscope but were not authenticated. All cell lines were cultured in high-glucose Dulbecco’s modified Eagle medium (DMEM) with 10% fetal bovine serum (FBS) at 37 °C in 5% CO_2_ (v/v). All cell lines were transiently transfected at 60–80% confluence using polyethyleneimine.

### Histology and immunofluorescence staining

Mice were anesthetized with isoflurane and the muscles were dissected. Isolated muscles were frozen in a beaker filled with a slurry of isopentane at −80 °C and stored in a deep freezer until further analysis. Frozen muscles were sectioned using a cryostat (Leica CM3050S) at 10 μm thickness. Cryosections were used for hematoxylin-eosin staining and immunofluorescence analysis. For immunofluorescence, the sections were fixed with ice-cold methanol for 10 min, permeabilized with 0.25% Triton X-100 for 15 min and blocked with 1% bovine serum albumin (BSA)/Tris-buffered saline with Tween (TBST) solution for 30 min at room temperature. The sections were incubated overnight at 4 °C with primary antibodies (anti-V5, anti-laminin, anti-TOM20, streptavidin-Alexa Fluor 647) diluted in 1% BSA/TBST solution. After washing with TBST solution, the sections were incubated at room temperature for 1 h with the secondary antibody diluted in 1% BSA/TBST solution. The stained sections were incubated with Hoechst 33342 solution for 5 min at room temperature and then mounted with antifaded fluorescence mounting medium. All images were acquired on a Zeiss LSM 880 confocal microscope.

### Immunofluorescence and confocal microscopy

To visualize the subcellular localization of transiently expressed RTN4IP1-V5-APEX2, HEK293T cells were plated on coverslips (thickness no. 1.5, radius 18 mm). The cells were fixed in 4% paraformaldehyde and permeabilized with cold methanol for 5 min at −20 °C, followed by washing with Dulbecco’s phosphate-buffered saline (DPBS) and blocking for 1 h with 2% BSA in DPBS at room temperature. Immunolabeling was conducted in blocking solution with appropriately diluted antibodies (anti-V5 and anti-TOM20) and Alexa Fluor-labeled secondary antibodies (anti-streptavidin-Alexa Fluor 647, mouse anti-Alexa Fluor 488 and rabbit anti-Alexa Fluor 568) with extensive washes. Immunofluorescence images were obtained and analyzed on an SP8 X Leica microscope with an objective lens (HC PL APO ×100/1.40 oil), white light laser (470–670 nm, 1 nm tunable laser) and Hybrid Detector (HyD), which was controlled with LAS X software.

### Immunofluorescence imaging of oxidized DNA

To visualize the 8-oxo guanosine, control or *Rtn4ip1*-KO C2C12 cells were plated on coverslips (thickness no. 1.5, radius 18 mm). The cells were fixed and permeabilized with cold methanol for 30 min at −20 °C, followed by washing with DPBS. To eliminate RNA, the cells were treated with RNase solution for 1 h at 37 °C. After washing three times with DPBS, nuclear DNA was denatured with 2 N HCl for 10 min. Following another set of three washes with DPBS, the cells underwent a 1 h blocking step at room temperature using a 2% BSA solution in DPBS. Immunolabeling was performed within this blocking solution using a suitably diluted anti-8-OhdG antibody and mouse anti-Alexa 568. The cells were then counterstained with 4,6-diamidino-2-phenylindole. Immunofluorescence images were obtained and analyzed on an SP8 X Leica microscope with an objective lens (HC PL APO ×100/1.40 oil), white light laser (470–670 nm, 1 nm tunable laser) and HyD detector, which was controlled with LAS X software. Foci stained with anti-8-oxo-dG were counted using ImageJ software.

### Measurement of membrane potential

TMRE fluorescence analysis was conducted using flow cytometry and a microplate reader. Control or *Rtn4ip1*-KO C2C12 cells were cultured in an incubator (37 °C, 5% CO_2_). As a control experiment, the cells were treated with 200 µM of mesoxalonitrile 4-trifluoromethoxyphenylhydrazone (FCCP) for 3 h. For flow cytometry, the cells were collected using trypsin treatment and then resuspended in 0.5 ml of DMEM supplemented with 5% FBS containing 200 nM of TMRE for 30 min at 37 °C. To avoid background fluorescence signals, gating was applied based on intensity levels observed in negative control samples (TMRE nontreated). Cellular fluorescence was measured using the Flow Activated Cell Sorter (FACS CantoII). Data were analyzed by BD FACSDiva Software. Fluorescence excitation at 520 nm and emission at 580 nm was evaluated on a black 96-well culture plate with a clear bottom using a microplate reader (Molecular Devices).

### OCR measurement

Real-time measurements of OCR were performed using the Seahorse Xfe96 Extracellular Flux Analyzer (Agilent) with Seahorse XF Cell Mito Stress Test Kit (catalog no. 103015-100). One day before the measurements, cells were plated at 1.2 × 10^4^ cells per well. One hour before the measurements, the cell culture medium was changed to prewarmed DMEM supplemented with 10 mM glucose, 1 mM pyruvate and 2 mM glutamine. To test mitochondrial stress, 1.5 µM oligomycin, 2 µM FCCP, 0.5 µM rotenone and 0.5 µM antimycin A were used according to the suggested protocol from the manufacturer. OCR was measured according to the manufacturer’s Cell Mito Stress Test protocol. For knockdown experiments, 4.0 × 10^4^ cells per ml were reverse-transfected with siRNA and seeded at 0.8 × 10^4^ cells per well 2 days before the measurements.

### Recombinant RTN4IP1 expression and purification

The DNA fragments encoding WT RTN4IP1 were amplified by PCR and cloned into a modified pET-21a vector. For protein production, the plasmids were transformed into *Escherichia coli* BL21 (DE3) cells. Protein expression was induced with 0.3 mM isopropyl-β-d-1-thiogalactopyranoside when the cells reached an absorbance of 0.6 at an optical density of 600 nm; culturing was continued at 18 °C for 18 h. The cells were collected using centrifugation at 5,000*g* for 10 min and resuspended in lysis buffer (25 mM sodium phosphate pH 7.8, 400 mM sodium chloride and 10 mM imidazole). After cell lysis by sonication, lysed cells were clarified using centrifugation for 30 min at 10,000*g*. The supernatant was applied onto an Ni^2+^-IMAC affinity column equilibrated with binding buffer consisting of 25 mM sodium phosphate (pH 7.8), 400 mM NaCl and 10 mM imidazole. The proteins were eluted with binding buffer supplemented with 400 mM imidazole. The proteins were purified using Amicon filters (Milipore) and eluted in buffer with PBS. The protein solution was concentrated to approximately 5 mg ml^−1^ and flash-frozen in liquid nitrogen for storage.

### RTN4IP1 oxidoreductase activity

Oxidoreductase activity of RTN4IP1 was determined by measuring the absorption of DCPIP (50 μM) spectrophotometrically at 600 nm using the SpectraMax i3x Multi-Mode Microplate Reader (Molecular Devices). Absorption was measured after adding purified RTN4IP1 (100 nM) protein or BSA (100 nM) to a reaction mixture containing 100 mM Tris-HCl, 0.01% Tween 20 and 200 μM NADPH (pH 7.4).

### Construction of the RTN4IP1-V5-TurboID cell line

Flp-In T-Rex 293 cells were maintained in DMEM supplemented with 10% FBS, 2 mM l-glutamine, 50 units ml^−1^ penicillin and 50 µg ml^−1^ streptomycin at 37 °C under 5% CO_2_. Cells were grown in a T25 flask. Stable cell lines were first generated by transfection with the pcDNA5 expression construct plasmid expressing RTN4IP1-V5-TurboID or Matrix-V5-TurboID. Cells were transfected at 60–80% confluence using 6 μl of polyethyleneimine transfection reagent and 2,000 ng plasmid per six-well cell culture plate. After 24 h, the cells were split into a 90 mm cell culture dish (SPL, 11090) with hygromycin (2 µg ml^−1^). Media containing hygromycin were changed every 3–4 days. After 2–3 weeks, 3–4 colonies were selected and transferred to a 24-well plate. The cells were continuously split into larger plates and a cell stock was prepared. After splitting the cells into a six-well plate, separate samples were prepared for expression detection. RTN4IP1-V5-TurboID or Matrix-V5-TurboID expression was induced by 5 ng m^−1^ doxycycline.

### Construction of the Rtn4ip1-KO C2C12 cell line

The CRISPR–Cas9 technique was used to generate knockout cell lines. Single-guide RNAs (sgRNAs) were designed using the CRISPR RGEN Tools website (http://www.rgenome.net); the sequence 5′-GGAAGCGGTCGAAAGATAAA-3′ was used as a non-target control, while 5′-TCTGCCATAAACAAGGTTGG-3′ was used to target exon 4 of the mouse *Rtn4ip1* gene. Each sgRNA was cloned into the lentiCRISPRv2 vector. Production and transduction of CRISPR lentivirus was performed and puromycin (2 µg ml^−1^) was used to select knockout cells.

### Construction of the RTN4IP1-overexpressed C2C12 cell line

A retrovirus was used to generate overexpression cell lines. MSCV PIG (Puro-IRES-GFP) was a gift from S. Lowe (Addgene catalog no. 18751). Vector and PCR-amplified human *RTN4IP1* complementary DNA were digested with XhoI and EcoRI for ligation. The cloned vector was transfected to Phoenix-AMPHO (ATCC, CRL-3213) using Lipofectamine 2000. Sixteen hours after plasmid transfection, the culture medium was changed to a fresh medium with 1 mM sodium butyrate (Sigma, B5887). Twenty-four hours after the medium change, the medium was collected, centrifuged at 400*g* for 5 min and filtered by a 0.45 μm PES syringe filter. The filtered medium was mixed with fresh medium at a 1:2 ratio with 6 μg ml^−1^ polybrene to infect C2C12 cells. Transduced cells were selected by puromycin.

### Mitoplast preparation from the skeletal muscle of mice

Dissected muscle tissues were minced and washed with Chappel–Perry buffer (50 ml of 1 M KCl, 25 ml of 0.5 M MOPS (pH 7.4), 25 ml of 0.1 M MgSO_4_-7H_2_O, the volume brought up to 500 ml with ddH_2_O and 3.03 g ATP; pH 7.4). Chappel–Perry buffer (10 ml g^−1^ of tissue) with dispase (1 mg g^−1^) was added and the samples were incubated on ice for 5 min with constant stirring, followed by the addition of trypsin (10 mg g^−1^) and an additional incubation of 15 min. The samples were then homogenized with a tight pestle three times. The reaction was stopped by adding Chappel–Perry-2 buffer (150 ml of Chappel–Perry buffer with 2.5 ml of 0.1 M EGTA and 0.3 g of BSA) (10 ml g^−1^ tissue). The protease was removed by centrifuging at 12,000*g* for 10 min and the pellet was resuspended in 10 ml g^−1^ tissue Chappel–Perry-2 buffer. The samples were further homogenized with the tight pestle thrice and centrifuged at 600*g* for 10 min. The supernatant was collected and 10 ml g^−1^ of tissue Chappel–Perry-2 buffer was added to the pellet. The pellet was homogenized with a loose pestle twice, followed by centrifugation at 600*g* for 10 min. The supernatant was collected, combined with the first supernatant, and filtered through a cheesecloth. The mitochondria were pelleted by centrifuging at 7,000*g* for 20 min. KME buffer (10 ml g^−1^ tissue; 100 mM KCl, 50 mM MOPS, 0.5 mM EGTA) was added to the pellet, followed by digitonin (1 mg ml^−1^). Trypsin and proteinase K were added (final concentration of 5 µg ml^−1^ each) and samples were incubated for 15 min at 4 °C. Proteases were inactivated by adding phenylmethylsulfonyl fluoride (1 mM final concentration) and incubating for 10 min at 4 °C. Finally, the samples were centrifuged at 7,000*g* for 20 min.

### APEX2-mediated in situ biotinylation reaction in live cells

All cell lines expressing APEX2 were incubated with 250 μM biotin phenol for 30 min, followed by treatment with 1 mM H_2_O_2_ and quenching with 1 M sodium azide, Trolox and sodium ascorbate. The cells were then lysed with RIPA buffer containing a 1× protease cocktail for immunoblotting or fixed in 4% paraformaldehyde for immunofluorescence.

### In situ biotinylation reaction in the muscle of MAX-Tg mice

The dissected tissues were placed in test tubes and incubated with DBP (500 μM) in PBS for 1 h. Subsequently, diluted H_2_O_2_ (20 mM) was added to each sample for a final concentration of 2 mM H_2_O_2_ and the tubes were gently agitated for 2 min. The reaction was then quenched by adding DPBS containing 10 mM Trolox, 20 mM sodium azide and 20 mM sodium ascorbate to the tubes. The labeled tissues were homogenized using a bead beater. Homogenized tissues were lysed with 1 ml of RIPA buffer containing 1× protease inhibitor cocktail. Each sample was immunoblotted with anti-V5 and streptavidin-horseradish peroxidase to detect the expression of the processed bait protein (MTS-V5-APEX2) and DBP-modified proteins by Matrix-APEX2, respectively. Line-scan analysis was carried out using ImageJ. After subtraction of the background intensity value, protein signals from the top to the bottom of the protein marker were placed on the *x* axis, while the signal intensity from the top to the bottom of each lane was placed on the *y* axis.

### Biotin labeling and tissue lysis

The dissected muscle tissues were placed in test tubes and incubated with DBP (500 μM) in PBS for 1 h. Diluted H_2_O_2_ (20 mM) was then added to each sample for a final concentration of 2 mM H_2_O_2_ and then the tubes were gently agitated for 2 min. The reaction was then quenched by adding DPBS containing 10 mM Trolox, 20 mM sodium azide and 20 mM sodium ascorbate to the tubes. The labeled tissues were homogenized using a bead beater. Homogenized tissues were lysed with 1 ml of lysis buffer (4% sodium dodecyl sulfate in 1× TBS containing 1× protease inhibitor cocktail). For clarifying, the lysates were ultrasonicated (Bioruptor) for 15 min; each step was performed on ice or at 4 °C in a cold room.

### Biotin labeling by RTN4IP1-V5-TurboID and cell lysis

For mass sampling, RTN4IP1-V5-TurboID and Matrix-V5-TurboID stable cells were grown in three T75 flasks to obtain triplicate samples. For transiently expressing constructs, cells at 70–80% confluence were treated with 5 ng ml^−1^ doxycycline. After 16 h, 50 μM biotin was added for 30 min and incubated at 37 °C. After biotin labeling, the cells were washed three to four times with cold DPBS and lysed with 1 ml of lysis buffer. For clarifying, the lysates were ultrasonicated for 15 min in a cold room.

### Heavy 4-HB treatment on C2C12 cells

Treatment of C2C12 cells with heavy 4-HB was performed following published studies^54^. For stable isotope labeling, C2C12 cells were treated with *p*-hydroxy-(aromatic-^13^C6) benzoic acid ([^13^C6] 4-HB) (30 μM, 48 h) in high-glucose DMEM with 10% FBS at 37 °C in 5% CO_2_ (v/v). After treatment, cells were washed with PBS and released from the 150-mm cell culture dish using a cell scraper. Suspended cells were collected by centrifugation and cell pellets were stored at −80 °C.

### CoQ extraction for LC–PRM analysis

Coenzymes Q_9_ and Q_10_, and their reduced forms, were extracted according to a previous study with modifications^72^. Briefly, freeze-dried cells were extracted with 300 µl of methanol using a mixer mill (MM 440, Qiagen) at a frequency of 30 s with three repetitions. Extracts were placed on ice for 15 min and then centrifuged at 12,000*g* at 4 °C for 10 min. The supernatants were transferred to a clean tube. This process was repeated twice. The remaining precipitate was re-extracted with methanol. The pooled supernatants were concentrated under a nitrogen stream, reconstituted with 100 µl of methanol and then subjected to LC–MS/MS.

### O-methylation reaction of COQ3 and RTN4IP1

The O-methylation activity assay was performed as reported previously^51^. The in vitro *O*-methyltransferase activity of COQ3 with RTN4IP1 was measured using synthetic DMeQ_2_ as a substrate. In the assay, 100 μM of substrate and 100 μM of *S*-adenosyl-methionine (as a methyl donor) were resolved into 200 μl of reaction mixture (0.05 M sodium phosphate, pH 7.0 1.0 μM ZnSO_4_). For each condition, 1 μM of purified green fluorescent protein (GFP), RTN4IP1 and COQ3 were added to the mixture. After incubation (37 °C, 1 h), 5 μl of acetic acid was added to terminate the reaction. Samples were concentrated and resuspended in methanol. The samples were subjected to LC–PRM analysis. The activity is presented as a percentage based on each LC–PRM peak area value (CoQ_2_ peak area/(CoQ_2_ peak area + DMeQ_2_ peak area)).

### Climbing assay of dRTN4IP1-KD fruit flies

Flies were maintained on standard cornmeal medium at 25 °C and 50% humidity. *Mef2-GAL4* (BL 27390), *Act5C-GAL4* (BL 4144) and *UAS-dRTN4IP1 RNAi* (VDRC 47264) were obtained from the Bloomington Stock Centerand the Vienna Drosophila Resource Center. In the climbing assay, the percentage of flies that successfully climbed over a height of 8 cm in 10 s was calculated. The 3–5-day male flies were treated with CoQ_2_ containing regular food to a final CoQ_2_ concentration of 50 μg g^−1^ for 24 h.

### Digestion and enrichment of biotinylated peptides

Cold acetone (4 ml) stored at –20 °C was mixed with the lysates and stored at −20 °C for at least 2 h. These samples were centrifuged at 13,000*g* for 10 min at 4 °C and the supernatant was gently discarded. The pellet was resuspended in 500 µl of 8 M urea in 50 mM ammonium bicarbonate. The protein concentration was determined using a BCA assay, after which the protein samples were denatured at 650 rpm for 1 h at 37 °C using a Thermomixer (Eppendorf). Sample reduction and alkylation were individually performed by adding 10 mM dithiothreitol and 40 mM iodoacetamide, respectively, and incubating at 650 rpm for 1 h at 37 °C with the Thermomixer. The samples were diluted eightfold using 50 mM ammonium bicarbonate, after which CaCl_2_ was added for a final concentration of 1 mM. Samples were digested using trypsin (50:1 w/w) at 650 rpm for 6–18 h at 37 °C with the Thermomixer. Insoluble material was removed by centrifuging for 3 min at 10,000*g*. The streptavidin beads (200 μl) were first washed with 2 M urea in 1× TBS three to four times and then added to the samples. The mixture was then rotated for 1 h at room temperature, followed by washing the beads two to three times with 2 M urea in 50 mM ABC; the flow-through fraction was not discarded. After discarding the supernatant, the beads were washed with pure water and transferred to new tubes. After adding 100 μl of 80%, 0.2% trifluoroacetic acid and 0.1% formic acid, the biotinylated peptides were heated at 60 °C and mixed at 650 rpm. The supernatants without streptavidin beads were transferred to new tubes. This elution step was repeated at least four times and then the total elution fractions were dried for 5 h using a Speed-Vac (Eppendorf). The samples were stored at −20 °C before use in LC–MS/MS analyses.

### LC–MS/MS-based proteomic analysis of enriched biotinylated peptide samples

Long analytical capillary columns (100 cm × 75 µm internal diameter) and dual-fritted trap columns (2 cm × 150 µm internal diameter) were packed in house with 3-µm Jupiter C18 particles with a pore size of 300 Å (Phenomenex). The columns were placed in a column heater regulated to a temperature of 45 °C. A NanoAcquity ultrahigh-performance liquid chromatography system (Waters) run in back-flush mode for trapping of samples was operated at a flow rate of 300 nl min^−1^ over 2 h with a linear gradient ranging from 95% solvent A (H_2_O with 0.1% formic acid) to 40% of solvent B (acetonitrile with 0.1% formic acid). The enriched samples were analyzed on an Orbitrap Fusion Lumos mass spectrometer (Thermo Scientific) equipped with an in-house customized nanoelectrospray ion source with the following instrumental parameters: spray voltage, 2.2 kV; capillary temperature, 275 °C and radio frequency lens level, 30.0. The precursor ion scan was operated with a scan range of 300 to 1,500 *m*/*z*; AGC target of 5 × 10^5^; maximum injection time of 50 ms and resolution of 120,000 at 200 *m*/*z*. The MS/MS scan was performed with an isolation width of 1.4 Th, HCD with 30% collision energy, AGC target of 1 × 10^5^, maximum injection time of 200 ms and resolution of 30,000 at 200 *m*/*z*.

### Proteomic data processing and identification of biotinylated peptides

All MS/MS data were searched by MaxQuant (v.1.5.3.30 and v.1.6.2.3) with the Andromeda search engine at a 10 ppm precursor ion mass tolerance against the UniProt mouse reference proteome database (55,152 entries) or the SwissProt *Homo sapiens* proteome database (20,199 entries), according to the sample’s origin. Label-free quantification and match between runs were used with the following search parameters: semitryptic digestion, fixed carbaminomethylation on cysteine, dynamic oxidation of methionine, protein N-terminal acetylation and dynamic DBP labeling (delta monoisotopic mass +331.1896 Da) of tyrosine for APEX2 samples or dynamic biotinylation of lysine (delta monoisotopic mass +226.07759 Da) for TurboID samples. A false discovery rate less than 1% was obtained at the unique labeled peptide level and unique labeled protein level. Label-free quantification intensity values were log-transformed for further analysis and missing values were filled by imputed values representing a normal distribution around the detection limit. To impute the missing value, the intensity distribution of mean and standard deviation was first determined, and then for the imputation values, a new distribution based on Gaussian distribution with a downshift of 1.8 and width of 0.3 standard deviations was created for the total matrix.

### LC–PRM analysis of CoQ molecules

Standard CoQ molecules, including CoQ_2_, CoQ_9_ and CoQ_10_, were purchased from Sigma-Aldrich, and the respective reduced quinol forms were prepared by partial reduction of CoQs via NaCNBH_4_ treatment^72,73^. For the O-methylation in vitro assay, DMeQ_2_ was synthesized in house. The standard molecules were individually analyzed by LC-Orbitrap MS to determine the feature parameters (retention time, precursor ion *m/z* and fragment ion *m*/*z*) for PRM and to optimize the collision energy to maximize the LC–PRM sensitivity. For all CoQs, the fragment ions corresponding to the head group were used to generate the extracted ion chromatograms (LC–PRM chromatograms). By virtue of the high resolution and mass accuracy (less than 10 ppm or less than 2 mDa) of Orbitrap MS in PRM mode, CoQs can be identified and measured without interference in all samples. All extracted CoQ supernatant samples were reconstituted with 40 µl methanol after concentrating with a Speed-Vac (Concentrator plus, Eppendorf), followed by further centrifugation at 12,000*g* at 4 °C for 10 min, and then subjected to LC–PRM analysis using a 1290 infinity II UHPLC system (Agilent) and Orbitrap Exploris 480 MS or Q-Exactive Classic MS system (ThermoFisher Scientific) with a heated electrospray ionization interface. Analytes (10 µl) were injected and separated on a Kinetex C18 column (50 mm long by 2.1 mm internal diameter, 1.3 µm, 100 Å; Phenomenex) for Orbitrap Exploris 480 MS or a ZORBAX RRHD Extend-C18 column (50 mm long and 2.1 mm internal diameter, 1.8 µm, 80 Å; Agilent) for Q-Exactive Classic MS. Mobile phase A (2 mM ammonium acetate in 100% LC–MS-grade water) and mobile phase B (2 mM ammonium acetate in 100% LC–MS-grade methanol) were used at a flow rate of 300 µl min^−1^ to generate a gradient under the following isocratic separation conditions: 0 min, 50% B; 0.01 min, 75% B; 3.01 min, 100% B; 5 min, 100% B; 5.01 min, 50% B; 8 min, 50% B for CoQ_2_ and DMeQ_2_ analysis, and 0 min, 50% B; 0.01 min, 75% B; 3.01 min, 100% B; 12 min, 100% B; 12.01 min, 50% B; 15 min, 50% B for CoQ_9_ and CoQ_10_ analysis. Electrospray ionization was operated in positive-ion mode at 4 kV under the following conditions: ion transfer tube temperature, 325 °C; sheath gas, 35 a.u; auxiliary gas, 5 a.u. Targeted MS/MS mode was used for the inclusion list containing precursor ions and the corresponding collision energy value under a 30,000 Orbitrap resolution and 110 ms injection time for Orbitrap Exploris 480 MS or a 35,000 Orbitrap resolution and 120 ms injection time for Q-Exactive Classic MS. Figure 4e was obtained by Q-Exactive Classic MS coupled with a ZORBAX RRHD Extend-C18 column, and all other data were obtained from Orbitrap Exploris 480 MS coupled with a Kinetex C18 column. LC–PRM feature parameters for all CoQs in both instrumental settings are summarized in Supplementary Table 4. Quantitation of targeted CoQs was conducted based on chromatographic peak area of fragment ions (obtained by Qual Browser in Xcalibur Software, ThermoFisher Scientific) for each analyte at an adequate retention time window.

### Synthesis of (*E*)-2-(3,7-dimethylocta-2,6-dien-1-yl)-5-hydroxy-6-methoxy-3-methylcyclohexa-2,5-diene-1,4-dione (2) and DMeQ_2_

The Freidel-Crafts allylation of fumigatin was carried out according to the established procedure (1), with subsequent adjustments. Fumigatin (300 mg, 1.77 mmol) was dissolved in a mixture of ether and ethanol (1:1, 60 ml), followed by dropwise addition of Na_2_S_2_O_4_ (10% in H_2_O) to the stirred solution until the mixture was decolorized^74,75^. On decolorization, ether (50 ml) was introduced and the resulting organic layer underwent three washes with brine. The solution was then dried over MgSO_4_ and concentrated under vacuum. The resulting fumigatin hydroquinone was dissolved in freshly distilled 1,4-dioxane (60 ml) under an argon atmosphere. To this solution, geraniol (817 mg, 5.3 mmol) was added, followed by Bf_3_OEt_2_ (8.9 ml, 6.3 mmol). The mixture was left to react at room temperature for 18 h. Postreaction, the mixture underwent brine washing and subjected to three ether extractions. The resulting organic layers were dried using MgSO_4_, filtered and concentrated under vacuum conditions. The crude product was dissolved in ether (30 ml) and treated with excess FeCl_3_ in a mixture of water and methanol (1:1) for 30 min. The resulting mixture underwent three ether extractions, followed by drying over MgSO_4_, filtration and concentration using rotary evaporation. The crude product was then subjected to purification on a Florisil column using the following gradient system: 4:1 hexane/ethyl acetate, 1:1 hexane/ethyl acetate, 100% ethyl acetate, 4:1 hexane/ethyl acetate and 4:1 hexane/ethyl acetate containing 1% glacial acetic acid. The desired product (demethylhyl-Q2) was retained as a purple compound at the column’s upper portion until the concluding wash, which involved 1% glacial acetic acid. After being exposed to acetic acid, the desired compound’s color shifted from purple to red orange, prompting its subsequent elution from the column. Nuclear magnetic resonance characterization of the yellow-orange oil (110 mg, 20% yield) matched with previously reported data (2): 1H nuclear magnetic resonance (CDCl3, 400 MHz): *δ* = 6.49 (s, 1H), 5.05 (br., s, 1H), 4.91 (t, 3JH − H = 6.6 Hz, 1H), 4.07 (s, 3H), 3.20 (d, 3JH − H = 7.0 Hz, 2H), 2.06 (m, 2H), 2.04 (s, 3H), 1.97 (m, 2H), 1.74 (s, 3H), 1.65 (s, 3H) and 1.58 (s, 3H) ppm.

### APEX2 electron microscopy (MTS-V5-APEX2 and RTN4IP1-V5-APEX2)

To observe the DAB-stained mitochondria in the muscle tissues of MAX-Tg mice, the dissected tissues were fixed with 2.5% glutaraldehyde and 2% paraformaldehyde in 0.1 M cacodylate solution (pH 7.2) for 1 h at 4 °C. After washing, 20 mM glycine solution was used to quench the unreacted aldehyde. DAB staining was performed for approximately 40 min until a light brown stain was visible under a stereomicroscope. DAB-stained tissues were postfixed with 2% osmium tetroxide in distilled water for 60 min at 4 °C, set en bloc in 1% uranyl acetate overnight and dehydrated with a graded acetone series. The samples were then embedded with an Embed-812 embedding kit and polymerized in an oven at 60 °C. The polymerized samples were sectioned (60 nm) with an ultramicrotome (UC7; Leica Microsystems) and the sections were mounted on copper slot grids with a specimen support film. Sections were stained with uranyless and lead citrate and then observed on a Tecnai G2 transmission electron microscope (ThermoFisher).

To visualize the subcellular localization of the transiently expressed RTN4IP1-V5-APEX2, HEK293T cells were cultured in 35-mm glass grid-bottomed culture dishes (MatTek Life Sciences) to 30–40% confluency. The cells were then transfected with RTN4IP1-V5-APEX2 using Lipofectamine 2000. The next day, the cells were fixed with 2.5% glutaraldehyde and 2% paraformaldehyde in 0.1 M cacodylate solution (pH 7.2) for 1 h at 4 °C. After washing, 20 mM glycine solution was used to quench unreacted aldehyde. DAB staining was performed for approximately 20–40 min until a light brown stain was visible under an inverted light microscope. DAB-stained cells were postfixed with 2% osmium tetroxide in distilled water for 30 min at 4 °C and dehydrated with a graded ethanol series. The samples were then embedded with the Embed-812 embedding kit and polymerized in an oven at 60 °C. The polymerized samples were sectioned (60 nm) with an ultramicrotome (UC7; Leica Microsystems), and the sections were mounted on copper slot grids with a specimen support film. Sections were stained with uranyless and lead citrate and then viewed on a Tecnai G2 transmission electron microscope (ThermoFisher).

### Electron microscopy of control and Rtn4ip1-KO C2C12 cells

Cells were grown in 35-mm glass-bottomed culture dishes to 50–60% confluency. The cells were then fixed with 2 ml of the fixative solution containing 2% paraformaldehyde and 2.5% of glutaraldehyde diluted in 0.1 M sodium cacodylate buffer. After washing, the cells were postfixed in 2% osmium tetroxide containing 1.5% potassium ferrocyanide for 1 h at 4 °C. The fixed cells were dehydrated using an ethanol series (50, 60, 70, 80, 90 and 100%) for 10 min at each concentration and infiltrated with an embedding medium. After embedment, 60-nm sections were cut horizontally to the plane of the block (UC7; Leica Microsystems) and were mounted on copper slot grids with a specimen support film. The sections were then double stained with uranyless and lead citrate and viewed on a Tecnai G2 transmission electron microscope (ThermoFisher).

### Electron microscope imaging of control and RTN4IP1-KD fruit flies

Adult flies were collected directly into the 0.1 M sodium cacodylate buffer (pH 7.2) at 4 °C, followed by careful removal of the legs, head and abdomen using a dissection microscope. Thoraxes were fixed with 2.5% glutaraldehyde and 2% paraformaldehyde in 0.1 M cacodylate solution (pH 7.2) overnight at 4 °C. After washing, the samples were postfixed in 2% osmium tetroxide containing 1.5% potassium ferrocyanide for 1 h at 4 °C. The fixed samples were dehydrated using an acetone series (50, 60, 70, 80, 90 and 100%) for 20 min at each concentration and infiltrated with an embedding medium (EM812). The polymerized samples were sectioned (60 nm) with an ultramicrotome (UC7; Leica Microsystems) and the sections were mounted on copper slot grids with a specimen support film. Sections were stained with uranyless and lead citrate and viewed on a Tecnai G2 transmission electron microscope (ThermoFisher).

### Expression plasmids

The genes were incorporated into designated vectors through conventional enzymatic restriction digestion, followed by ligation using T4 DNA ligase. For the creation of constructs containing brief tags (for example, V5 epitope tag) or signal sequences attached to the protein, the respective tag was included within the primers used for PCR amplification of the gene. Subsequently, the PCR products were subjected to digestion with restriction enzymes and then ligated into prepared vectors (that is, pcDNA3, pcDNA5 and pET-21a). Throughout the process, the cytomegalovirus promoter was consistently used for the purpose of expressing the genes in mammalian cells.

### Ethical statement

Animal studies were approved by the Institutional Animal Care and Use Committee of Seoul National University. Mice were maintained under a 12-h light-dark cycle in a climate-controlled specific pathogen-free facility in Seoul National University. Standard chow diet and water were provided ad libitum.

### Reporting summary

Further information on research design is available in the Nature Portfolio Reporting Summary linked to this article.

## Online content

Any methods, additional references, Nature Portfolio reporting summaries, source data, extended data, supplementary information, acknowledgements, peer review information; details of author contributions and competing interests; and statements of data and code availability are available at 10.1038/s41589-023-01452-w.

### Supplementary information

Supplementary Information Supplementary Figs. 1–9 and Tables 1–6.

Reporting Summary

Supplementary Video Video for climbing test.

Supplementary Data 1 DBP-labeled peptides and proteins in MAX-Tg.

Supplementary Data 2 DBP-labeled proteins by MTS-APEX2 in HEK293T and in muscle tissues.

Supplementary Data 3 Tissue-specific DBP-labeled proteins in MAX-Tg.

Supplementary Data 4 Muscle tissue-specific DBP-labeled proteins in Myf5-Cre; LSL-MAX-Tg.

Supplementary Data 5 DBP-labeled proteins from MAX-Tg and Myf5-Cre; LSL-MAX-Tg mouse and mitoplast proteins from WT mouse.

Supplementary Data 6 Interactome of RTN4IP1 revealed by TurboID.

### Source data

Source Data Fig. 1 Unmodified western blot.

Source Data Fig. 3 Plate reader raw data.

Source Data Fig. 4 LC–PRM raw data.

Source Data Fig. 5 Foci counting data, Plate reader raw data and OCR raw data.

Source Data Fig. 5 Unmodified western blot.

Source Data Fig. 6 LC–PRM raw data, Climbing assay recording data.

Source Data Extended Data Fig. 2 Unmodified western blot.

Source Data Extended Data Fig. 3 Unmodified western blot.

Source Data Extended Data Fig. 4 Unmodified western blot.

Source Data Extended Data Fig. 6 LC–PRM raw data.

Source Data Extended Data Fig. 8 OCR raw data.

Source Data Extended Data Fig. 9 LC–PRM raw data.

## Data Availability

Further information and requests for resources and reagents should be directed to and will be fulfilled by the lead contact, H.W.R. (rheehw@snu.ac.kr). On reasonable request, unique reagents used in this paper can be provided. The structural information was referred to from the PDB under the following accession numbers: 2VN8 and 1QOR. The coexpression relevance data for RTN4IP1-COQ3 was retrieved from the ARCHS4 database (https://maayanlab.cloud/archs4/) and the DepMap database (https://depmap.org/portal/). Transcriptome data for human organs were acquired from the GTEx portal (https://gtexportal.org/). The MS proteomics data have been deposited to the ProteomeXchange Consortium (http://proteomecentral.proteomexchange.org) via the PRIDE partner repository with the dataset identifier PXD026793. Source data are provided with this paper.

## References

[1] Uhlen M (2015). Tissue-based map of the human proteome. Science.

[2] Uhlen M (2019). A genome-wide transcriptomic analysis of protein-coding genes in human blood cells. Science.

[3] Zick M, Rabl R, Reichert AS (2009). Cristae formation-linking ultrastructure and function of mitochondria. Biochim. Biophys. Acta.

[4] Pette D, Vrbová G (1999). What does chronic electrical stimulation teach us about muscle plasticity. Muscle Nerve.

[5] Williams EG (2018). Quantifying and localizing the mitochondrial proteome across five tissues in a mouse population. Mol. Cell Proteom..

[6] Mootha VK (2003). Integrated analysis of protein composition, tissue diversity, and gene regulation in mouse mitochondria. Cell.

[7] Bayraktar, E. C. et al. MITO-Tag mice enable rapid isolation and multimodal profiling of mitochondria from specific cell types in vivo. *Proc. Natl Acad. Sci. USA* **116**, 303–312 (2019). 10.1073/pnas.1816656115 PMC6320505 30541894

[8] Busch JD (2019). MitoRibo-tag mice provide a tool for in vivo studies of mitoribosome composition. Cell Rep..

[9] Yoo C-M, Rhee H-W (2020). APEX, a master key to resolve membrane topology in live cells. Biochemistry.

[10] Lee S-Y (2017). Architecture mapping of the inner mitochondrial membrane proteome by chemical tools in live cells. J. Am. Chem. Soc..

[11] Silva J (2018). EXD2 governs germ stem cell homeostasis and lifespan by promoting mitoribosome integrity and translation. Nat. Cell Biol..

[12] Park J (2019). The structure of human EXD2 reveals a chimeric 3′ to 5′ exonuclease domain that discriminates substrates via metal coordination. Nucleic Acids Res..

[13] Rhee H-W (2013). Proteomic mapping of mitochondria in living cells via spatially restricted enzymatic tagging. Science.

[14] Lobingier BT (2017). An approach to spatiotemporally resolve protein interaction networks in living. Cells Cell.

[15] Lam SS (2015). Directed evolution of APEX2 for electron microscopy and proximity labeling. Nat. Methods.

[16] Dumrongprechachan, V. et al. Cell-type and subcellular compartment-specific APEX2 proximity labeling reveals activity-dependent nuclear proteome dynamics in the striatum. *Nat. Commun.* **12**, 4855 (2021). 10.1038/s41467-021-25144-y PMC8357913 34381044

[17] Hobson BD (2022). Subcellular proteomics of dopamine neurons in the mouse brain. eLife.

[18] Hung V (2014). Proteomic mapping of the human mitochondrial intermembrane space in live cells via ratiometric APEX tagging. Mol. Cell.

[19] Udeshi ND (2017). Antibodies to biotin enable large-scale detection of biotinylation sites on proteins. Nat. Methods.

[20] Kwak C (2020). Contact-ID, a tool for profiling organelle contact sites, reveals regulatory proteins of mitochondrial-associated membrane formation. Proc. Natl Acad. Sci. USA.

[21] Cho KF (2020). Split-TurboID enables contact-dependent proximity labeling in cells. Proc. Natl Acad. Sci. USA.

[22] Calvo SE, Clauser KR, Mootha VK (2016). MitoCarta2.0: an updated inventory of mammalian mitochondrial proteins. Nucleic Acids Res..

[23] Rath S (2021). MitoCarta3.0: an updated mitochondrial proteome now with sub-organelle localization and pathway annotations. Nucleic Acids Res..

[24] Rahbani JF (2021). Creatine kinase B controls futile creatine cycling in thermogenic fat. Nature.

[25] Martell JD (2012). Engineered ascorbate peroxidase as a genetically encoded reporter for electron microscopy. Nat. Biotechnol..

[26] Picard M, White K, Turnbull DM (2012). Mitochondrial morphology, topology, and membrane interactions in skeletal muscle: a quantitative three-dimensional electron microscopy study. J. Appl. Physiol..

[27] Fukasawa Y (2015). MitoFates: improved prediction of mitochondrial targeting sequences and their cleavage sites. Mol. Cell. Proteom..

[28] Ghezzi, D. & Zeviani, M. in *Mitochondrial Oxidative Phosphorylation: Nuclear-Encoded Genes, Enzyme Regulation, and Pathophysiology* (ed. Kadenbach, B.) 65–106 (Springer, 2012).

[29] Borna NN (2019). Mitochondrial ribosomal protein PTCD3 mutations cause oxidative phosphorylation defects with Leigh syndrome. Neurogenetics.

[30] Newman AC, Maddocks ODK (2017). One-carbon metabolism in cancer. Br. J. Cancer.

[31] Altman BJ, Stine ZE, Dang CV (2016). From Krebs to clinic: glutamine metabolism to cancer therapy. Nat. Rev. Cancer.

[32] Staron RS (1999). Fiber type composition of four hindlimb muscles of adult Fisher 344 rats. Histochem. Cell Biol..

[33] Lopaschuk GD, Ussher JR, Folmes CDL, Jaswal JS, Stanley WC (2010). Myocardial fatty acid metabolism in health and disease. Physiol. Rev..

[34] Oizel K (2015). D-2-Hydroxyglutarate does not mimic all the IDH mutation effects, in particular the reduced etoposide-triggered apoptosis mediated by an alteration in mitochondrial NADH. Cell Death Dis..

[35] Gollnick PD, Sjödin B, Karlsson J, Jansson E, Saltin B (1974). Human soleus muscle: a comparison of fiber composition and enzyme activities with other leg muscles. Pflügers Arch..

[36] Jang YC (2012). Dietary restriction attenuates age-associated muscle atrophy by lowering oxidative stress in mice even in complete absence of CuZnSOD. Aging Cell.

[37] Mohiuddin M (2019). Critical limb ischemia induces remodeling of skeletal muscle motor unit, myonuclear-, and mitochondrial-domains. Sci. Rep..

[38] Morgenstern M (2017). Definition of a high-confidence mitochondrial proteome at quantitative scale. Cell Rep..

[39] Angebault C (2015). Recessive mutations in RTN4IP1 cause isolated and syndromic optic neuropathies. Am. J. Hum. Genet..

[40] Charif M (2018). Neurologic phenotypes associated with mutations in RTN4IP1 (OPA10) in children and young adults. JAMA Neurol..

[41] Thorn JM, Barton JD, Dixon NE, Ollis DL, Edwards KJ (1995). Crystal structure of *Escherichia coli* QOR quinone oxidoreductase complexed with NADPH. J. Mol. Biol..

[42] Taneja B, Mande SC (1999). Conserved structural features and sequence patterns in the GroES fold family. Protein Eng..

[43] Murzin AG (1996). Structural classification of proteins: new superfamilies. Curr. Opin. Struct. Biol..

[44] Sillitoe I (2019). CATH: expanding the horizons of structure-based functional annotations for genome sequences. Nucleic Acids Res..

[45] Bongard RD, Lindemer BJ, Krenz GS, Merker MP (2009). Preferential utilization of NADPH as the endogenous electron donor for NAD(P)H:quinone oxidoreductase 1 (NQO1) in intact pulmonary arterial endothelial cells. Free Radic. Biol. Med..

[46] Bongard RD (2011). Characterization of the threshold for NAD(P)H:quinone oxidoreductase activity in intact sulforaphane-treated pulmonary arterial endothelial cells. Free Radic. Biol. Med.

[47] Merker MP, Audi SH, Bongard RD, Lindemer BJ, Krenz GS (2006). Influence of pulmonary arterial endothelial cells on quinone redox status: effect of hyperoxia-induced NAD(P)H:quinone oxidoreductase 1. Am. J. Physiol. Lung Cell. Mol. Physiol..

[48] Wu CY, Hwa YH, Chen YC, Lim C (2012). Hidden relationship between conserved residues and locally conserved phosphate-binding structures in NAD(P)-binding proteins. J. Phys. Chem. B.

[49] Branon TC (2018). Efficient proximity labeling in living cells and organisms with TurboID. Nat. Biotechnol..

[50] Lee SY, Seo JK, Rhee HW (2019). Direct identification of biotinylated proteins from proximity labeling (Spot-BioID). Methods Mol. Biol..

[51] Poon WW (1999). Yeast and rat Coq3 and Escherichia coli UbiG polypeptides catalyze both O-methyltransferase steps in coenzyme Q biosynthesis. J. Biol. Chem..

[52] Jonassen T, Clarke CF (2000). Isolation and functional expression of human COQ3, a gene encoding a methyltransferase required for ubiquinone biosynthesis. J. Biol. Chem..

[53] Peterson AC, Russell JD, Bailey DJ, Westphall MS, Coon JJ (2012). Parallel reaction monitoring for high resolution and high mass accuracy quantitative, targeted proteomics. Mol. Cell. Proteom..

[54] Xie LX (2015). Resveratrol and *para*-coumarate serve as ring precursors for coenzyme Q biosynthesis [S]. J. Lipid Res..

[55] Fernández-Del-Río L, Clarke CF (2021). Coenzyme Q biosynthesis: an update on the origins of the benzenoid ring and discovery of new ring precursors. Metabolites.

[56] Bersuker K (2019). The CoQ oxidoreductase FSP1 acts parallel to GPX4 to inhibit ferroptosis. Nature.

[57] Mellors A, Tappel AL (1966). The inhibition of mitochondrial peroxidation by ubiquinone and ubiquinol. J. Biol. Chem..

[58] Doll S (2017). ACSL4 dictates ferroptosis sensitivity by shaping cellular lipid composition. Nat. Chem. Biol..

[59] Friedmann Angeli JP (2014). Inactivation of the ferroptosis regulator Gpx4 triggers acute renal failure in mice. Nat. Cell Biol..

[60] Lewerenz J, Ates G, Methner A, Conrad M, Maher P (2018). Oxytosis/ferroptosis-(Re-) emerging roles for oxidative stress-dependent non-apoptotic cell death in diseases of the central nervous system. Front. Neurosci..

[61] Höglinger D (2019). NPC1 regulates ER contacts with endocytic organelles to mediate cholesterol egress. Nat. Commun..

[62] Zaki NM (2016). Strategies for oral delivery and mitochondrial targeting of CoQ10. Drug Deliv..

[63] Daigle TL (2018). A suite of transgenic driver and reporter mouse lines with enhanced brain-cell-type targeting and functionality. Cell.

[64] Hu W-H (2002). Identification and characterization of a novel Nogo-interacting mitochondrial protein (NIMP). J. Neurochem..

[65] Barkovich RJ (1997). Characterization of the *COQ5* Gene from *Saccharomyces cerevisiae* evidence for a C-methyltransferase in ubiquinone biosynthesis. J. Biol. Chem..

[66] Jumper J (2021). Highly accurate protein structure prediction with AlphaFold. Nature.

[67] Fujii M, Yasuda K, Hartman PS, Ayusawa D, Ishii N (2011). A mutation in a mitochondrial dehydrogenase/reductase gene causes an increased sensitivity to oxidative stress and mitochondrial defects in the nematode *Caenorhabditis elegans*. Genes Cells.: Devoted Mol. Cell. Mech..

[68] Birrell GW (2002). Transcriptional response of *Saccharomyces cerevisiae* to DNA-damaging agents does not identify the genes that protect against these agents. Proc. Natl Acad. Sci. USA.

[69] Zou XH (2019). Whole exome sequencing identifies two novel mutations in the reticulon 4-Interacting Protein 1 gene in a Chinese family with autosomal recessive optic neuropathies. J. Mol. Neurosci..

[70] Giacomini T (2020). Optic atrophy and generalized chorea in a patient harboring an OPA10/RTN4IP1 pathogenic variant. Neuropediatrics.

[71] D’Gama AM (2021). Exome sequencing identifies novel missense and deletion variants in RTN4IP1 associated with optic atrophy, global developmental delay, epilepsy, ataxia, and choreoathetosis. Am. J. Med. Genet. Part A.

[72] Tang Z (2014). Rapid assessment of the coenzyme Q10 redox state using ultrahigh performance liquid chromatography tandem mass spectrometry. Analyst.

[73] Burger N (2020). A sensitive mass spectrometric assay for mitochondrial CoQ pool redox state in vivo. Free Radic. Biol. Med.

[74] Nagase H, Woessner JF (1999). Matrix metalloproteinases. J. Biol. Chem..

[75] van der Klei, A., de Jong, R. L. P., Lugtenburg, J. & Tielens, A. G. M. Synthesis and spectroscopic characterization of [1′-14C]-ubiquinone-2,[1′-14C]-5-demethoxy-5-hydroxyubiquinone-2, and [1′-14C]-5-demethoxyubiquinone-2. *Eur. J. Organ. Chem.* **2002**, 3015–3023 (2002).

