# Supplementary material for: Mitochondrial matrix RTN4IP1/OPA10 is an oxidoreductase for coenzyme Q synthesis
Source: Nat Chem Biol. 2023 Oct 26;20(2):221–33. doi: 10.1038/s41589-023-01452-w (PMC10830421; doi:10.1038/s41589-023-01452-w)
Supplement: Supplementary file 1 — Supplementary Figs. 1–9 and Tables 1–6. [file 41589_2023_1452_MOESM1_ESM.pdf]

nature chemical biology

Article

https://doi.org/10.1038/s41589-023-01452-w

Mitochondrial matrix RTN4IP1/OPA10 is an oxidoreductase for coenzyme Q synthesis

In the format provided by the authors and unedited

Supplementary Information

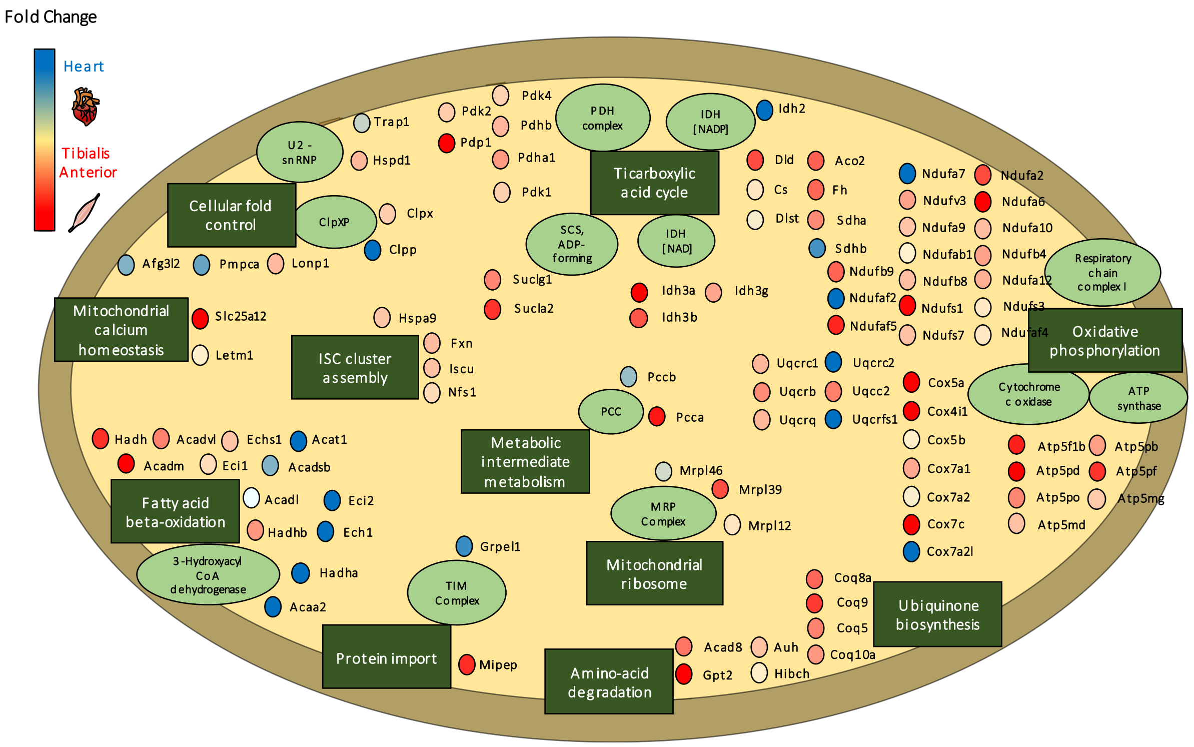

Supplementary Fig. 1 Heart and TA muscle-enriched proteins

Heart and TA muscle-enriched proteins are in blue and red, respectively. All proteins are color-coded to reflect the fold change in the average intensity between the TA muscle and heart. Annotations with function and complex are based on information from UniProt and CORUM. See **Supplementary Data 3** for detailed information.

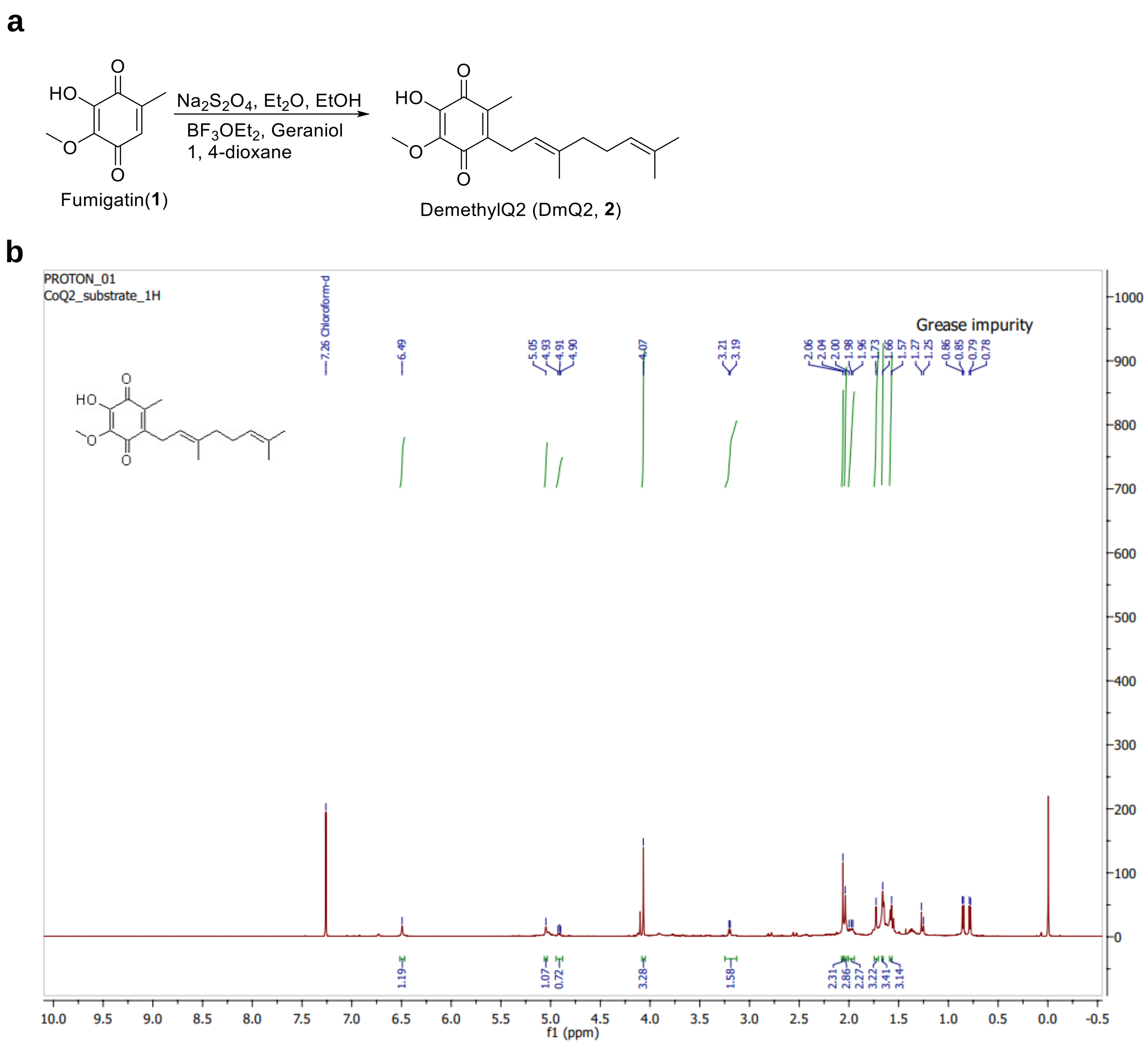

Supplementary Fig. 2 Synthesis of DMeCoQ$_2$

(**a**) Scheme for the synthesis of DMeCoQ$_2$. This scheme is followed by (10419476 DOI: 10.1074/jbc.274.31.21665) (**b**) 1H NMR characterization of DMeCoQ$_2$ (CDCl3, 400 MHz): δ = 6.49 (s, 1H), 5.05 (br., s, 1H), 4.91 (t, 3JH−H = 6.6 Hz, 1H), 4.07 (s, 3H), 3.20 (d, 3JH−H = 7.0 Hz, 2H), 2.06 (m, 2H), 2.04 (s, 3H), 1.97 (m, 2H), 1.74 (s, 3H), 1.65 (s, 3H), 1.58 (s, 3H) ppm. This data matches with the previously reported data[77].

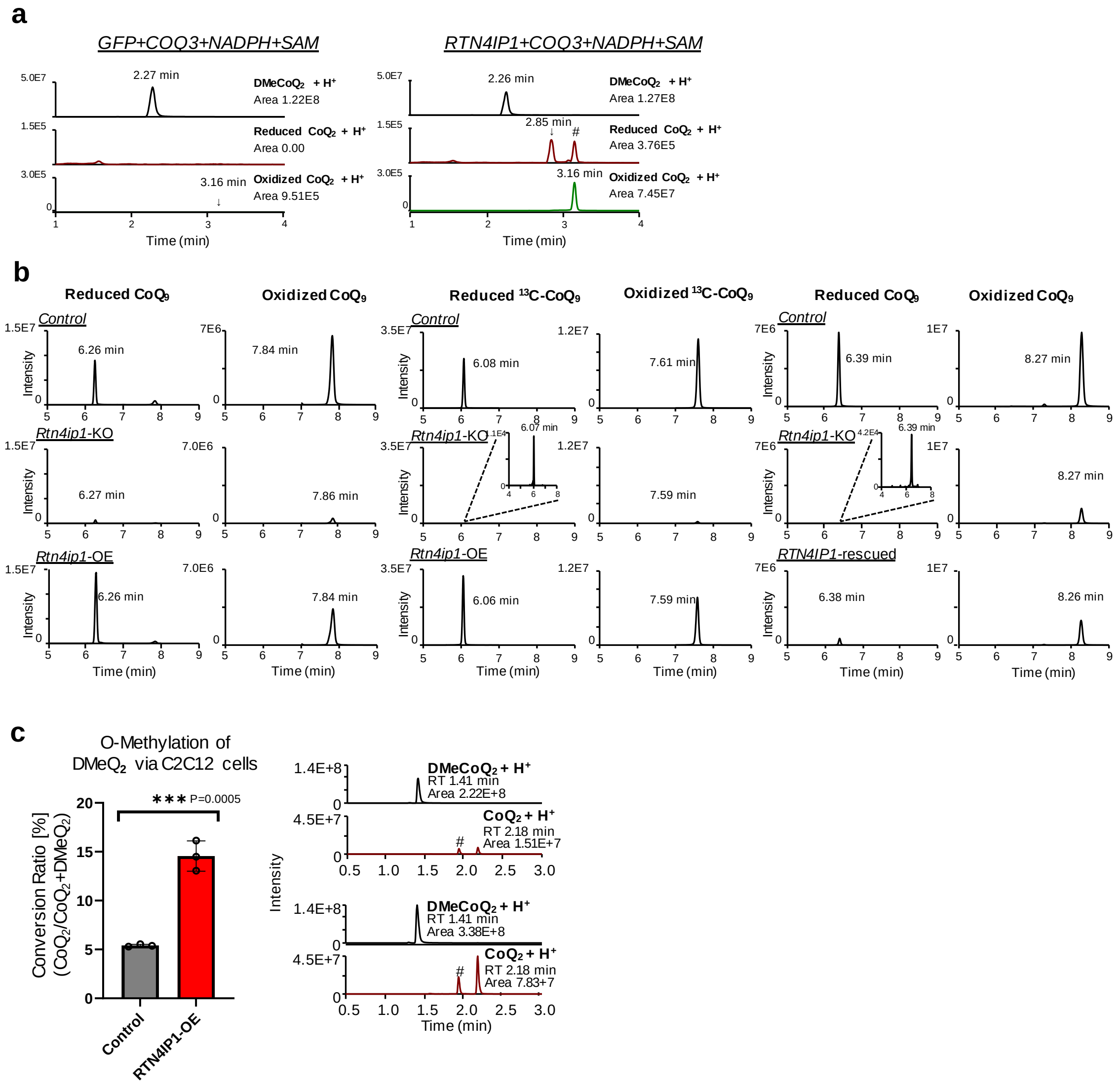

Supplementary Fig. 3 Representative LC-PRM chromatograms

(**a**) LC-PRM chromatograms of measured CoQ_2 and $DMeQ_2$ are shown representing RTN4IP1-assisted O-methyltransferase activity of COQ3. Second isotope of oxidized CoQ_2 is marked with # in the chromatogram. (**b**) Representative LC-PRM chromatograms of endogenous or *de novo* synthesized CoQ_9 and CoQ_9H_2. (**c**) Histogram of LC-PRM assay results and representative LC-PRM chromatograms of DMeQ2/CoQ2 in DMeQ2-treated (30 μM, 48 h) C2C12 cells (i.e. control and RTN4IP1-OE). Y-axis of histogram indicates the measured conversion ratio (i.e. [CoQ2] / [DMeQ2 and CoQ2]). Uncharacterized metabolite showing identical ion reaction pattern (m/z 319.19 → 197.081) with CoQ2 (2.18 min) at different retention time (1.94 min), which may be one of possible structural isoforms of CoQ2, is marked with # in the chromatogram. (n=3 biological replicates) A fixed Y-axis scale was used for all LC-PRM chromatograms for the respective panels. Mean values are shown with error bars representing the standard deviation. Statistical significance was determined using a two-tailed Student's t-test: $*p < 0.05$, $**p < 0.01$, $***p < 0.001$. Source data can be found in the Source Data file

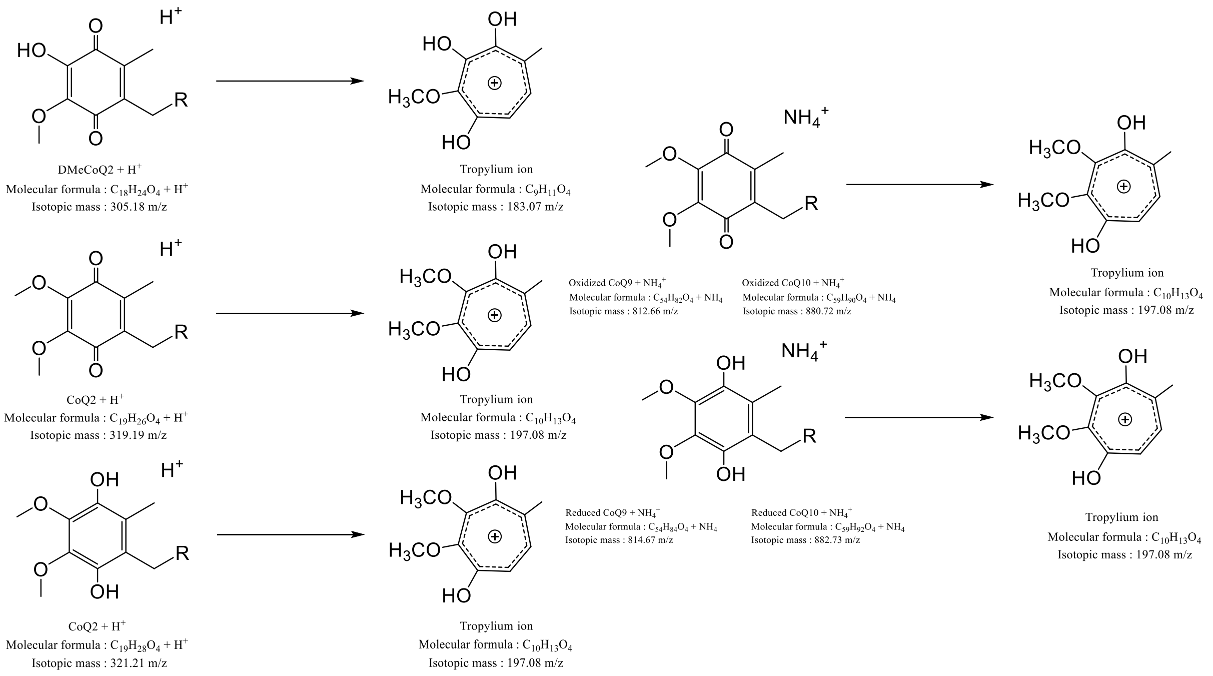

Supplementary Fig. 4 Expected fragmentation reaction of CoQ molecules

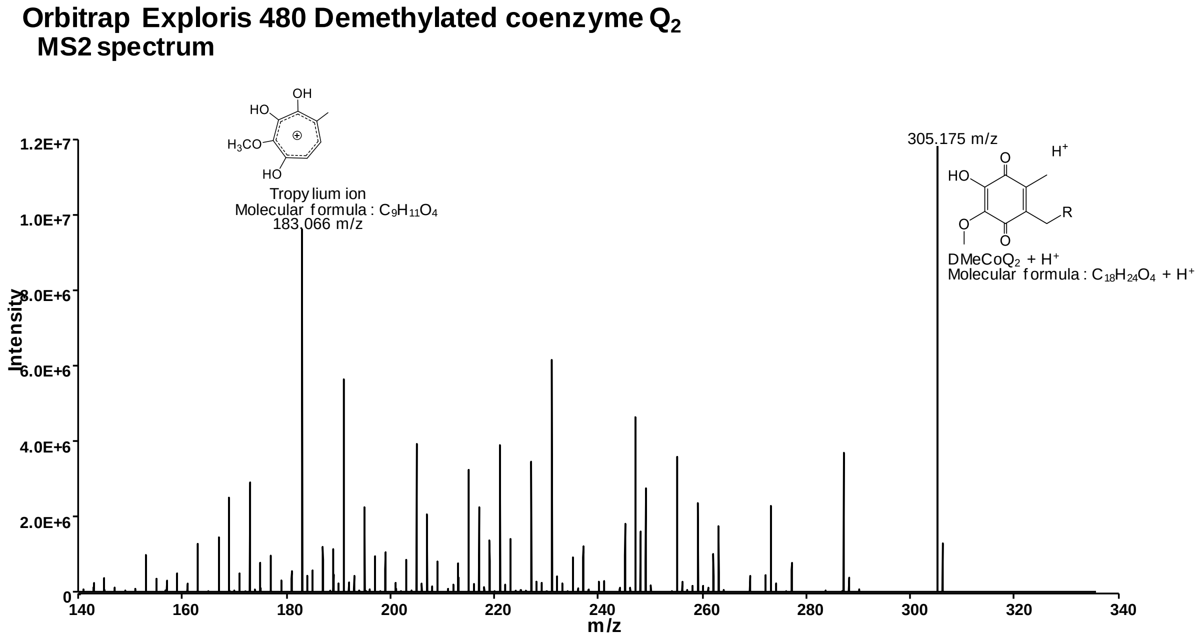

Supplementary Fig. 5 MS2 Spectra for DMeQ2

Orbitrap Exploris 480 Reduced coenzyme Q_2
MS2 spectrum

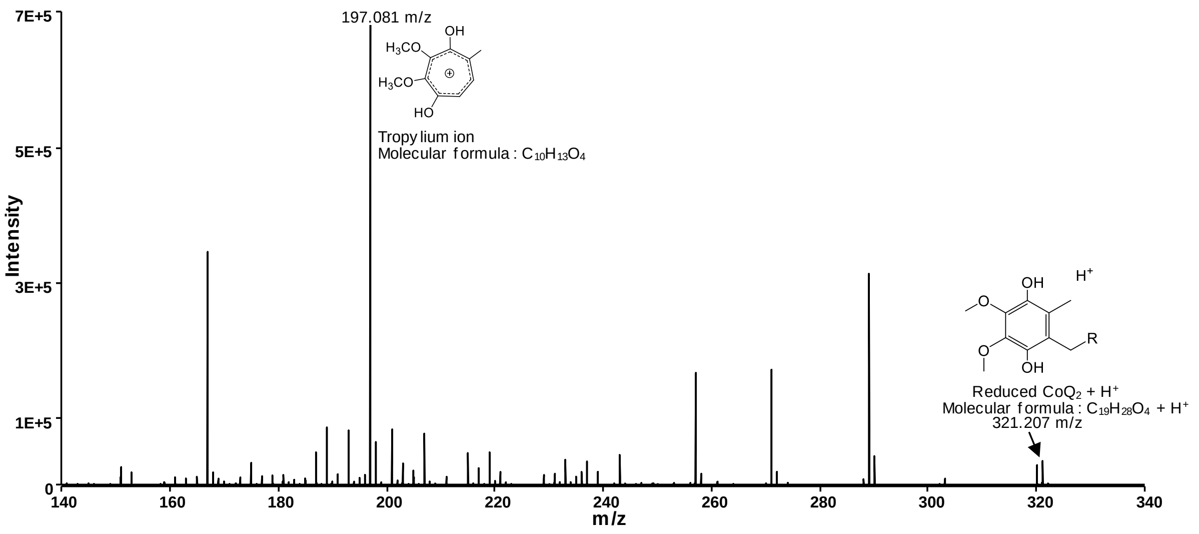

Orbitrap Exploris 480 Oxidized coenzyme Q_2
MS2 spectrum

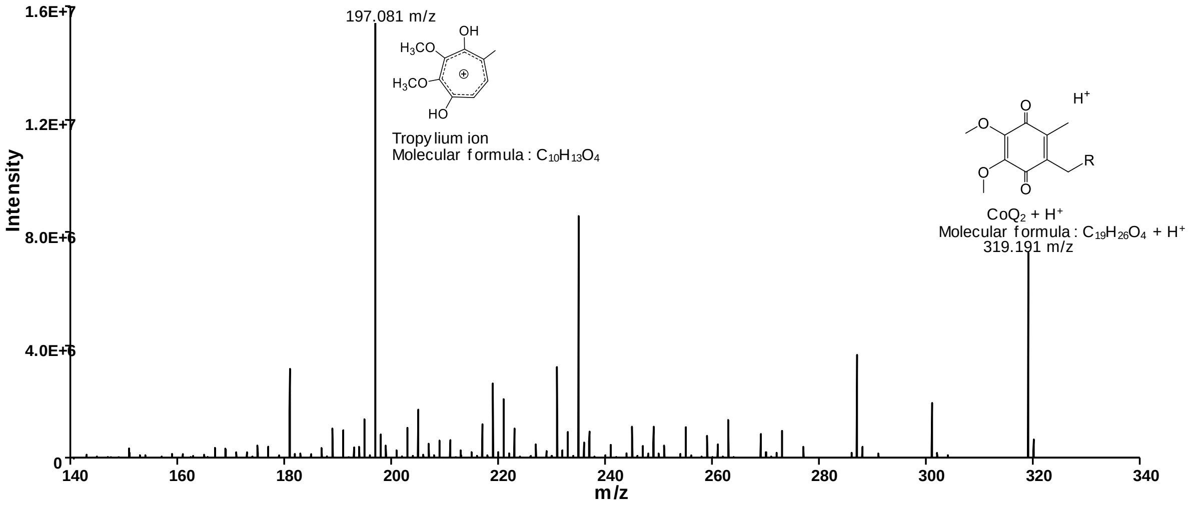

Supplementary Fig. 6 MS2 Spectra for CoQ2

Orbitrap Exploris 480 Reduced coenzyme Q_9 MS2 spectrum

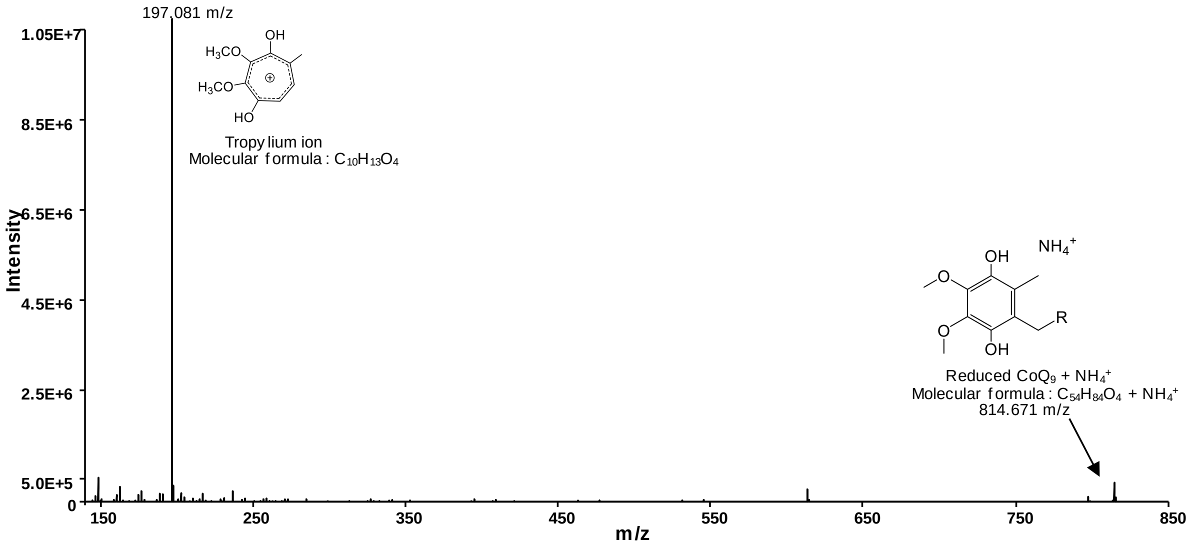

Orbitrap Exploris 480 Oxidized coenzyme Q_9 MS2 spectrum

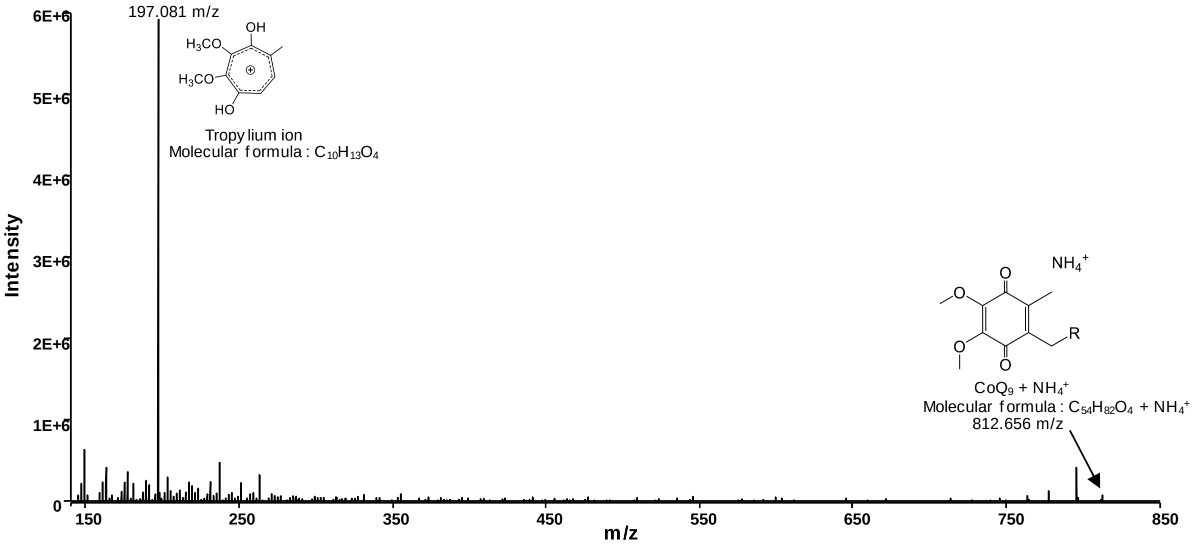

Supplementary Fig. 7 MS2 Spectra for CoQ9

Orbitrap Exploris 480 Reduced coenzyme Q_{10}
MS2 spectrum

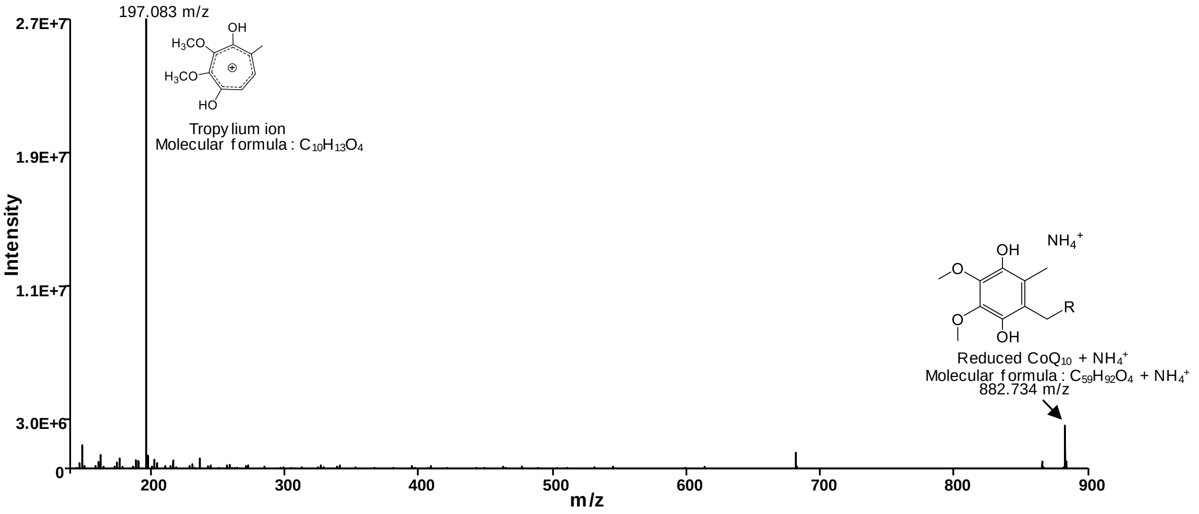

Orbitrap Exploris 480 Oxidized coenzyme Q_{10}
MS2 spectrum

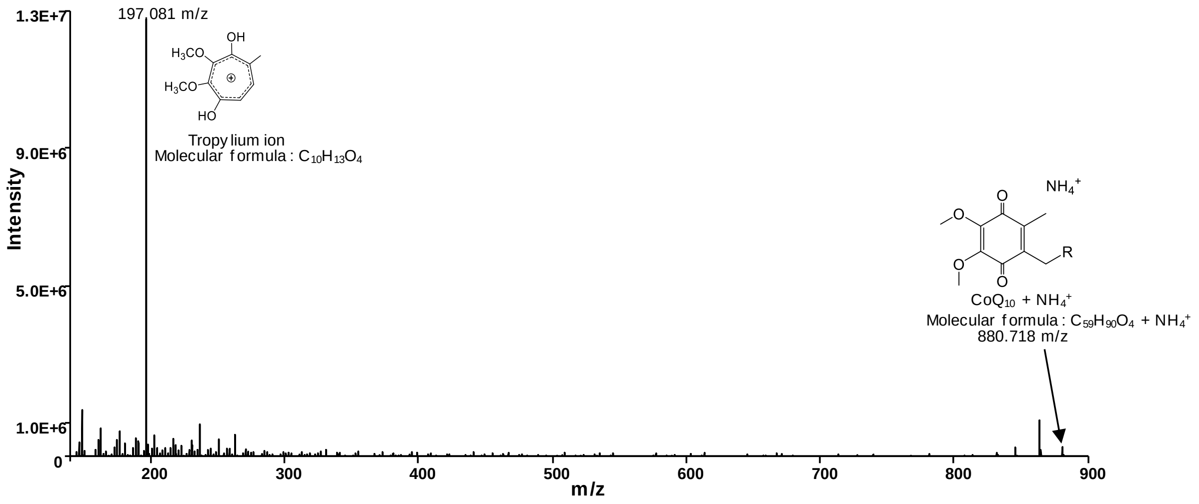

Supplementary Fig. 8 MS2 Spectra for CoQ10

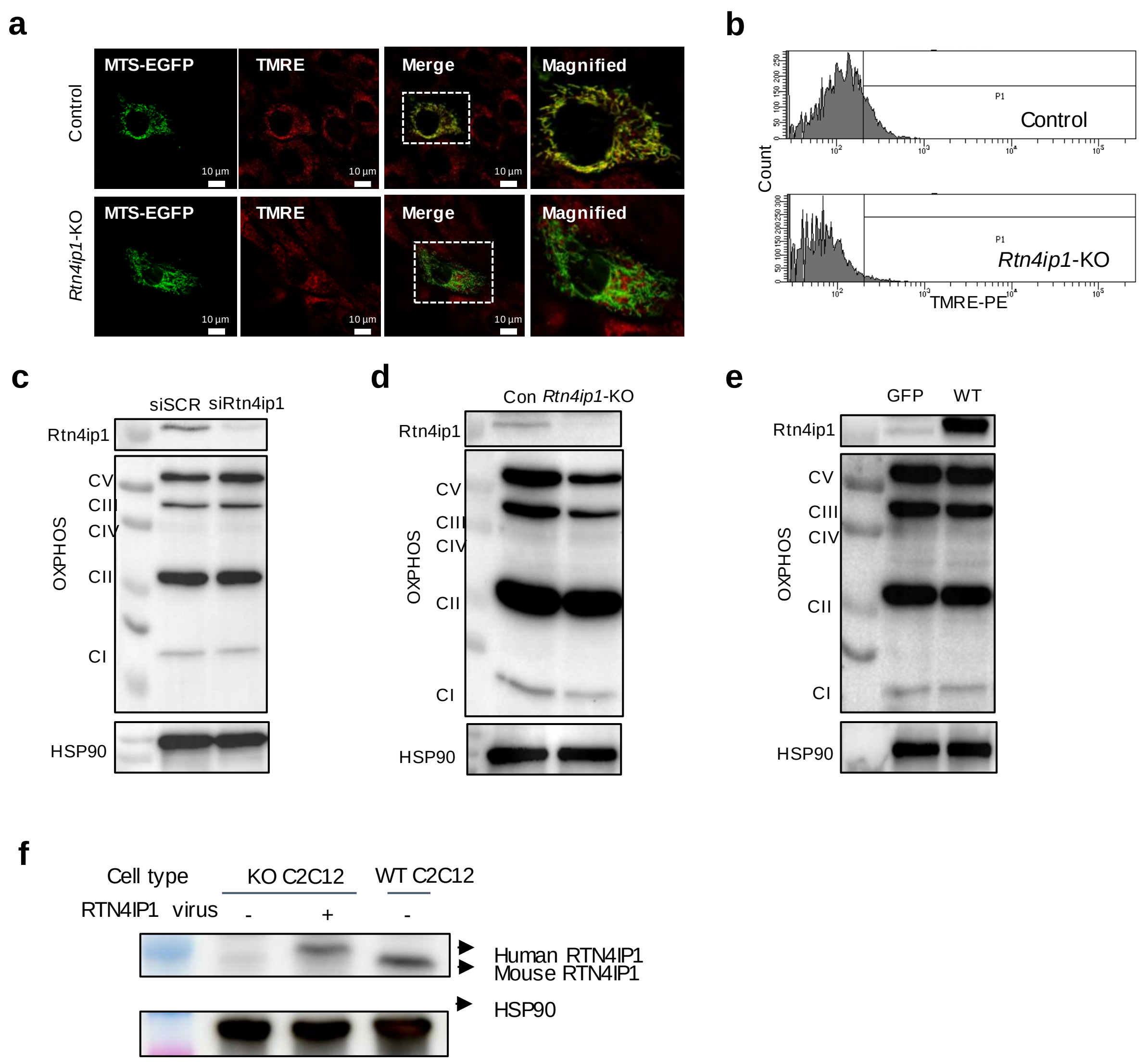

Supplementary Fig. 9 Reduced membrane potential and OXPHOS activity in *Rtn4ip1*-KO cells

(**a, b**) Mitochondrial membrane potential measurement of control and Rtn4ip1-KO C2C12 cells by tetramethylrhodamine, ethyl ester (TMRE) fluorescence and flow cytometry. (**a**) Confocal microscope imaging of TMRE and MTS-EGFP (a mitochondrial marker protein) in control and Rtn4ip1-KO C2C12 cells (scale bar = 10 µm). (**b**) Representative histograms of control and Rtn4ip1-KO C2C12 cells incubated with 1 µM TMRE (left panel); 10,000 cells were assayed for TMRE fluorescence by flow cytometry (**c**) Western blotting with anti-RTN4IP1 and an antibody cocktail for the oxidative phosphorylation (OXPHOS) complex in control and Rtn4ip1-KO C2C12 cells. (**d**) Western blotting with anti-RTN4IP1 and an antibody cocktail for the OXPHOS complex in siSCR and siRtn4ip1 C2C12 cell lysates. Representative images from three independent experiments are shown. (**e**) Western blotting with anti-RTN4IP1 and an antibody cocktail for the OXPHOS complex in GFP or RTN4IP1-overexpressed (RTN4IP1-WT) C2C12 cell lysates. Representative images from three independent experiments are shown. (**f**) Western blotting with anti-RTN4IP1 and an Anti-HSP90 in control and RTN4IP1-rescued C2C12 cell lysates. Representative images from three independent experiments are shown.

ARCHS4 database					
Most similar genes based on co-expression (RTN4IP1)			Most similar genes based on co-expression (COQ3)		
Rank	Gene Symbol	Pearson Correlation	Rank	Gene Symbol	Pearson Correlation
**1**	**COQ3**	**0.687**	**1**	**RTN4IP1**	**0.687**
2	MRPS9	0.654	2	UBE2T	0.686
3	PDCD2L	0.64	3	BOLA3	0.678
4	MRPL39	0.633	4	CHCHD4	0.662
5	MRPL58	0.623	5	MRPS9	0.661
6	NIF3L1	0.613	6	FARSB	0.659
7	CHCHD4	0.609	7	NDUFAF2	0.657
8	SAAL1	0.609	8	PDCD2L	0.657
9	CHAC2	0.603	9	MRPL58	0.657
10	C2orf47	0.6	10	MRPL39	0.656

DepMap 22Q2 Public+Score, Chronos					
Co-dependencies, CRISPR (RTN4IP1)			Co-dependencies, CRISPR (COQ3)		
Rank	Gene Symbol	Pearson Correlation	Rank	Gene Symbol	Pearson Correlation
1	NDUFC1	0.598	1	COA6	0.57
2	FOXRED1	0.565	2	PDSS2	0.556
3	NDUFA1	0.564	**3**	**RTN4IP1**	**0.555**
**4**	**COQ3**	**0.555**	4	NDUFC1	0.543
5	NDUFS8	0.535	5	POLG	0.543
6	DDX28	0.534	6	TFB1M	0.535
7	NDUFAF7	0.531	7	DARS2	0.535
8	NDUFAF3	0.529	8	MTG2	0.529
9	NDUFA2	0.527	9	FOXRED1	0.529
10	NDUFA10	0.527	10	COA7	0.526

Supplementary Table 1 Co-expression and co-dependency values of RTN4IP1 and COQ3.

Co-expression relevance of RTN4IP1-COQ3 in ARCHS4 database (https://maayanlab.cloud/archs4/) (upper), Cancer cell survival co-dependency value between RTN4IP1 and COQ3 from DepMap database (https://depmap.org/portal/)

Gene name	UniProt ID	Mouse Gene ID	MitoCarta3.0 Sublocal	MitoFates Probability	MitoFates prediction	Subcellular localization (UniProt)	Mitoplast Proteome	MAX Proteome	Myf5; MAX Proteome
Sh3bp1	A2A5V3	20401		0.76	Possessing MTS	Cell projection		+	+
Phka1	A2AI91	18679		0.65	Possessing MTS	Cell membrane			+
Zadh2	Q8BGC4	225791	unknown	0.21		Peroxisome	+	+	
Myom2	Q14BI5	17930		0.12				+	+
Spr	Q91XH5	20751	unknown	0.01		Cytoplasm		+	+
Prdx1	P35700	18477				Cytoplasm			+

Supplementary Table 2 Example of newly identified muscle-specific mitochondrial matrix proteins using our in vivo proximity labeling approach (i.e., MAX-Tg mice and Myf5-Cre; LSL-MAX-Tg mice)

Normalized mass intensity: Low – High

Median transcripts per million (TPM): Low – High

	Matrix-APEX2 labeld proteome		Transcriptome (GTEx Portal)													
Gene name	TA muscle	HEK293 cells	Skeletal muscle	Heart	Adipose	Cerebellum	Hippocampus	Kidney	Liver	Lung	Pancreas	Skin	Spleen	Stomach	Testis	Whole Blood
Cox7c	32.93669764	25.67972247	271	314	152.55	170.2	157.8	192.2	111.3	157.1	88.115	141.7	162.1	176.95	139.6	27.8
Ndufa2	32.27803993	26.85724894	57.465	50.6	46.76	47.66	42.19	53.15	38.96	45.09	20.345	36.12	53.35	46.525	79.03	25.1
Cs	32.50312487	29.18262672	236.05	191	124.05	142.4	35.36	68.6	33.42	109.7	46.11	116	82.325	86.3	48.51	33.75
Uqcrc1	32.15523275	28.82472229	361.1	390.1	102.2	154	70.15	137.3	115.7	104.8	53.435	157.3	108.8	167.55	205.7	84.6
Immt	32.421772	27.11312803	103	94.73	57.005	50.8	21.51	50.49	30.81	52.59	24.53	46.87	59.685	54.475	88.87	20.28
Atp5a1	32.27369181	29.5727946	620.1	808	286.2	470.8	157.3	374.7	178.3	268.3	150.65	344.1	297.6	386.25	287.9	108.2
Mdh2	31.88308843	27.9054629	357.6	214.1	98.825	93.37	45.16	92.19	112.2	95.65	57.525	134	101.85	101.15	97.18	38.49
Atp5c1	32.10264015	26.1704038	183.75	297.3	120.4	114.6	70.36	142.5	95.7	111.3	70.605	111.8	101.8	138.15	83.06	31.23
Ndufab1	31.58645058	27.93801181	101.3	157.5	49.365	65.78	41.16	46.37	44.2	48.06	22.54	43.65	48.6	52.535	56.41	9.109

Supplementary Table 3 Transcriptome-level muscle specificity of the muscle-specific mitochondrial matrix proteins identified in this study from TA muscle tissue of MAX-Tg mice.

Transcriptome data of human organs were obtained from the GTEx portal (https://gtexportal.org/)

Molecule name	Molecular formula		Precursor ion m/z		Fragment ion m/z	Mass tolerance of fragment ion	RT (Kinetex column)	CE (Exp480)
DMeQ2	$C_{18}H_{24}O_4$	304.167	$C_{18}H_{24}O_4 + H^+$	305.175	183.066	± 1.00 mDa	2.3	22
CoQ2	$C_{19}H_{26}O_4$	318.183	$C_{19}H_{26}O_4 + H^+$	319.191	197.081	± 1.00 mDa	3.2	22
Reduced CoQ2	$C_{19}H_{28}O_4$	320.199	$C_{19}H_{28}O_4 + H^+$	321.207	197.081	± 1.00 mDa	2.9	25
CoQ9	$C_{54}H_{82}O_4$	794.621	$C_{54}H_{82}O_4 + NH_4^+$	812.656	197.081	± 1.00 mDa	7.8 - 7.9	25
Reduced CoQ9	$C_{54}H_{84}O_4$	796.637	$C_{54}H_{84}O_4 + NH_4^+$	814.671	197.081	± 1.00 mDa	6.3	33
CoQ10	$C_{59}H_{90}O_4$	862.684	$C_{59}H_{90}O_4 + NH_4^+$	880.718	197.081	± 1.00 mDa	10.0 - 10.2	25
Reduced CoQ10	$C_{59}H_{92}O_4$	864.7	$C_{59}H_{92}O_4 + NH_4^+$	882.734	197.081	± 1.00 mDa	7.4 - 7.6	33
13C_6-CoQ9	$^{13}C_6C_{48}H_{82}O_4$	800.621	$^{13}C_6C_{48}H_{82}O_4 + NH_4^+$	818.656	203.081	± 0.02 Da	7.6	35
Reduced 13C_6-CoQ9	$^{13}C_6C_{48}H_{84}O_4$	802.637	$^{13}C_6C_{48}H_{84}O_4 + NH_4^+$	820.671	203.081	± 0.02 Da	6.1	50
13C_6-CoQ10	$^{13}C_6C_{53}H_{90}O_4$	868.684	$^{13}C_6C_{53}H_{90}O_4 + NH_4^+$	886.718	203.081	± 0.02 Da	9.6 - 9.7	35
Reduced 13C_6-CoQ10	$^{13}C_6C_{53}H_{92}O_4$	870.7	$^{13}C_6C_{53}H_{92}O_4 + NH_4^+$	888.734	203.081	± 0.02 Da	7.2	50

Supplementary Table 4 LC-PRM feature parameters for CoQ analysis

Name	Features	Promotor/Vector	Details
MTS-V5-APEX2_pCDNA5	KpnI-MTS-BamHINheI-V5-APEX2-Stop-NotI	CMV/pCDNA5	MTS-V5-APEX2 was a gift from Prof. Alice Ting (Addgene plasmid #72480)
RTN4IP1-V5-APEX2_pCDNA5	AflII-RTN4IP1<br>NheI-V5-APEX2-Stop-NotI	CMV/pCDNA5	NA
MTS(1-32aa of RTN4IP1)-V5-APEX2_pCDNA5	AflII-1-32aa (MTS) of RTN4IP1-<br>NheI-V5-APEX2-Stop-NotI	CMV/pCDNA5	NA
RTN4IP1(ΔMTS)-V5-APEX2_pCDNA5	AflII-RTN4IP1(Δ1-32aa)-NheI- V5-APEX2-Stop-NotI	CMV/pCDNA5	NA
His6_MBP_TEV_ RTN4IP1(Δ1-32aa(MTS))_pET21a	NdeI-His6-NdeI-MBP-EcoRINheI-TEV cleavage site-RTN4IP1(Δ1-32aa)-Stop-XhoI	T7 /pET21a	NA
His6_MBP_TEV_ RTN4IP1(ΔMTS, R103H)-Flag_pET21a	NheI-RTN4IP1 (ΔMTS, R103H)<br>XhoI-Flag-Stop	T7 /pET21a	R103H mutation was added via site-directed mutagenesis.
RTN4IP1(ΔMTS, G215A)-Flag-His6_pET21a	NheI-RTN4IP1 (ΔMTS, G215A)<br>XhoI-Flag-Stop	T7 /pET21a	G215A mutation was added via site-directed mutagenesis.
COQ3(Δ1-85aa(MTS))-Flag-His6_pET21a	NheI-COQ3<br>XhoI-Flag-Stop	T7 /pET21a	COQ3 gene was obtained from KRIBB human gene bank (BKU002093).
RTN4IP1-TurboID_pCDNA5	AflII-RTN4IP1<br>KpnI-V5-TurboID-Stop-XhoI	CMV/pCDNA5	NA
MTS-V5-TurboID_pCDNA5	AflII-MTS-<br>KpnI-V5-TurboID-Stop-XhoI	CMV/pCDNA5	NA

Supplementary Table 5 Construct information

REAGENT or SOURCES	SOURCE	IDENTIFIER
**Antibodies**		
Anti-V5 Tag Monoclonal Antibody (mouse)	Invitrogen	Cat # R960-25
Goat Anti-Mouse IgG (H + L)-HRP conjugate	Bio-Rad Laboratories	Cat # 1706516
Anti-rabbit IgG, HRP-linked Antibody	Cell Signaling Technology	Cat # 7074S
Alexa Fluor 488 IgG mouse	Invitrogen	Cat # A11001
Alexa Fluor 568 IgG mouse	Invitrogen	Cat # A11004
Anti-RTN4IP1	Atlas Antibodies	Cat # HPA036357
Anti-TOM20	ProteinTech	Cat # 11802-1-AP
p44/42 MAPK (Erk1/2)	Cell Signaling Technology	Cat # 9102
Anti-8-OHdG	Santa Cruz Biotechnology	Cat # sc-66036
**Chemicals, Peptides, and Recombinant Proteins**		
Streptavidin-HRP	Thermo Fisher Scientific	Cat # 21126
Streptavidin, Alexa Fluor 647 conjugate	Invitrogen	Cat # S21374
Sodium 2,6-dichloroindophenolate hydrate (DCPIP)	Sigma-Aldrich	Cat # 119814
NADPH	Sigma-Aldrich	Cat # 2646-71-1
Biotin	Alfa Aesar	Cat # A14207
H_2O_2	Sigma-Aldrich	Cat # STBJ2658
RIPA lysis buffer	ELPISBIO	Cat # EBA-1149
Glutaraldehyde	Electron Microscopy Sciences	Cat # 16200
Protease inhibitor cocktail	Invitrogen	Cat # 78438
DAB	Sigma-Aldrich	Cat # D8001
Urea	Sigma-Aldrich	Cat # U5378
Uranyl acetate	Electron Microscopy Sciences	Cat # 22400
Embed-812	Electron Microscopy Sciences	Cat # 14120
Uranyless	Electron Microscopy Sciences	Cat # 22409
Lead citrate	Electron Microscopy Sciences	Cat # 22410
Lipofectamine 2000	Life Technologies	Cat # 11668019
20X TBS	Thermo Fisher Scientific	Cat # 28358
Acetone	Sigma-Aldrich	Cat # 650501
Ammonium bicarbonate	Sigma-Aldrich	Cat # A6141
Doxycycline	Sigma-Aldrich	Cat # D9891
TPCK-Trypsin	Thermo Fisher Scientific	Cat # 20233
Dithiothreitol	Sigma-Aldrich	Cat # 43819
Iodoacetoamide	Sigma-Aldrich	Cat # I1149
$CaCl_2$	Alfa Aesar	Cat # 12312
Trifluoroacetic acid	Sigma-Aldrich	Cat # T6508-10AMP
Formic acid	Thermo Fisher Scientific	Cat # 28905
Acetonitrile	Sigma-Aldrich	Cat # 900667
Coenzyme Q_{10} (oxidized form, >98%)	Sigma-Aldrich	Cat # C9538

Coenzyme Q_9 (oxidized form, >98%)	ChemScene	Cat # CS-6359
Coenzyme Q_{10} (reduced form, >95%)	Biosynth Carbosynth	Cat # FU28634
**Critical Commercial Assays**		
TMRE	Sigma-Aldrich	Cat #115532-52-0
Seahorse XF Cell Mito Stress Test Kit	Agilent	Cat #103015-100
Seahorse XF DMEM medium	Agilent	Cat #103575-100
Seahorse XF 1.0 M glucose solution	Agilent	Cat #103577-100
Seahorse XF 100mM pyruvate solution	Agilent	Cat #103578-100
Seahorse XF 200mM glutamine solution	Agilent	Cat #103579-100
**Oligonucleotides**		
Small interfering RNA (siRNA) for *Rtn4ip1*	Bioneer	Cat # 170728-1
**Recombinant DNA** (See detail information Supplemental Information)		
MTS-V5-APEX2_pCDNA5	Lee et al., 2017	NA
RTN4IP1-V5-APEX2_pCDNA5	This paper	NA
MTS (from RTN4IP1)-V5-APEX2_pCDNA5	This paper	NA
RTN4IP1(Δ1-32aa)-V5-APEX2_pCDNA5	This paper	NA
His6_MBP_TEV_ RTN4IP1(Δ1-32aa)_pET21a	This paper	NA
RTN4IP1-TurboID_pCDNA5	This paper	NA
MTS-V5-TurboID_pCDNA5	This paper	NA
lentiCRISPRv2	Addgene	Cat #52961
**Software and Algorithms**		
Morpheus	Broad Institute	https://software.broadinstitute.org/morpheus/
STRING	CPR, EMBL, SIB, KU, and UZH	https://string-db.org/
MitoFates	AIST	http://mitf.cbrc.jp/MitoFates/cgi-bin/top.cgi
Image J	NIH	https://imagej.nih.gov/ij/
Pymol	Schrodinger	https://pymol.org/2/
Normalizer	Lund University, Medicon Village 406, 223 81, Lund, Sweden	http://normalyzer.immunoprot.lth.se/
Perseus	Max-Planck-Institute of Biochemistry	https://maxquant.net/perseus/

Supplementary Table 6 Material information

Supplementary Video 1. Climbing test

Supplementary Data 1. DBP-labeled peptides and proteins in MAX-Tg

Supplementary Data 2. DBP-labeled proteins by MTS-APEX2 in HEK293T and in muscle tissues

Supplementary Data 3. Tissue-specific DBP-labeled proteins in MAX-Tg

Supplementary Data 4. Muscle tissue-specific DBP-labeled proteins in Myf5-Cre; LSL-MAX-Tg

Supplementary Data 5. DBP-labeled proteins from MAX-Tg and Myf5-Cre; LSL-MAX-Tg mouse and mitoplast proteins from WT mouse

Supplementary Data 6. Interactome of RTN4IP1 revealed by TurboID
