# Supplementary material for: Mitochondrial matrix RTN4IP1/OPA10 is an oxidoreductase for coenzyme Q synthesis
Source: Nat Chem Biol. 2023 Oct 26;20(2):221–33. doi: 10.1038/s41589-023-01452-w (PMC10830421; doi:10.1038/s41589-023-01452-w)
Supplement: Supplementary file 2 — Reporting Summary [file 41589_2023_1452_MOESM2_ESM.pdf]

nature research

Corresponding author(s): Hyun-Woo Rhee

Last updated by author(s): Aug 30, 2023

Reporting Summary

Nature Research wishes to improve the reproducibility of the work that we publish. This form provides structure for consistency and transparency in reporting. For further information on Nature Research policies, see our Editorial Policies and the Editorial Policy Checklist.

Statistics

For all statistical analyses, confirm that the following items are present in the figure legend, table legend, main text, or Methods section.

n/a	Confirmed	
☐	☒	The exact sample size (*n*) for each experimental group/condition, given as a discrete number and unit of measurement
☐	☒	A statement on whether measurements were taken from distinct samples or whether the same sample was measured repeatedly
☐	☒	The statistical test(s) used AND whether they are one- or two-sided<br>*Only common tests should be described solely by name; describe more complex techniques in the Methods section.*
☐	☒	A description of all covariates tested
☒	☐	A description of any assumptions or corrections, such as tests of normality and adjustment for multiple comparisons
☐	☒	A full description of the statistical parameters including central tendency (e.g. means) or other basic estimates (e.g. regression coefficient) AND variation (e.g. standard deviation) or associated estimates of uncertainty (e.g. confidence intervals)
☐	☒	For null hypothesis testing, the test statistic (e.g. *F*, *t*, *r*) with confidence intervals, effect sizes, degrees of freedom and *P* value noted<br>*Give P values as exact values whenever suitable.*
☒	☐	For Bayesian analysis, information on the choice of priors and Markov chain Monte Carlo settings
☐	☒	For hierarchical and complex designs, identification of the appropriate level for tests and full reporting of outcomes
☐	☒	Estimates of effect sizes (e.g. Cohen's *d*, Pearson's *r*), indicating how they were calculated

Our web collection on statistics for biologists contains articles on many of the points above.

Software and code

Policy information about availability of computer code

Data collection	Thermo Xcalibur (version 4.3 and version 4.4) was used for MS data acquisition.
Data analysis	MaxQuant (version 1.5.3.30 and version 1.6.2.3 for MTS-APEX2 mouse experiment) and the Andromeda search algorithm were used for MS data analysis. The mass spectrometry proteomics data have been deposited to the ProteomeXchange Consortium (http://proteomecentral.proteomexchange.org) via the PRIDE partner repository with the dataset identifier PXD026793.<br>Intensities in the MTS-APEX2 mouse experiment, were quantile normalized using Normalyzer (http://quantitativeproteomics.org/normalyzer).<br>Hierarchical clustering was performed with Morpheus (8331b8d on Jul 15, 2022 Git stats, https://software.broadinstitute.org/morpheus/)

For manuscripts utilizing custom algorithms or software that are central to the research but not yet described in published literature, software must be made available to editors and reviewers. We strongly encourage code deposition in a community repository (e.g. GitHub). See the Nature Research guidelines for submitting code & software for further information.

Data

Policy information about availability of data

All manuscripts must include a data availability statement. This statement should provide the following information, where applicable:

- Accession codes, unique identifiers, or web links for publicly available datasets
- A list of figures that have associated raw data
- A description of any restrictions on data availability

The structural information was referred to from the Protein Data Bank under the following accession numbers: 2VN8, 1QOR. The co-expression relevance data for RTN4IP1-COQ3 was retrieved from the ARCHS4 database (https://maayanlab.cloud/archs4/) and the DepMap database (https://depmap.org/portal/). Transcriptome data for human organs were acquired from the GTEx portal (https://gtexportal.org/). For comparing annotation of sub-mitochondrial localization, Mouse MitoCarta3.0 datasets and UniProt database (https://www.uniprot.org/) were used. Further information and requests for resources and reagents should be

directed to and will be fulfilled by the lead contact, Hyun-Woo Rhee (rheehw@snu.ac.kr). Upon reasonable request, unique reagents utilized in this paper can be provided.

Field-specific reporting

Please select the one below that is the best fit for your research. If you are not sure, read the appropriate sections before making your selection.

☒ Life sciences ☐ Behavioural & social sciences ☐ Ecological, evolutionary & environmental sciences

For a reference copy of the document with all sections, see nature.com/documents/nr-reporting-summary-flat.pdf

Life sciences study design

All studies must disclose on these points even when the disclosure is negative.

Sample size	For mouse experiments, three mice were utilized for each experimental group. The determination of the sample size was based on achieving the minimum number of animals required for sacrifice while also ensuring statistical validity. The figure legend provides information about the sample size for the experiment and all the statistical analyses performed.
Data exclusions	There were no data exclusions except those resulting from technical errors making data interpretation impossible.
Replication	The replication of proteomic experiments is summarized in figure legend or supplementary information. Other experiments were confirmed with multiple biological replicates as indicated in the figure legends, and the representative results are shown.
Randomization	The mice, cells, and flies used in this study were allocated randomly. They were selected for transmission electron microscopy (TEM) or fluorescence imaging through random selection. For lysis and western blotting, mice and cells were also randomly chosen, including for mass spectrometry (MS) analysis.
Blinding	Sample treatments and subsequent MS sample processing were blinded from one another. No additional blinding was employed in order to make comparisons between specific treatments.

Reporting for specific materials, systems and methods

We require information from authors about some types of materials, experimental systems and methods used in many studies. Here, indicate whether each material, system or method listed is relevant to your study. If you are not sure if a list item applies to your research, read the appropriate section before selecting a response.

Materials & experimental systems

n/a	Involved in the study	
☐	☒	Antibodies
☐	☒	Eukaryotic cell lines
☒	☐	Palaeontology and archaeology
☐	☒	Animals and other organisms
☒	☐	Human research participants
☒	☐	Clinical data
☒	☐	Dual use research of concern

Methods

n/a	Involved in the study	
☒	☐	ChIP-seq
☐	☒	Flow cytometry
☒	☐	MRI-based neuroimaging

Antibodies

Antibodies used	Anti-V5 Tag Monoclonal Antibody (mouse) (Invitrogen, R960-25, 1:1000)<br>HRP-conjugated goat anti-rabbit IgG (H+L) (Cell Signaling Technology, 7074S, 1:1000)<br>HRP-conjugated goat anti-mouse IgG (H+L) (Bio-Rad Laboratories, 1706516, 1:1000)<br>Alexa Fluor 488 IgG mouse (Invitrogen, A11001, 1:10000)<br>Alexa Fluor 568 IgG mouse (Invitrogen, A11004, 1:10000)<br>Anti-RTN4IP1 rabbit (Atlas Antibodies, HPA036357, 1:1000)<br>Anti-TOM20 rabbit (ProteinTech, 11802-1-AP, 1:3000)<br>Rabbit anti-ERK1/2 (Cell Signaling Technologies, 4695, 1:2000)<br>Anti-8-OHdG (Santa Cruz Biotechnology, SC66036, 1:3000)<br>Anti-Laminin (Abcam, ab11575, 1:2000)
Validation	All antibodies were used for applications validated by antibody suppliers per quality assurance provided by each supplier.<br>Anti-V5 Tag Monoclonal Antibody (mouse) was validated for Western Blot (WB), Immunocytochemistry (ICC/IF), ELISA (ELISA), Immunoprecipitation (IP). (https://www.thermofisher.com/antibody/product/V5-Tag-Antibody-clone-SV5-Pk1-Monoclonal/R960-25)<br>HRP-conjugated goat anti-rabbit IgG (H+L) was validated in human and mouse species. (https://www.cellsignal.com/products/secondary-antibodies/anti-rabbit-igg-hrp-linked-antibody/7074?_requestid=3041343)<br>HRP-conjugated goat anti-mouse IgG (H+L) was validated in human and mouse species. (https://www.thermofisher.com/antibody/

product/Goat-anti-Mouse-IgG-H-L-Secondary-Antibody-Polyclonal/31430)
Alexa Fluor 488 IgG mouse was validated for Immunohistochemistry (IHC), Immunocytochemistry (ICC/IF), Flow Cytometry (Flow). (https://www.thermofisher.com/antibody/product/Goat-anti-Mouse-IgG-H-L-Cross-Adsorbed-Secondary-Antibody-Polyclonal/A-11001)
Anti-RTN4IP1 rabbit was validated for Immunohistochemistry (IHC), Western Blot (WB) in Human species. (https://www.atlasantibodies.com/products/antibodies/primary-antibodies/triple-a-polyclonals/rtn4ip1-antibody-hpa036357/)
Anti-TOM20 rabbit was validated for Immunohistochemistry (IHC), Western Blot (WB), Immunofluorescence (IF), Immunoprecipitation (IP) in Human and Mouse species. (https://www.ptglab.com/products/TOM20-Antibody-11802-1-AP.htm)
Rabbit anti-ERK1/2 was validated for Immunohistochemistry (IHC), Western Blot (WB), Immunofluorescence (IF), Immunoprecipitation (IP), Flow Cytometry. (https://www.cellsignal.com/products/primary-antibodies/p44-42-mapk-erk1-2-137f5-rabbit-mab/4695)
Anti-8-OHdG was validated for Immunohistochemistry (IHC), Immunofluorescence (IF). (https://www.scbt.com/p/8-ohdg-antibody-15a3)
Anti-Laminin was validated for Immunohistochemistry (IHC) in Human and Mouse species. (https://www.abcam.com/products/primary-antibodies/laminin-antibody-ab11575.html)

Eukaryotic cell lines

Policy information about cell lines

Cell line source(s)	HEK293T and C2C12 cells were obtained from the American Type Culture Collection (ATCC).
Authentication	The cell lines were frequently checked by their morphological features and the cell lines are not been authenticated by the short tandem repeat (STR) profiling.
Mycoplasma contamination	All cell lines were tested to be mycoplasma-negative by the standard PCR method.
Commonly misidentified lines (See ICLAC register)	No commonly misidentified cell lines are used in this study.

Animals and other organisms

Policy information about studies involving animals; ARRIVE guidelines recommended for reporting animal research

Laboratory animals	MTS-V5-APEX2 Tg mice (based on C57BL/6N) were generated, interbred in specific pathogen-free conditions at Macrogen (Seoul, Republic of Korea). Mice were maintained under a 12 h light-dark cycle in a climate-controlled (22°C with 50% humidity) specific pathogen-free facility in Seoul National University. Standard chow diet and water were provided ad libitum. 8 weeks old mice were used for the experiment.
Wild animals	no wild animals were used in the study.
Field-collected samples	no field collected samples were used in the study.
Ethics oversight	Animal studies were approved by the Institutional Animal Care and Use Committee of Seoul National University (no SNU180521-2-1). Mice were maintained in a specific pathogen-free institute.

Note that full information on the approval of the study protocol must also be provided in the manuscript.

Flow Cytometry

Plots

Confirm that:

☒ The axis labels state the marker and fluorochrome used (e.g. CD4-FITC).

☒ The axis scales are clearly visible. Include numbers along axes only for bottom left plot of group (a 'group' is an analysis of identical markers).

☒ All plots are contour plots with outliers or pseudocolor plots.

☒ A numerical value for number of cells or percentage (with statistics) is provided.

Methodology

Sample preparation	Control or Rtn4ip1-KO C2C12 cells were cultured in an incubator (37°C, 5% CO2). For flow cytometry, the cells were harvested by trypsin treatment, and then resuspended in 0.5 mL DMEM supplemented with 5% FBS containing 200 nM of TMRE for 30 min at 37°C. Cellular fluorescence was measured using the Flow Activated Cell Sorter (FACS CantoII, TMRE-PE). Data were analyzed by BD FACSDiva™ Software.
Instrument	Flow Activated Cell Sorter (FACS CantoII)
Software	Data were analyzed by BD FACSDiva™ Software

Cell population abundance	10,000
Gating strategy	Samples were analyzed by BD FACS CantoII with the proper lasers and plotted by FSC-A and PE-A. For population P1, a gate was drawn between 200 - 10^5 PE-A of cell clusters.

☒ Tick this box to confirm that a figure exemplifying the gating strategy is provided in the Supplementary Information.
