# Supplementary figures and images for: Mitochondrial matrix RTN4IP1/OPA10 is an oxidoreductase for coenzyme Q synthesis
Source: Nat Chem Biol. 2023 Oct 26;20(2):221–33. doi: 10.1038/s41589-023-01452-w (PMC10830421; doi:10.1038/s41589-023-01452-w)

Fig1b

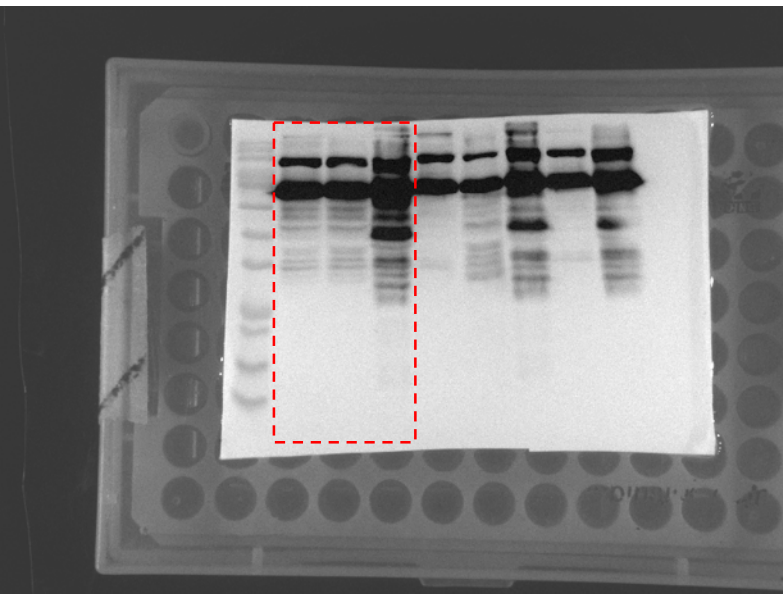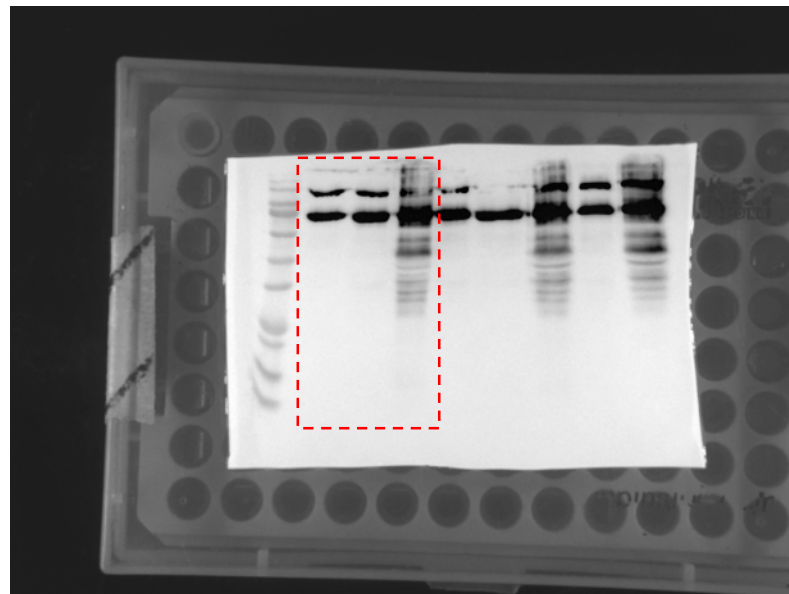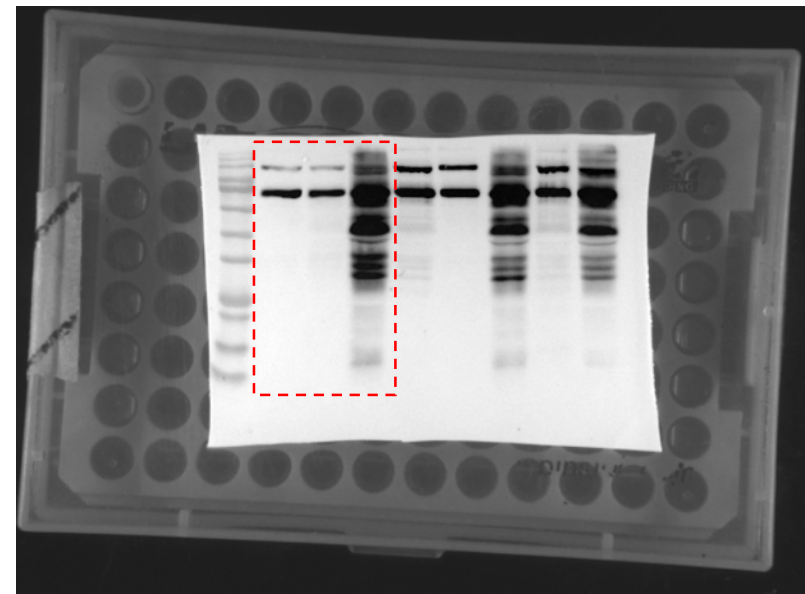

Fig1c

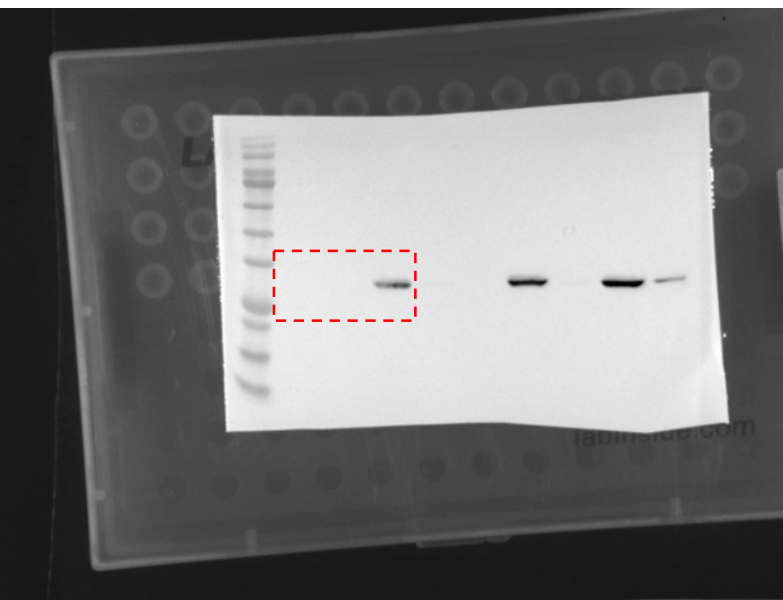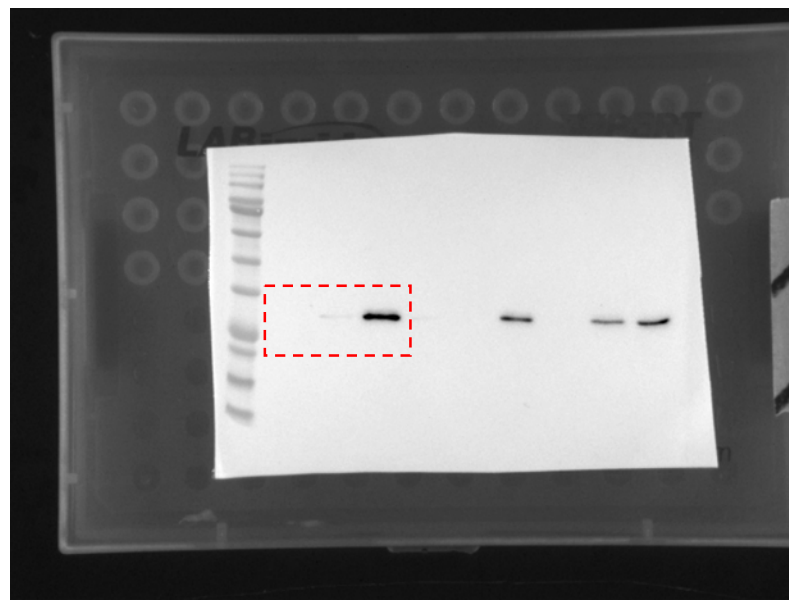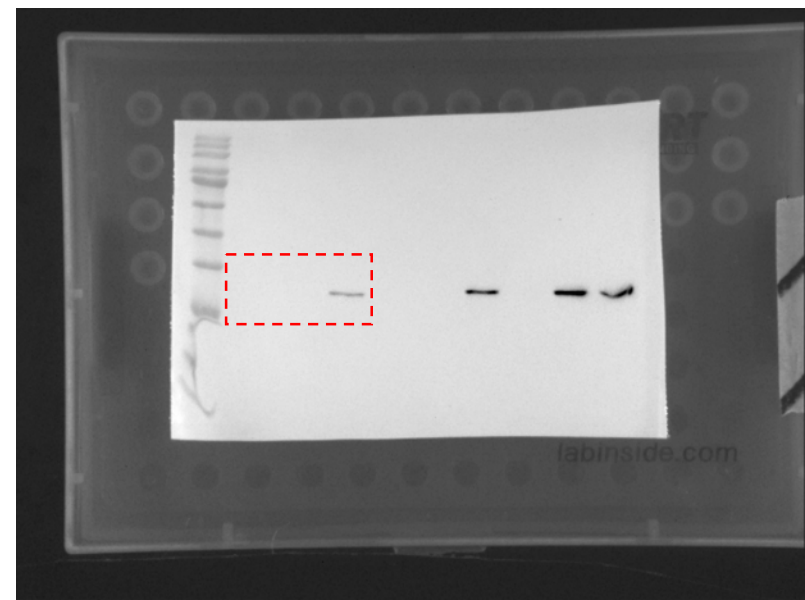

Supplement: Supplementary file 10 — Unmodified western blot. [file 41589_2023_1452_MOESM10_ESM.pdf]

Fig5g

Anti-HSP90

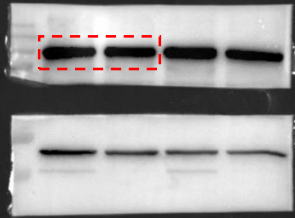

OXPHOS

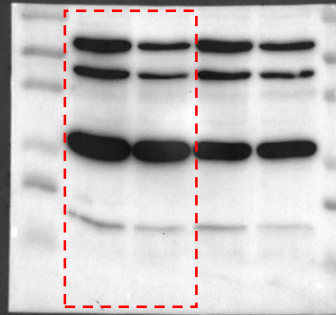

Anti-RTN4IP1

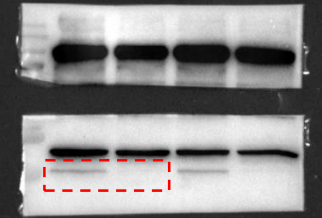

Supplement: Supplementary file 14 — Unmodified western blot. [file 41589_2023_1452_MOESM14_ESM.pdf]

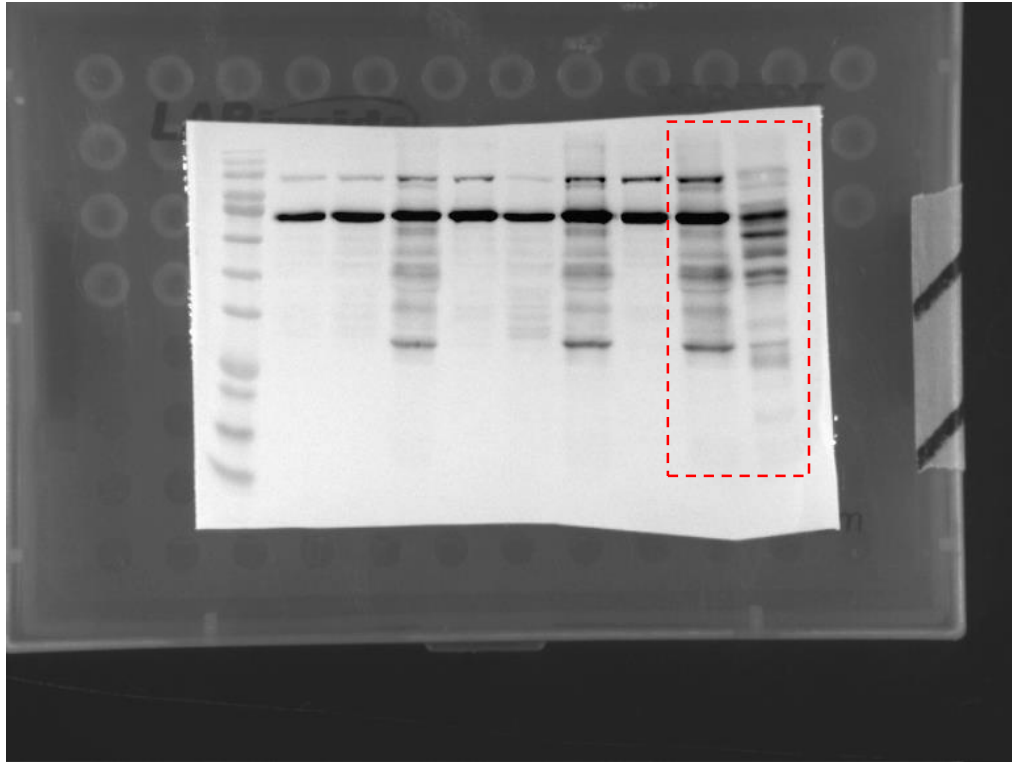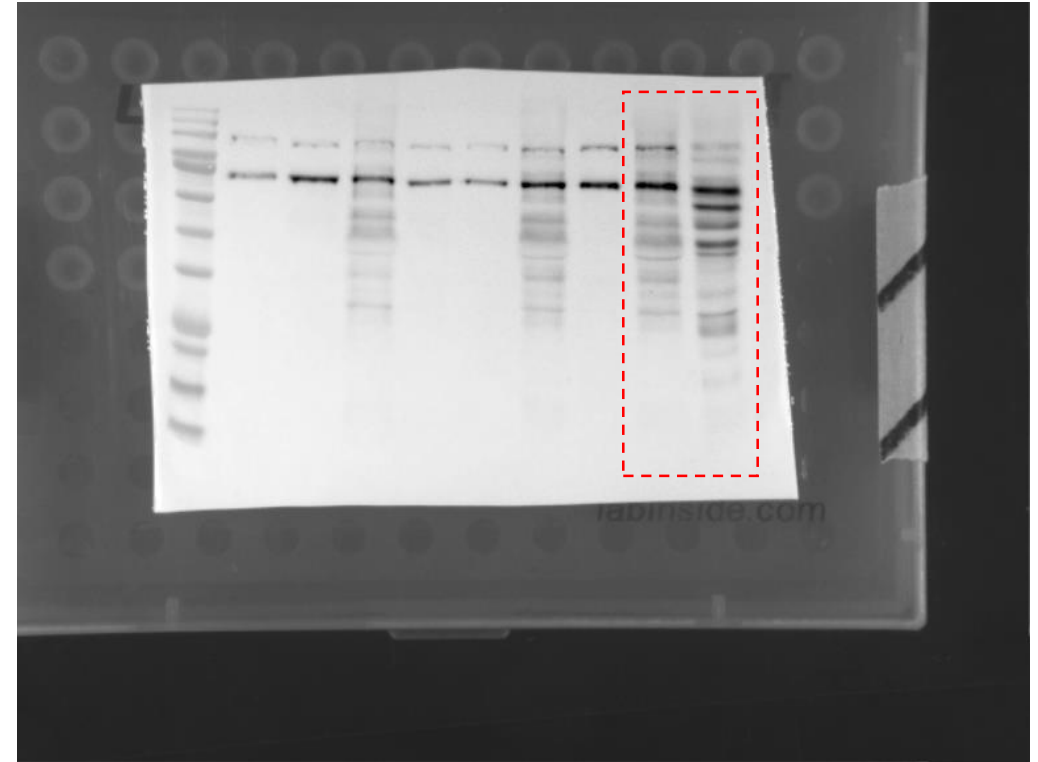

Supplement: Supplementary file 16 — Unmodified western blot. [file 41589_2023_1452_MOESM16_ESM.pdf]

ExFig3b

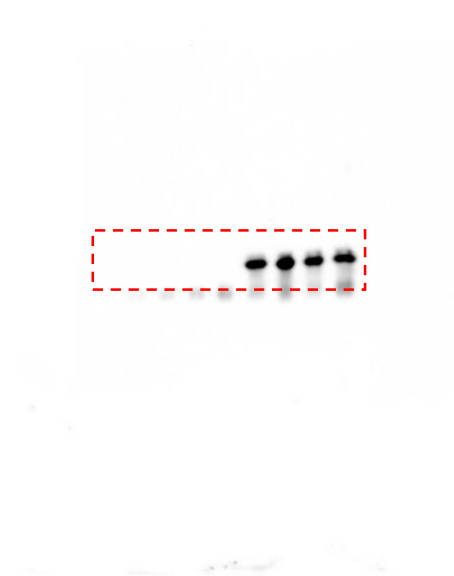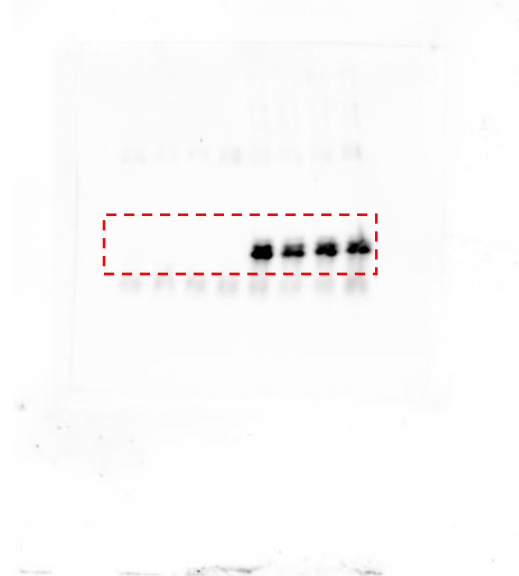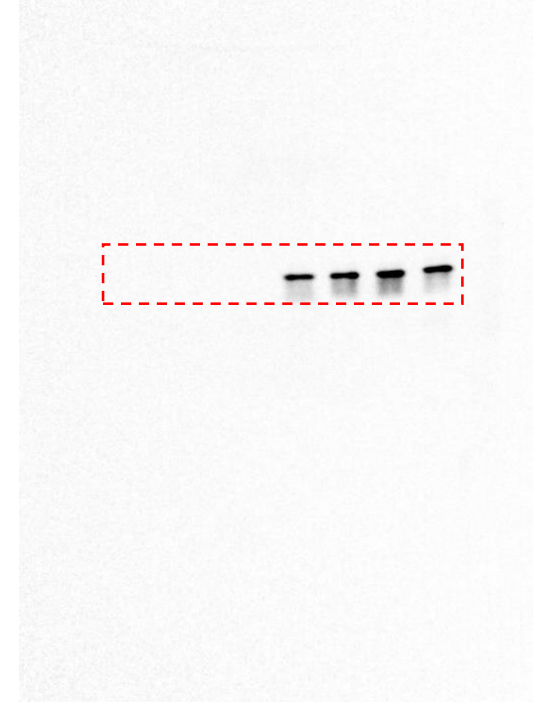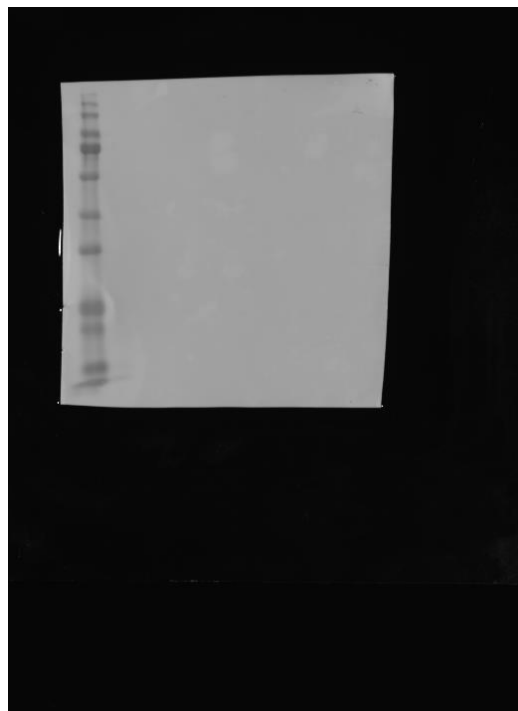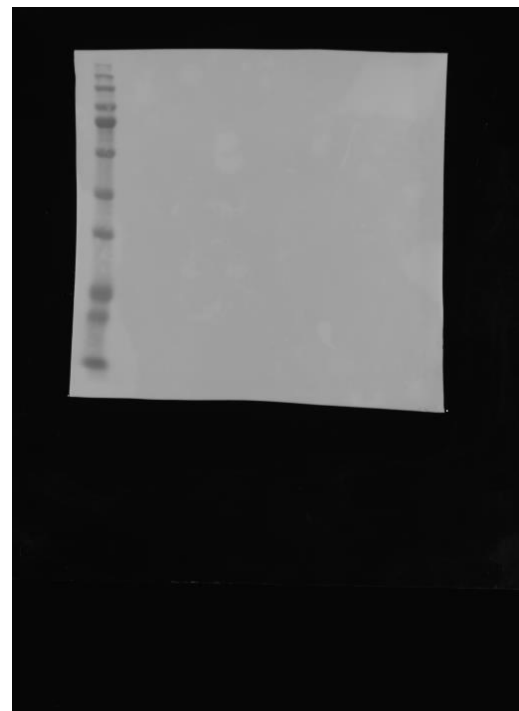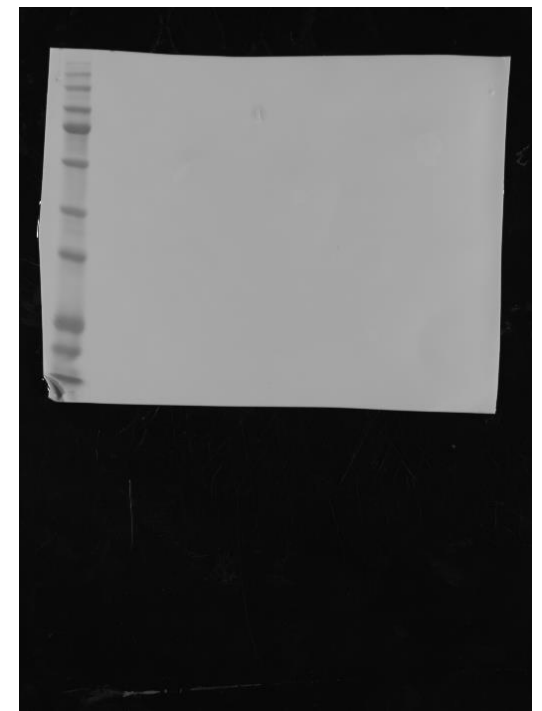

ExFig3c

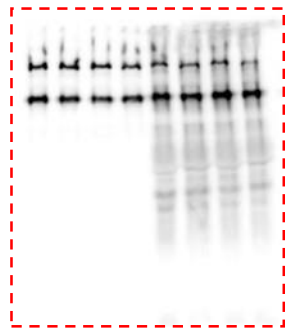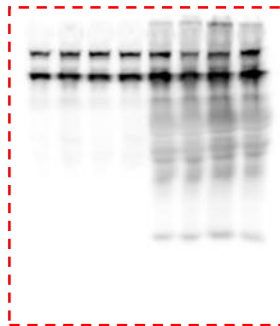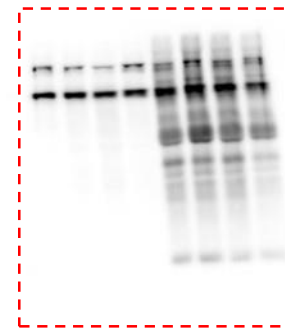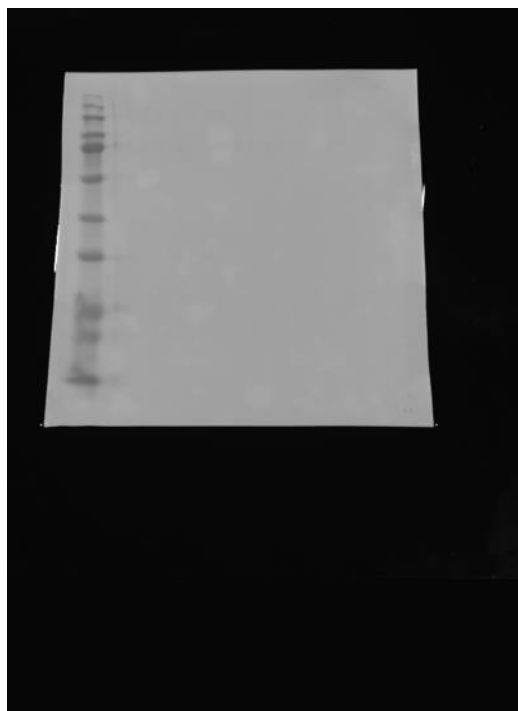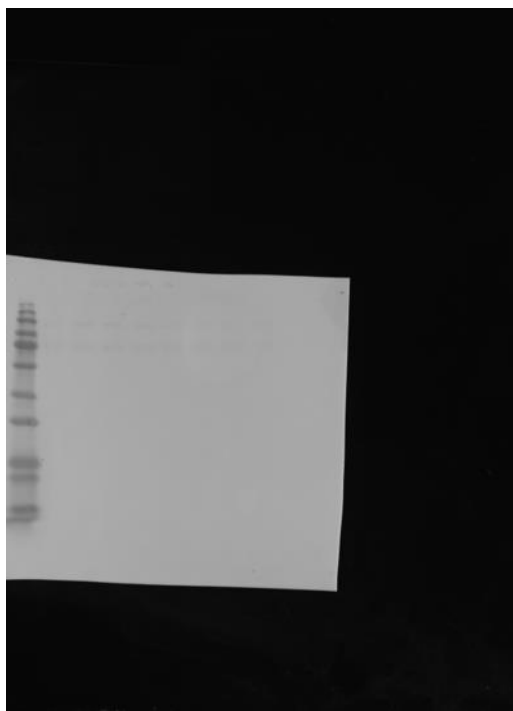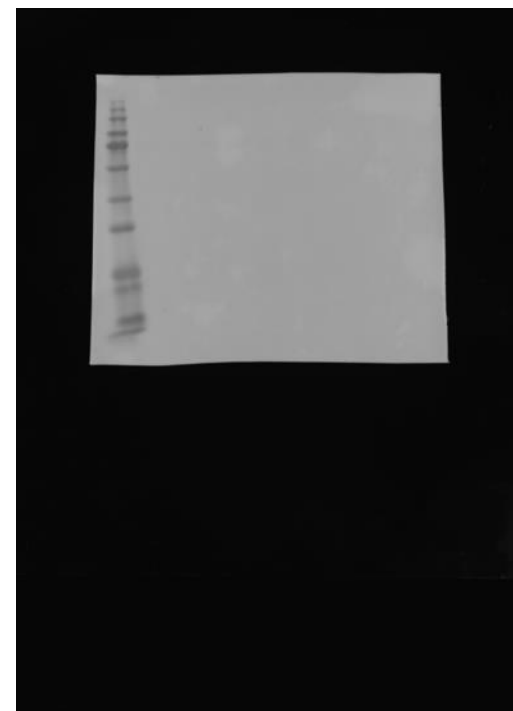

Supplement: Supplementary file 17 — Unmodified western blot. [file 41589_2023_1452_MOESM17_ESM.pdf]

Anti-Gapdh (Cytosol)

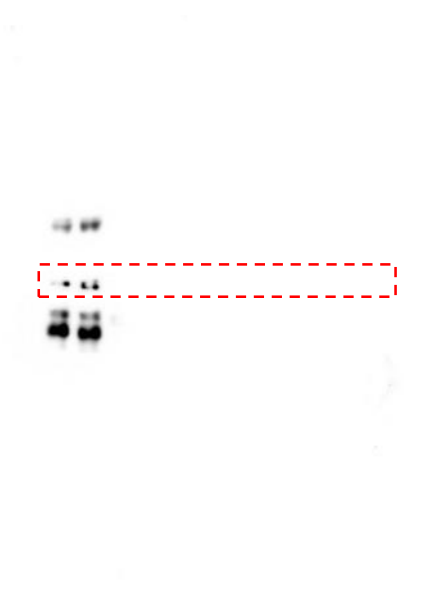

Anti-Tom20 (OMM)

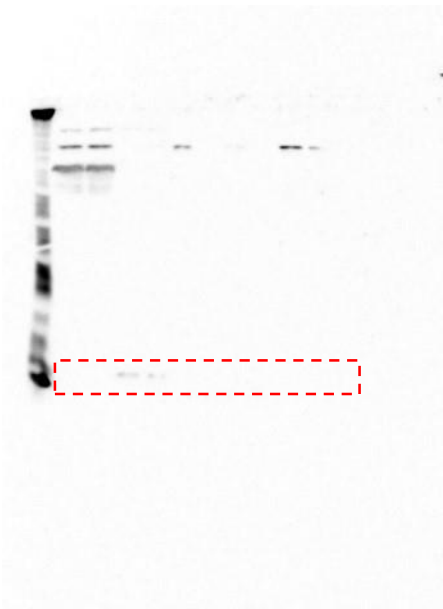

Anti-Chchd3 (IMS)

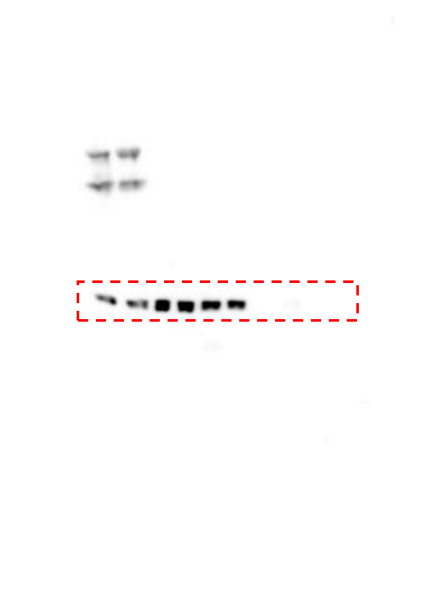

Anti-Hspd1 (Matrix)

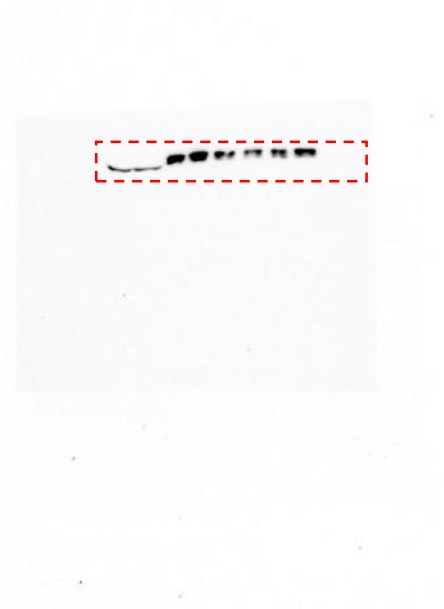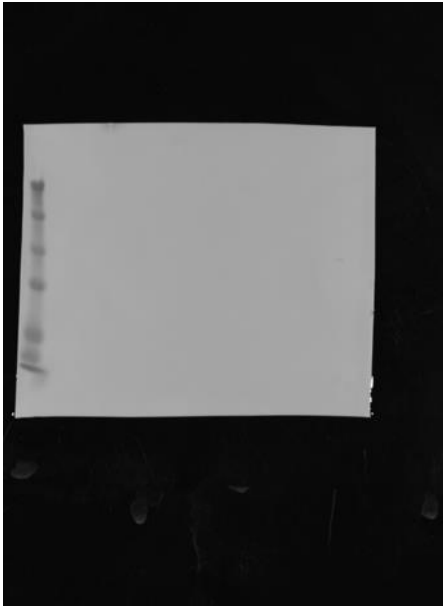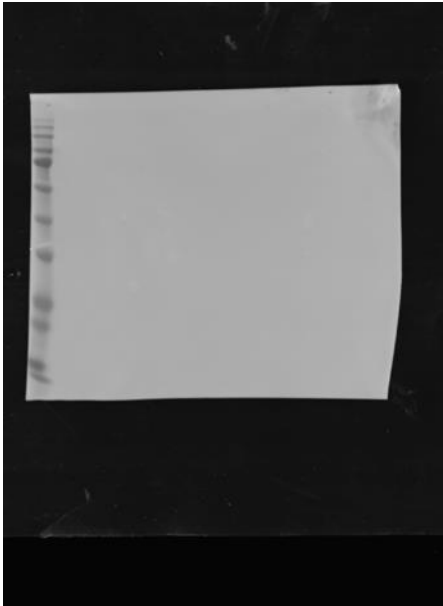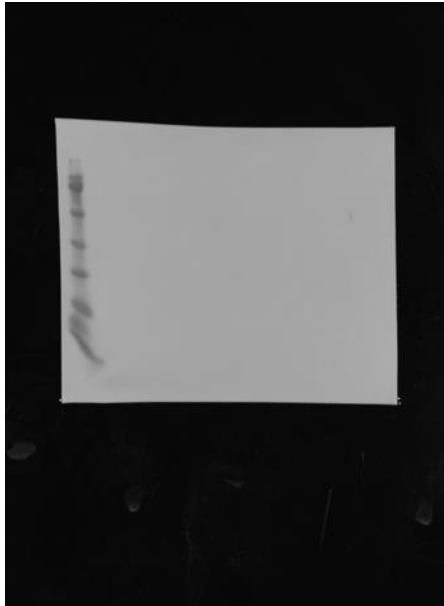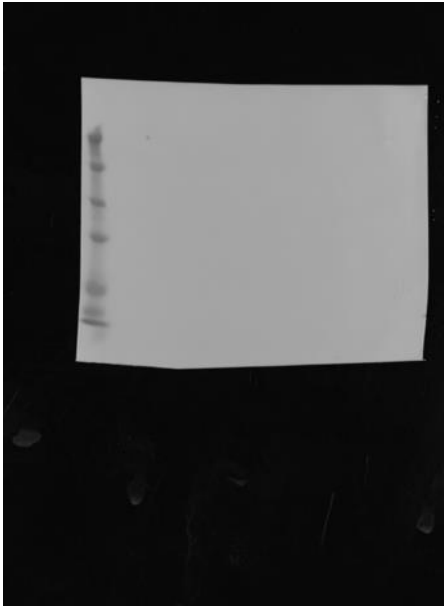

Supplement: Supplementary file 18 — Unmodified western blot. [file 41589_2023_1452_MOESM18_ESM.pdf]
